# Long-term effects of preterm birth on cortical folding trajectories in early childhood

**DOI:** 10.1093/braincomms/fcag097

**Published:** 2026-05-18

**Authors:** Yong Hun Jang, Jong Min Kim, Bong Gun Lee, Jeong-Kyu Hoh, Gang Yi Lee, Hyun Ho Kim, Ilwoo Lyu, Hyun Ju Lee

**Affiliations:** Department of Paediatrics, Hanyang University Hospital, Hanyang University College of Medicine, Seoul 04763, Republic of Korea; Graduate School of Artificial Intelligence, POSTECH, Pohang 37673, South Korea; Department of Orthopaedic Surgery, Hanyang University Hospital, Hanyang University College of Medicine, Seoul 04763, Republic of Korea; Department of Obstetrics and Gynaecology, Hanyang University Hospital, Hanyang University College of Medicine, Seoul 04763, Republic of Korea; Department of Translational Medicine, Hanyang University Graduate School of Biomedical Science and Engineering, Seoul 04763, Republic of Korea; Department of Paediatrics, Jeonbuk National University School of Medicine, Jeonju 54896, Republic of Korea; Graduate School of Artificial Intelligence, POSTECH, Pohang 37673, South Korea; Department of Computer Science and Engineering, POSTECH, Pohang 37673, South Korea; Department of Paediatrics, Hanyang University Hospital, Hanyang University College of Medicine, Seoul 04763, Republic of Korea; Hanyang Institute of Bioscience and Biotechnology, Hanyang University, Seoul 04763, Republic of Korea

**Keywords:** preterm infants, early childhood, cortical folding, local gyrification index, sulcal depth

## Abstract

Cortical folding emerges in the late prenatal period and undergoes rapid reorganization during early childhood. However, the long-term impact of folding alterations associated with preterm birth remains unclear. Herein, we analysed the structural MRI data of 56 preterm children and 206 full-term peers aged 1–7 years. We derived cortical metrics from the reconstructed cortical surfaces using a vertex-wise computation framework to characterize regional folding patterns. We then conducted a combined analysis of the local gyrification index and sulcal depth to explain folding patterns in the preterm brain. Compared with their full-term peers, preterm children exhibited a region-specific impairment pattern characterized by a significantly reduced local gyrification index and sulcal depth in the bilateral superior temporal gyrus and left superior frontal gyrus (*P* < 0.05). Notably, the sulcal depth in the superior temporal cortex showed significant differences between preterm and full-term children in its association with neurodevelopmental outcomes (*P* < 0.05), indicating an atypical structure–function relationship in preterm children. The local gyrification index was significantly reduced in the right isthmus cingulate and posterior cingulate gyri (*P* < 0.05), reflecting a simplified gyral configuration. The study findings suggest several folding patterns that capture diverse mechanisms of morphogenetic disruption, indicating that preterm birth induces persistent region-specific impairments in cortical folding that may affect neurodevelopmental domains. These folding-sensitive markers provide critical insights into the development of targeted interventions to optimize long-term neurodevelopmental outcomes.

## Teaser

Jang and Kim *et al*. report that the study aimed to characterize cortical folding in early childhood using a vertex-wise computational framework. They found reductions in local gyrification index and sulcal depth in superior temporal and frontal regions, with reduced local gyrification index in the isthmus and posterior cingulate in preterm children.

## Graphical Abstract

**Graphical Abstract:**
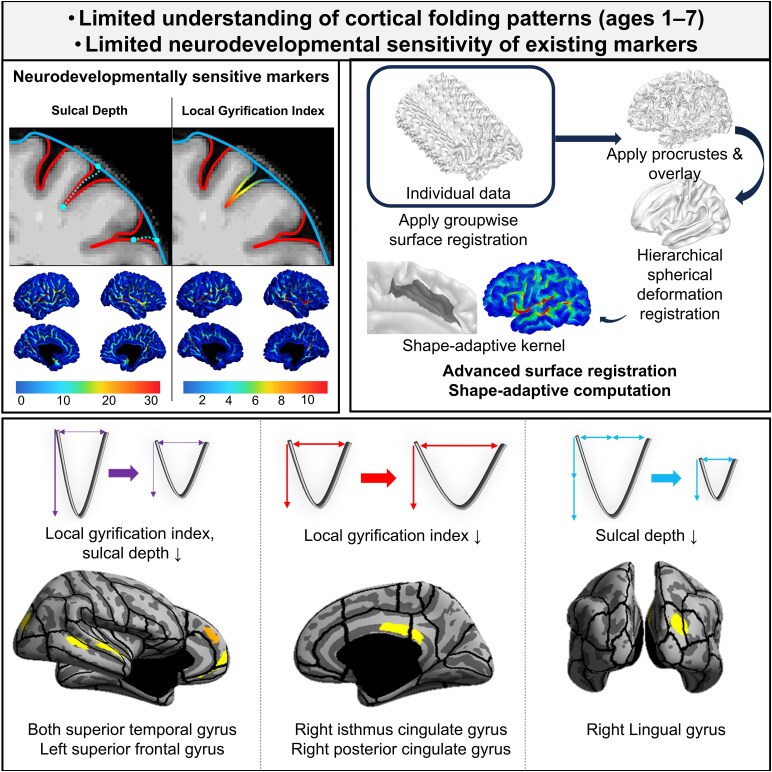

## Introduction

Cortical folding in the human brain occurs during the third trimester of gestation and continues throughout infancy and early childhood. Dynamic and progressive folding processes are orchestrated by a complex interaction of genetic programmes, mechanical forces and environmental inputs, facilitating initial cognitive development.^1,2^ Despite notable inter-individual variability, sulcal emergence follows a highly stereotyped gene-driven spatiotemporal sequence that is conserved across individuals.^3-10^ During the third trimester, primary sulci such as the precentral, calcarine and cingulate sulci emerge rapidly, followed by secondary and tertiary folding at approximately 32 and 38 weeks postmenstrual age, respectively.^11^ Leading theoretical models have proposed that cortical folding is either induced by axonal tension along long-range white matter tracts^12^ or continuously modified by local mechanical instability arising from region-specific heterogeneous growth in the outer cortical layers.^13,14^ Longitudinal cohort studies have shown that the global gyrification index (GI) increases by approximately 23.7% between term-equivalent age and age 2 in typically developing children,^15^ with annual increases in GI falling below 0.5% after age 6, suggesting that gyrification may reach its peak before age 6.^16-18^

The emergence of major folding coincides with the period of preterm birth, raising concerns about the vulnerability of cortical folding processes to extrauterine environmental perturbations. At term-equivalent age, preterm infants exhibit fewer complex patterns of secondary and tertiary folding than do full-term (FT) infants, indicating an increased vulnerability of cortical folding to environmental morphogenetic factors during this sensitive period.^11^ Early disruption of cortical folding may impair the structural coupling between cortical folding complexity and long-range white matter connectivity, thereby altering subsequent regional specialization during neurodevelopment.^19,20^ Such deviations from the normative folding trajectories have been consistently linked to long-term cortical dysmaturation and cognitive impairments.^21-24^

MRI studies comparing preterm (PT) and FT individuals have consistently revealed alterations in the spatial patterns of gene expression^25^ and cortical microstructures that persist from term-equivalent age through childhood.^26-29^ However, elucidating the specific effects of extrauterine exposure on regional brain development remains challenging due to the multifactorial interplay between genetic, epigenetic and environmental factors. Recent longitudinal studies examining cortical development in PT versus FT populations have typically relied on macrostructural metrics, such as surface area, cortical thickness and volume.^28,30-35^ While these metrics offer valuable insights into group-level anatomical differences and general developmental trends, they are limited in their capacity to comprehensively capture the region-specific variability of early cortical development, morphological complexity of long-term cortical maturation, and associated pathological risk factors. Furthermore, existing theoretical frameworks on cortical folding have predominantly emphasized the early postnatal period (0–2 years of age), with comparatively less attention paid to the subsequent phase of accelerated brain reorganization that occurs between 2 and 7 years of age.^15,36^ Given that ongoing brain structural changes and the emergence of various cognitive functions occur from 1 to 7 years of age,^37^ the present study aimed to clarify the spatiotemporal differences between PT and FT folding patterns.

In light of this need, the present study investigated cortical maturation from 1 to 7 years of age following PT birth within a biomechanical framework, focusing on two shape-sensitive markers of cortical folding: the local gyrification index (LGI) and sulcal depth (SD). The combination of LGI and SD has emerged as a clinically interpretable measure that offers sensitivity to developmental and pathological variations that may not be reflected in conventional metrics.^38^ From a macro perspective, LGI reflects the proportion of the buried cortex within a given region, capturing local folding complexity,^39^ while SD quantifies sulcal invagination to detect subtle morphological changes.^9^ Specifically, these morphological indices are particularly responsive to disruptions in early neurodevelopmental processes, including neuronal migration, cortical lamination and white matter expansion,^40^ and later refinements, such as dendritic arborization and thalamocortical innervation.^41-43^ These measures reflect cortical complexity that may be associated with differential function, given that cortical folding patterns are influenced by the underlying cytoarchitecture and neural connections. Furthermore, LGI and SD are highly sensitive to structural alterations in regions influenced by postnatal environmental factors and white matter expansion^15,44^ and have validated the utility of mapping regionally specific maturation patterns across early developmental windows.^45-49^ Moreover, aberrant patterns of cortical folding have been increasingly reported in individuals with psychiatric disorders such as schizophrenia,^50^ bipolar disorder,^51^ depression^52^ and anxiety.^53^ These findings suggest that deviations in early sulcal formation, driven by both genetic programming and environmental perturbations during foetal and infant neurodevelopment, may induce mechanical disequilibrium in cortical morphogenesis, thereby increasing vulnerability to later psychiatric disorders. Taken together, these findings underscore the relevance of folding-based morphometrics as biomarkers of atypical neurodevelopmental trajectories.

This study was based on the hypothesis that children born PT would exhibit altered trajectories of cortical folding between the ages of 1 and 7 years due to PT birth-related disruptions in the cortical architecture. To examine this, we evaluated a cross-sectional cohort comprising 56 PT and 206 FT children by extracting LGI and SD, along with assessing their neurodevelopmental outcomes. Technically, we utilized a shape-adaptive LGI measure to increase the sensitivity to microstructural alterations during periods of accelerated cortical reorganization and interpreted it in conjunction with SD. Furthermore, we identified the associations between cortical metrics and neurodevelopmental outcomes to provide clinically meaningful insights.

## Materials and methods

### Study populations

The present study included PT and FT participants aged 1–7 years who were recruited from two independent hospital cohorts for a cross-sectional analysis. A total of 37 PT infants born at <37 weeks’ gestational age (GA) were admitted to the neonatal intensive care unit of Hanyang University Hospital and prospectively enrolled in a follow-up project at the Hanyang Inclusive Clinic for Developmental Disorders between 2017 and 2022. Another cohort of 26 PT infants was retrospectively recruited from the neonatal intensive care unit of the Jeonbuk National University Hospital between 2017 and 2021. For the FT group, 86 infants born at ≥37 weeks of GA were prospectively recruited within 1 week of birth from the newborn nursery at Hanyang University Hospital, and 131 typically developing children were retrospectively recruited from Jeonbuk National University Hospital between 2017 and 2024. The cohorts from both institutions were recruited at tertiary care centres within South Korea’s National Health Insurance system, and they shared similar socio-economic conditions in access to neonatal and long-term developmental services. PT infants with known severe bronchopulmonary dysplasia, congenital brain abnormalities, congenital infections, cystic periventricular leukomalacia, diffuse ventriculomegaly, genetic disorders (clinically or radiologically suspected), focal brain lesions, intraventricular haemorrhage (Grade II or higher) or punctate white matter injury were excluded from the study. Additionally, 44 children (22 PT and 22 FT), recruited at Hanyang University Hospital, underwent standardized cognitive testing at a mean age of 4.45 years using the Wechsler Preschool and Primary Scale of Intelligence, Fourth Edition (WPPSI-IV), administered by trained examiners.

Of the 280 eligible participants, 18 were excluded from the morphometric analysis because of motion artefacts and poor image quality. A total of 262 participants were recruited for the morphometric analysis using suitable MRI data obtained at 1–7 years of postnatal age (PNA). An overview of cohort size, MRI eligibility, and outcome availability is summarized in Supplementary Fig. 1. The study protocol was prospectively approved by the Institutional Review Board of Hanyang University Hospital, and written informed consent was obtained from parents or legal guardians at the time of neonatal enrolment as part of the hospital’s neonatal follow-up programme. For the Jeonbuk National University Hospital cohorts, the Institutional Review Board granted retrospective approval for the use of de-identified clinical and imaging data, in accordance with the principles outlined in the Declaration of Helsinki.

### MRI acquisition

The present study was based on MRI data collected from Jeonbuk University Hospital, and Hanyang University Hospital was included to ensure alignment with the prospective neonatal MRI protocol. Because both hospitals participate in the Korean Neonatal Network, MRI acquisition was collaboratively standardized for research purposes, resulting in a unified single-vendor, single-protocol imaging framework. This approach maintained consistent imaging conditions across sites despite differences in data collection timing, thereby maximizing comparability between cohorts. Individual T1-weighted structural images were acquired using an MRI scanner (Philips Achieva 16-channel phase-array head coil; Best, The Netherlands) with a magnetization-prepared rapid gradient echo sequence. An experienced paediatrician monitored the pulse oximeter during the MRI to determine the heart and respiratory rates of each participant. The parameters for T1-weighted images were TE = 3.39 ms, TR = 2.10 ms, TI = 1 ms, field of view = 200 mm^2^, voxel sizes = 0.9 × 0.9 mm^2^, slice thickness = 1 mm and slice number = 150.

### Structural data processing

We processed the T1-weighted structural MRI images using FreeSurfer version 7.4.1 (https://surfer.nmr.mgh.harvard.edu/).^54^ The processing pipeline included bias field correction, motion and heterogeneity correction, transformation to the Talairach coordinate system, intensity normalization, skull stripping, white matter and grey matter tissue segmentation, white and pial surface reconstruction, and spherical mapping. Although there are relatively few studies focusing on children, FreeSurfer is still commonly used in this population.^55-57^ After the cortical surface reconstruction, we applied a spherically deformed surface registration with minimal distortion.^58^ The shape correspondence was then established using the registered spheres, and each sphere was resampled to the seventh level of the icosahedron subdivision (163 842 vertices).

Owing to the complexities involved in cortical surface parcellation in the developing brain, two independent researchers performed both automated and manual quality assessments of all reconstructed imaging data following several processes (Supplementary Text 1).

### Morphological feature extraction

Two key morphological features were extracted from the reconstructed cortical surfaces, SD^59^ and LGI^60^ (Supplementary Fig. 2). For SD and LGI, the cerebral hull surface, which represents the outer contour of the cortex, was used as a reference. SD was defined as the shortest trajectory from the cerebral hull to the cortical surface, obtained by solving a constant-speed Eikonal equation between two surfaces.^59^ Since SD is derived from a non-negative distance, its values are strictly positive and represent absolute geometric depth. LGI was computed using a shape-adaptive LGI introduced as a refinement of the conventional FreeSurfer’s LGI.^60^ A shape-adaptive LGI defines a spatially varying kernel that adapts to the local cortical folding. This is because the adaptive kernel is constructed through anisotropic wavefront propagation guided by a tensor field derived from cortical folding patterns. Moreover, the kernel size is globally rescaled according to the cortical surface area across different age groups to account for brain size variability. Together, these adaptations show higher reproducibility in the multi-scan dataset compared to FreeSurfer’s conventional LGI. We applied Gaussian spatial smoothing with a full width at half maximum of 6 mm to mitigate the impact of noise on SD.^61^

### Statistical analysis

#### Demographics

The demographics of the PT and FT infants were statistically compared using SPSS 27.0 (SPSS, Chicago, IL) software. We used the Mann–Whitney U-test and chi-square analysis to compare the clinical factors between the PT and FT groups.

#### Statistical models

Linear mixed-effects models were designed to investigate group differences between PT and FT infants in terms of cortical measurements (SD, LGI) during early childhood. Cortical measurements were used as dependent variables, and the fixed effects comprised two covariates: PNA and sex. Although the MRI scanners and acquisition protocols were identical at both sites, potential non-biological site-specific variations could confound subsequent analyses. To address this, we included hospitals as a random effect (random intercept) in the statistical models to absorb potential heterogeneity across sites and to estimate the true biological main and interaction effects more accurately. The following models were analysed:

#### Preterm and full-term difference model

We examined the overall effect of cortical measurements that differed between PT and FT infants while controlling for PNA and sex. We test the following linear mixed model:


(1) $$measure=\;\beta_{0}+\beta_{1}(PT/FT)+\beta_{2}PNA+\beta_{3}(PT/FT)*PNA+\beta_{4}sex+u_{0j}+\epsilon \;$$

where *β* represents fixed effects, (PT/FT) represents a binary variable indicating FT and PT, $u_{0j}$ denotes the hospital-specific random intercept that captures site-level variability and is assumed to follow $u_{0j}\;∼\;N(0,\;\sigma_{u}^{2})$, and ϵ represents random error.

##### GA subgroup differences model

We examined the overall effect of cortical measurements that differ between extremely-to-very PT (GA < 32 weeks; E-VP) and late PT (GA ≥ 32 weeks; LP) while controlling for PNA and sex. We test the following linear model:


(2) $$measure=\;\beta_{0}+\beta_{1}(E\_VP/LP)+\beta_{2}PNA+\beta_{3}(E\_VP/LP)*PNA+\beta_{4}sex+u_{0j}+\epsilon \;$$

where *β* represents fixed effects, (E_VP/LP) represents a binary variable indicating GA subgroup classification, $u_{0j}$ denotes the hospital-specific random intercept that captures site-level variability and is assumed to follow $u_{0j}\;∼\;N(0,\;\sigma_{u}^{2})$, and ϵ represents random error.

##### Validation of linear age modelling

As an additional validation analysis to assess the appropriateness of the linear age term, we evaluated higher-order age effects and compared nested models. The full procedures and results of this supplementary validation are provided in Supplementary Text 2.

##### Implementation details

We conducted statistical analysis using SurfStat^62^ a MATLAB toolbox that enables linear mixed-effects modelling,^63^ random field theory statistical correction, and visualization of cortical surfaces. The statistical analysis was conducted via cortical surface registration on a vertex-wise basis, without the need for cortical parcellation. Cortical surface parcellation was used only for visualization purposes by projecting the results onto a template surface. In this process, FreeSurfer’s default cortical atlas, the Desikan–Killiany–Tourville cortical labelling protocol with 31 labels was used.^64^ Statistical significance of parameters was corrected for multiple comparisons using random field theory^65^ at the level of 0.05 and cluster threshold (raw *P*-value) = 0.01.

### Group comparisons of structure-function associations

To evaluate whether the associations between cortical measures and neurodevelopmental outcomes differed between the PT and FT groups, we used linear regression models incorporating a group × cortical measure interaction term. For each cortical metric (SD and LGI) that showed significant group differences, separate models were constructed for the Verbal Comprehension Index (VCI), Visual Spatial Index (VSI), Fluid Reasoning Index (FRI), Working Memory Index and Full Scale IQ (FSIQ) scores from the WPPSI-IV. The general model specification was:


(3) $$outcome=\beta_{0}+\beta_{1}measure+\beta_{2}group+\beta_{3}measure*group+\beta_{4}PNA+\beta_{5}sex+\beta_{6}GA+\epsilon$$

where outcome represents VCI, VSI, FRI, WMI or FSIQ; measure refers to SD or LGI; and group is coded as PT versus FT. Analyses were adjusted for PNA and sex, and for analyses involving PT children, GA was additionally included as a covariate. The interaction coefficient (*β*_3_) was used to test whether the association between cortical measures and cognitive outcomes differed significantly between the groups. All *P*-values were corrected for multiple comparisons using the FDR procedure.

## Results

### Participant characteristics

A total of 262 children were included in the study, comprising 56 PT (mean age, 4.61 ± 1.57 years) and 206 FT (mean age, 4.36 ± 1.77) participants. The participants were grouped as follows: 8 participants aged 1 years, including 8 FT (mean age, 1.60 years); 38 participants aged 2 years, including 8 PT and 34 FT (mean age, 2.35 years); 50 at age 3 (15 PT; 35 FT; mean age, 3.43); 36 at age 4 (8 PT; 28 FT; mean age, 4.45); 33 at age 5 (8 PT; 25 FT; mean age, 5.51); 51 at age 6 (9 PT; 42 FT; mean age, 6.46); and 54 at age 7 (12 PT; 42 FT; mean age, 7.36) (Supplementary Fig. 3).

The mean GA was significantly lower in the PT group than in the FT group (31.43 ± 3.89 weeks versus 38.88 ± 1.80 weeks, *P* < 0.001). No significant differences in PNA were observed between groups (4.61 ± 1.57 years versus 4.36 ± 1.77 years, *P* = 0.438). The proportion of male participants did not significantly differ between the groups (71.4% versus 59.7%, *P* = 0.147). No significant differences were observed in maternal education levels between the PT and FT groups in any category (<12 years, *P* = 0.696; <16 years, *P* = 1.000; >16 years, *P* = 1.000). At follow-up, cognitive performance was assessed using the WPPSI-IV in a subgroup of participants (22 PT and 22 FT children). Compared with their FT peers, PT children demonstrated significantly lower scores in VCI (80.23 ± 24.24 versus 94.41 ± 12.93, *P* = 0.009), VSI (83.95 ± 20.08 versus 101.77 ± 16.18, *P* = 0.002), FRI (81.17 ± 21.15 versus 100.62 ± 17.30, *P* = 0.017), and WMI (82.91 ± 23.97 versus 99.65 ± 15.03, *P* = 0.011), and FSIQ was significantly lower in the PT group (76.45 ± 23.32 versus 98.14 ± 15.39, *P* < 0.001). Processing Speed Index (PSI) did not show a statistically significant difference between the groups (80.27 ± 22.73 versus 87.91 ± 18.81, *P* = 0.401). Supplementary Table 1 shows the characteristics of the participants, including perinatal factors and neurodevelopmental outcomes.

### Interpretations of LGI and SD

**Figure 1:**
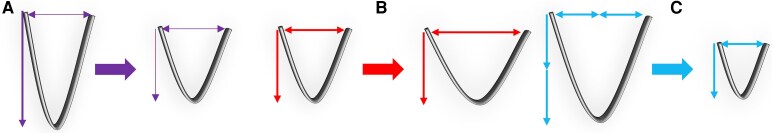
**Influence of sulcal width and depth on LGI.** Variations in LGI can result from changes in sulcal depth, width, or their combination. To analyse different scenarios, consider three characteristics: (**A**) half depth (both LGI and SD decreases); (**B**) double width (only LGI decreases); (**C**) half width and depth (only SD decreases, and LGI does not necessarily decrease). Thus, sulcal depth and LGI need to be considered together to explicitly explain cortical folding differences.

We performed a combined analysis of LGI and SD to capture these distinct aspects of cortical morphology, as LGI alone cannot distinguish between sulcal depth and width changes.^38^ Based on the present results, we noted three characteristic region-specific folding alterations: concurrent reductions in LGI and SD, reduced LGI with preserved SD, and decreased SD while maintaining the width-to-depth ratio (Fig. 1).

### Preterm and full-term group differences analysis

**Figure 2:**
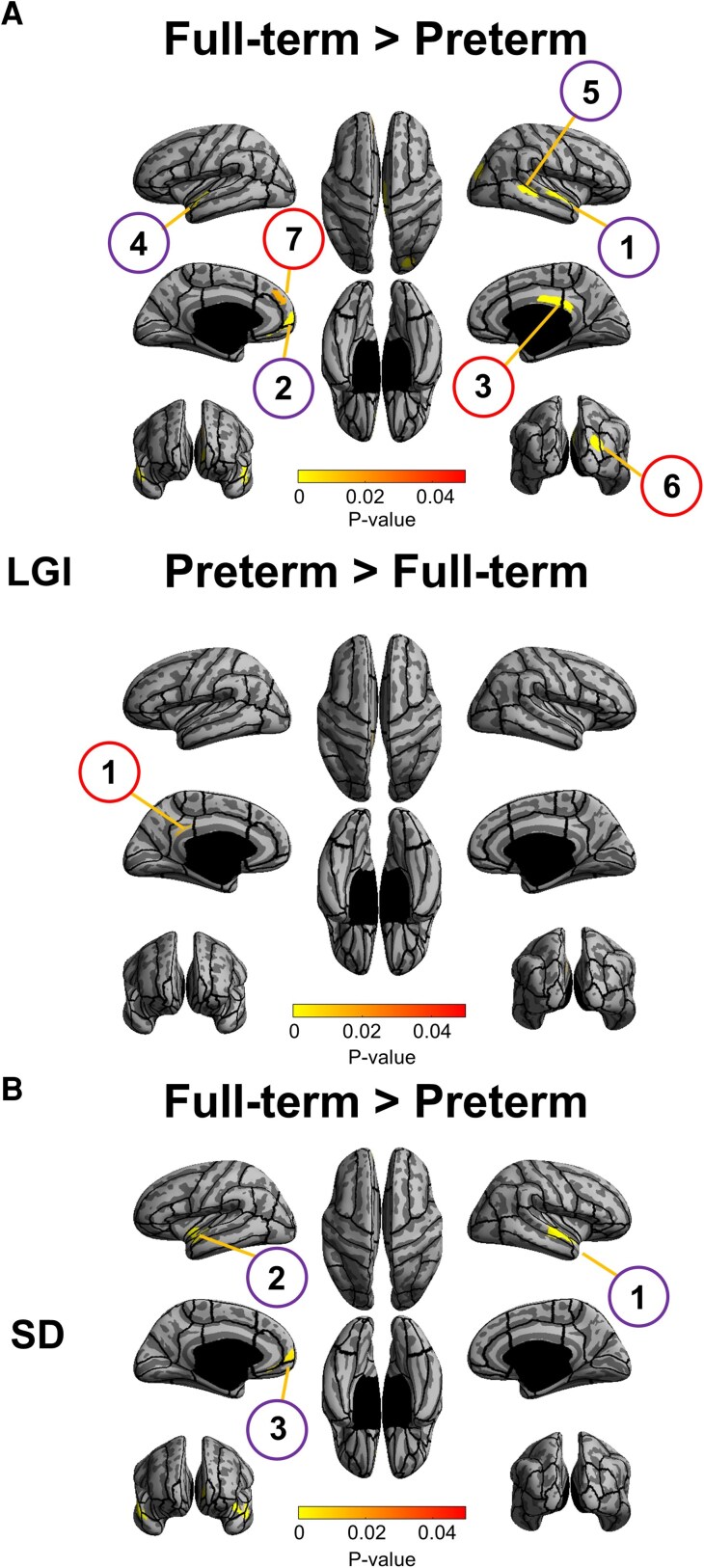
**Preterm and full-term group differences in cortical measurements.** Regions of statistically significant group differences in LGI (**A**) and SD (**B**) are shown, coloured according to cluster corrected *P*-value (bottom scale). First and third row indicate regions of lesser LGI and SD in the preterm group than in the full-term group, and vice versa in the second row, at the *P* < 0.05 level after correcting for multiple comparisons via random field theory. Group differences were assessed using a linear mixed model adjusted for PNA and sex. Clusters were highlighted with a purple circle when both LGI and SD are significant and highlighted with a red circle when only LGI is significant. The analysis included 56 preterm and 206 full-term children.

Statistical analysis revealed significant differences in cortical measurements between PT and FT infants in different regions, as shown in Figure 2.

The FT group exhibited significantly higher LGI in seven clusters: the right superior temporal gyrus (STG; anterior and posterior parts), left STG, left superior frontal gyrus (SFG; anterior and middle parts), right posterior cingulate (PCG), isthmus cingulate gyrus (ICG), and right lateral occipital region. SD was significantly higher in the three clusters localized to both the STG and left SFG regions (Table 1). The LGI was significantly higher in the PT group in one cluster and was localized to the left ICG. No significant differences were observed in SD in this contrast.

**Table 1:** Group differences in cortical measurements

Index	Cluster	Region	Adj. *P*-value
FT > PT			
LGI	1	Right superior temporal (anterior part)	0.0001<
	2	Left superior frontal (anterior part)	0.0001<
	3	Right posterior cingulate, isthmus cingulate	0.0002
	4	Left superior temporal	0.0003
	5	Right superior temporal (posterior part)	0.0010
	6	Right lateral occipital	0.0013
	7	Left superior frontal sulcus (middle part)	0.0156
SD	1	Right superior temporal (anterior part)	0.0001<
	2	Left superior temporal	0.0001<
	3	Left superior frontal (anterior part)	0.0039
**PT** **>** **FT**			
LGI	1	Left isthmus cingulate	0.0013

Cortical measurement index, cluster number per contrast (in ascending order of correct *P*-value), regions of cluster localization, and corrected *P*-values. For group differences, the direction of contrast FT > PT indicates that cortical measurement in the full-term infants is greater than that in the preterm infants and vice versa.

**Figure 3:**
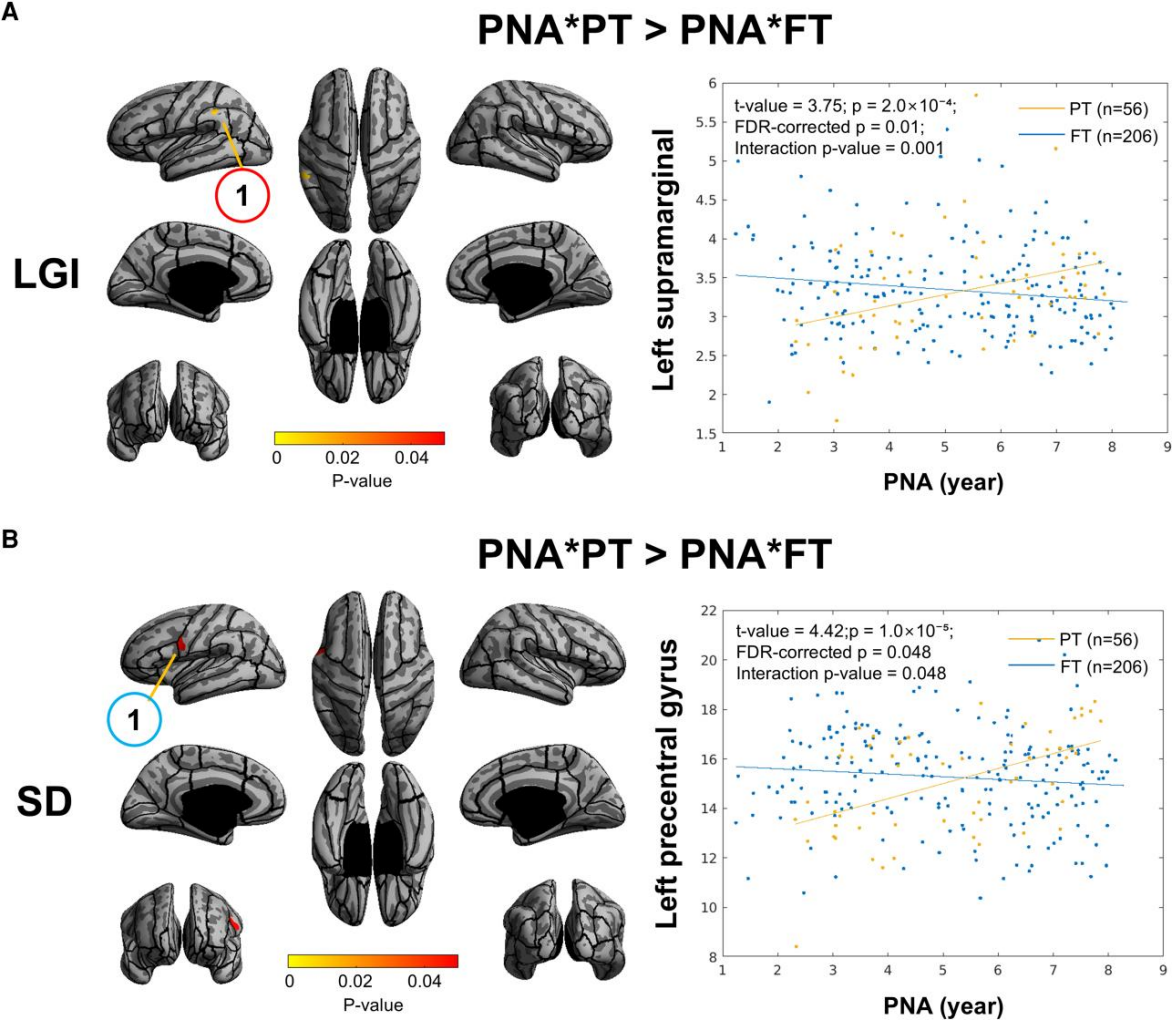
**Statistically significant regions of PNA by group interactions by cortical measurements.** Regions of statistically significant PNA by group differences in LGI (**A**) and SD (**B**) are shown, coloured according to cluster *P*-value (Bottom scale). First row indicates regions of lesser slope with PNA in the preterm group than in the full-term group and vice versa in the remaining two rows, at the *P* < 0.05 level after correcting for multiple comparisons via random field theory. PNA by group interactions were tested using a linear mixed model adjusted for sex. Clusters were highlighted with a red circle when only LGI is significant and highlighted with a blue circle when only SD is significant. The scatter plot displays individual data points with fitted regression lines for each group, and the corresponding statistical test results (t-value, group sizes, *P*-value, FDR-corrected *P*-value, and interaction *P*-value) are reported within the figure. Each data point represents an individual child (PT, yellow; FT, blue), with a total of 56 preterm and 206 full-term children included in the analysis.

Scatterplots for all the identified clusters are shown in Supplementary Figure 4A and B. Most regions showed a lower LGI and/or SD in the PT group from 1 to 7 years of age. However, LGI in the PT group showed a significantly greater increase in PNA in the left supramarginal region. SD showed a significantly greater increase in PNA than the PT group in one cluster localized to the left precentral gyrus (PreCG) (Fig. 3).

### E-VP and LP group differences analysis in preterm children

**Figure 4:**
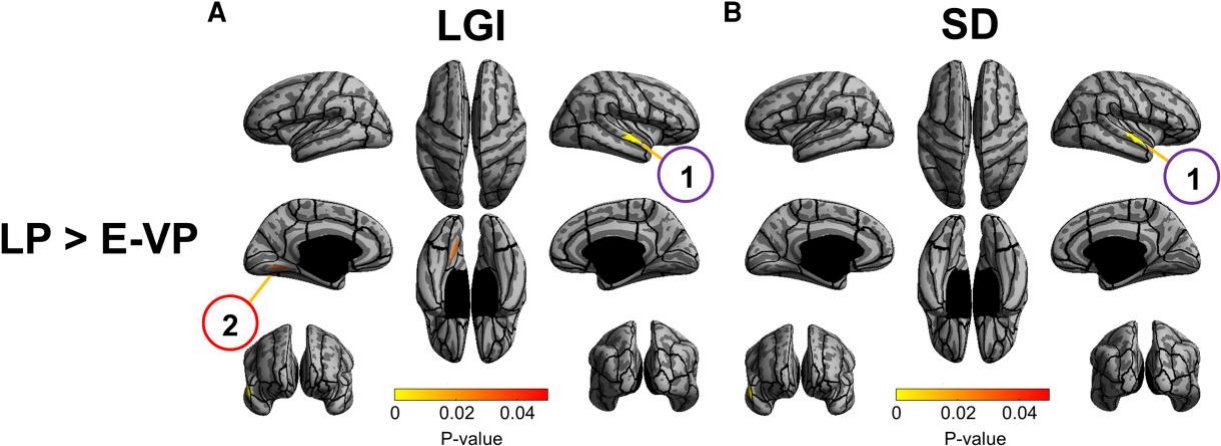
**Preterm subgroup differences in cortical measurements.** Regions of statistically significant group differences in LGI (**A**) and SD (**B**) are shown, coloured according to cluster corrected *P*-value (bottom scale). The results indicate regions of lesser LGI and SD in the E-VP group than in the LP group, at the *P* < 0.05 level after correcting for multiple comparisons via random field theory. Group differences were assessed using a linear mixed model adjusted for postnatal age and sex. Clusters were highlighted with a purple circle when both LGI and SD are significant and highlighted with a red circle when only LGI is significant. The analysis included 28 E-VP and 28 LP children.

Statistical analysis revealed significant differences in the cortical measurements between the E-VP and LP groups at different regions, as shown in Fig. 4. Scatterplots illustrating all identified clusters are presented in Supplementary Figure 4C and D.

*LGI* was significantly lower in the E-VP group in two clusters localized to the right STG and left LING. SD was significantly lower in one cluster located in the right STG. No statistically significant clusters were observed for LGI or SD in the E-VP > LP contrast (Table 2).

**Table 2:** Differences in cortical measurements in the preterm subgroup

Index	Cluster	Region	Adj. *P*-value
**LP** **>** **E-VP**			
LGI	1	Right superior temporal (anterior part)	0.0001<
	2	Left lingual	0.0279
SD	1	Right superior temporal (anterior part)	0.0004

Cortical measurement index, cluster number per contrast (in ascending order of corrected *P*-value), regions of cluster localization, and corrected *P*-values. For subgroup differences, the direction of contrast LP > E-VP indicates that the cortical measurement in the LP is greater than that in the E-VP, and vice versa.

**Figure 5:**
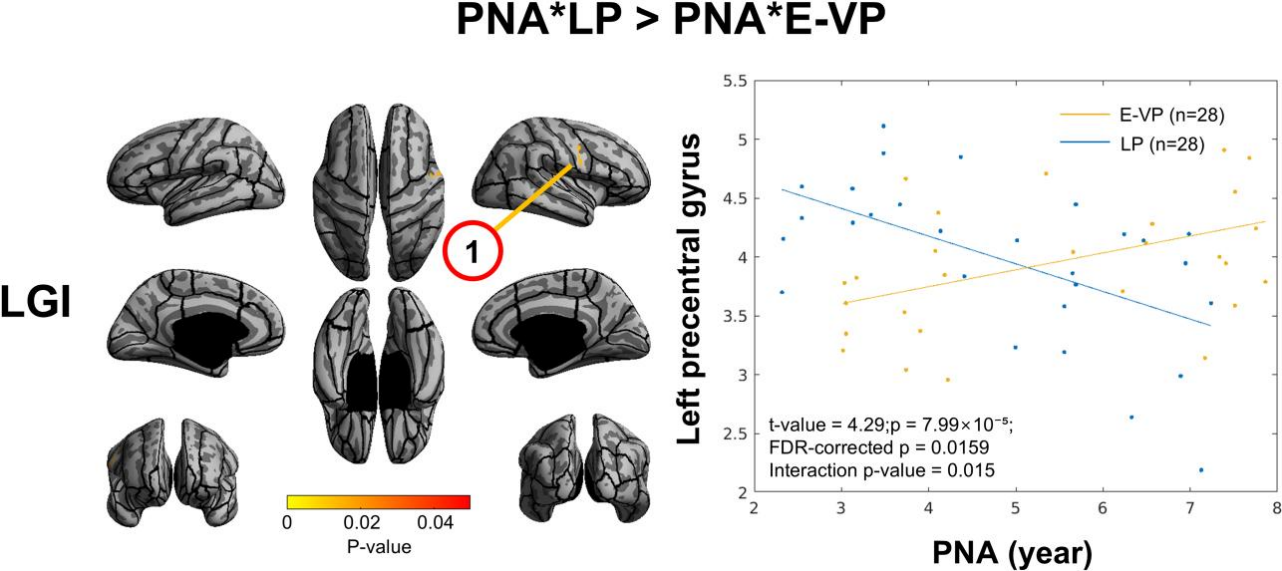
**Statistically significant regions of PNA by preterm subgroup interactions by cortical measurements.** Regions of statistically significant PNA by group differences in LGI. The results indicate regions where the slope with PNA is less in the E-VP group than in the LP group for LGI, at the *P* < 0.05 level after correcting for multiple comparisons via random field theory. PNA by preterm subgroup interactions were tested using a linear mixed model adjusted for sex. The cluster was highlighted with a red circle when only LGI is significant. The scatter plot displays individual data points with fitted regression lines for each group, and the corresponding statistical test results (t-value, group sizes, *P*-value, FDR-corrected *P*-value, and interaction *P*-value) are reported within the figure. Each data point represents an individual child (E-VP, yellow; LP, blue), with 28 children included in each preterm subgroup.

When examining PNA-related subgroup differences, only one statistically significant cluster was found for LGI, where the E-VP group showed a significantly greater increase in PNA compared to the E-VP group in the right PreCG. No significant interaction effects were observed for SD (Fig. 5).

### Group differences in associations between cortical measurements and neurodevelopmental outcomes

Statistical analysis revealed group differences in the associations between cortical measures and neurodevelopmental outcomes based on linear models incorporating group × cortical measure interaction terms (Table 3). In the right superior temporal region, the interaction between SD and VCI was significant (β = −18.48, FDR *P* = 0.014), whereas the interaction for LGI did not reach statistical significance (β = −11.53, FDR *P* = 0.822). For WMI, LGI showed a nominal interaction effect (β = −32.47, FDR *P* = 0.129), while SD demonstrated a significant interaction (β = −20.62, FDR *P* = 0.014). For FSIQ, the interaction effect was not significant for LGI (β = −19.72, FDR *P* = 0.543), but SD showed a significant interaction (β = −17.65, FDR *P* = 0.031). In the left superior temporal region, LGI demonstrated a nominal interaction effect with WMI (β = −19.95, FDR *P* = 0.129), whereas SD exhibited a significant interaction (β = −23.51, FDR *P* = 0.004). Full results are provided in Supplementary Tables 2 and 3.

**Table 3:** Group differences in the associations between cortical measures and WPPSI-IV subsets

Region	WPPSI Subset	Index	β (group × measures)	*P*-value	FDR *P*-value
Right superior temporal	VCI	LGI	−11.53	0.352	0.822
		SD	−18.48	0.005*	0.014*
	WMI	LGI	−32.47	0.032*	0.129
		SD	−20.62	0.009*	0.014*
	FSIQ	LGI	−19.72	0.126	0.543
		SD	−17.65	0.010*	0.031*
Left superior temporal	WMI	LGI	−19.95	0.037*	0.129
		SD	−23.51	0.001*	0.004*

Full results are provided in Supplementary Table S2 and S3.

^*^ *P* < 0.05.

## Discussion

In this study, we applied a cortical surface registration technique to establish intersubject anatomical correspondence and minimize registration-induced distortions. This anatomically precise alignment enabled robust group-wise comparisons of cortical morphometry in early childhood, revealing regionally specific deviations in cortical folding among PT children. Compared with their FT counterparts, PT infants exhibited significantly reduced LGI and SD in the bilateral STG and left SFG, suggesting a region-specific disruption of perinatal cortical morphogenesis. In the right ICG and PCG, the reduced LGI suggested incomplete gyral expansion during secondary and tertiary folding in early infancy. These alterations persisted from the ages of 1–7 years, indicating enduring deviations from normative folding trajectories.

Compared to the FT group, the PT group exhibited lower LGI and SD in both the STG and left SFG, reflecting altered gyral and sulcal maturation that could potentially restrict experience-dependent plasticity in higher-order cognitive regions. The STG and SFG emerge at approximately 20 weeks GA and undergo rapid morphological changes at approximately 24 weeks.^66^ Previous studies using surface-based analyses of cortical gyrification in healthy PT and FT infants have shown that folding progression is particularly marked within temporal and frontal association cortices across the perinatal period,^11,67^ and this regionally elevated growth trajectory appears to extend into the first two years after birth.^15^ This developmental profile aligns with a hierarchical maturation process in which association cortices gradually mature over an extended period through synaptic pruning, progressive myelination, and the establishment of long-range associative connections.^25,68,69^ Moreover, the expansion of axonal and synaptic architecture imposes increasing tangential growth demands on the cortical sheet, which can manifest as gyrification patterns that facilitate the efficient spatial embedding of distributed networks.^70^ Together, these folding mechanisms form a structural basis that enables experience-dependent refinement after birth and supports the emergence of higher-order cognitive functions. Interestingly, Hill *et al.*^71^ proposed that cortical regions related to higher-order cognitive processing maintain comparatively low levels of brain morphological maturity during the foetal period to preserve a window for postnatal experience-dependent neuroplasticity. Ronan *et al.*^72^ further reported that these regions undergo spatially heterogeneous tangential expansion after birth, suggesting that such variability may be a typical developmental feature supporting experience-dependent refinement. Given that gyrification is informative in capturing structural variability across cortical regions,^73-75^ reductions in LGI and SD observed in the STG and SFG suggest that PT birth may be associated with delayed or incomplete structural maturation, potentially limiting the engagement of neuroplastic mechanisms in regions subserving higher-order cognitive functions. Our suggestions are supported by previous studies. Ball *et al.*^25^ suggested that cortical alterations in the STG and SFG observed in PT infants are associated with genes regulating early maturing inhibitory neurons and that this may fail to follow the hierarchical maturation map after PT birth. From a macroscopic perspective, Engelhardt *et al.*^76^ reported that PT infants exhibit reduced surface area and lower GIs compared to FT infants, particularly in the STG and its adjacent regions.

Moreover, Papini *et al.*^77^ found reduced LGI in the STG and SFG among adults born very PT and suggested that early alterations in neural substrates due to PT birth may lead to qualitative differences in the relationship between cortical folding and adult mental health outcomes. Similarly, we found that the association between SD and VCI, WMI, and FSIQ was markedly reduced in the PT children (with a similar pattern for WMI in the left STG). These results highlight that PT birth not only alters folding morphology but also that the atypical folding patterns may be associated with early extrauterine exposure, which can fail to confer, and may even diminish, the neurodevelopmental advantages observed during normative development. Given that the STG functions as a hub for higher-order auditory associations, aberrant folding in this region could perturb auditory–language pathways and, in turn, manifest as differences in language, verbal working memory and general intellectual ability.^78-80^

The right STG, identified as differing in both LGI and SD between the PT and FT groups, was significantly reduced in the E-VP group in the PT subgroup analysis, extending the influence of GA on cortical development. Extrauterine exposure in infants with E-VP overlaps with a critical period of early cortical folding, during which cumulative stress may exert long-lasting effects on the neurobiological substrates of gyrification. Collectively, these findings demonstrate that disrupted gyral maturation limits experience-dependent plasticity normally engaged in the temporal association cortices, thereby contributing to altered neurodevelopmental trajectories in PT infants.

The right ICG and PCG in PT children displayed a pattern of reduced LGI with preserved SD, characterized by relatively wide but not shallow sulci, indicating simplified higher-order folding due to the disrupted elaboration of secondary and tertiary cortical folds. These atypical cortical folding patterns may be interpreted within the context of the following mechanistic frameworks. First, the differential growth hypothesis posits that asynchronous expansion between cortical layers and adjacent regions leads to folding.^81^ PT birth may disrupt this equilibrium, limiting the mechanical tension and geometric conditions required for complex cortical folding, particularly the secondary and tertiary folds that typically develop during late gestation. Recent developmental evidence suggests that PT infants fail to follow the typical trajectory of asynchronous cortical expansion associated with the migration of subplate neurons during the formation of complex secondary and tertiary folds.^11^ Given that the cingulate sulcus continues to stabilize structurally beyond the age of seven,^82^ early disturbances may result in long-lasting deviations from normative morphogenesis.^19^ Second, according to the tension-based theory, the folding pattern is shaped by mechanical tension generated through long-distance axonal connectivity,^12^ and premature birth may weaken these forces owing to reduced myelination and impaired axonal integrity.^83^ Supporting this view, previous neuroimaging studies have reported that the cingulate sulcus in very PT children is shorter and more fragmented than that in their FT peers, suggesting incomplete development of long-range connectivity.^84^ Moreover, the cingulum bundle plays a crucial role in integrating emotional and attentional processing by connecting the medial prefrontal, parietal, and temporal regions, thereby facilitating communication across the networks involved in self-referential thinking, memory retrieval, executive functioning, and emotional regulation.^85-87^ Disruption of the integrity or morphogenesis of the cingulate cortex and its associated white matter pathways may compromise a wide range of socioemotional outcomes observed in children born PT.^83^ This converging evidence suggests the potential of ICG and PCG as biomarkers for long-term psychosocial outcomes in PT infants.

PNA-related cortical maturation of the left PreCG (in SD) showed divergent intergroup developmental patterns during the perinatal period but converged around 5–6 years of age. These regions, the primary visual and motor cortices, are characterized by early structural maturation and low inter-individual variability, as shown in previous studies,^4,7,15,87^ and are therefore considered structurally stable during early cortical development. Nevertheless, divergent patterns of early cortical development may reflect altered cortical maturation induced by extrauterine exposure, including disrupted sensory-driven cortical morphogenesis or delayed thalamocortical connectivity in the sensorimotor cortex, which may ultimately impair cortical folding and the efficiency of neural communication through white matter pathways.^88,89^ Consequently, the subsequent convergence of developmental trajectories may reflect experience-dependent neuroplasticity and delayed alignment with normative cortical growth patterns in PT infants. Notably, strong thalamocortical inputs and preserved structural scaffolding may have contributed to the catch-up maturation.^90^ These findings align with those of previous reports indicating compensatory development in sensorimotor hubs^91-93^ and suggest that such regions may provide a foundation for the emergence of higher-order cognitive functions.^33,35,94,95^

Technically, traditional methods for computing LGI^54^ capture the overall cortical folding patterns reasonably well, but they are sometimes unable to reflect the finer details of local cortical morphology, as they do not explicitly incorporate folding patterns into the computation.^60^ To address this limitation, we employed a shape-adaptive LGI^60^ method that better reflected the local folding patterns of the cortex. This method has demonstrated improved sensitivity to region-specific developmental changes, particularly those not identified by traditional approaches.^38,96^ It is also well-suited for paediatric populations that exhibit substantial inter-individual variability.

Despite the strengths of this study, it has some clinical limitations that must be acknowledged. First, the cross-sectional design limited our ability to directly infer longitudinal developmental trajectories or track intra-individual changes in cortical morphology over time. As such, although we observed age-related patterns and group differences, these findings cannot definitively establish the causality or temporal dynamics of folding development. Second, the PT sample size was not sufficiently large to capture the broad developmental window (1–7 years) during which cortical maturation progresses rapidly, which may reduce the precision of age-related inferences. These results should be interpreted cautiously and validated in larger cohorts. Third, only a subset of children completed cognitive assessments, limiting the robustness of neurodevelopmental associations. These results should be viewed as preliminary and require replication with broader behavioural sampling. Fourth, our analysis was based solely on T1-weighted imaging and did not include concurrent diffusion tension imaging or other white matter-sensitive modalities. As a result, we were limited in our ability to interpret the observed cortical folding changes in the context of white matter integrity or long-range axonal connectivity,^90,97^ both of which are thought to play mechanistic roles in cortical morphogenesis.^36,98,99^ Multimodal approaches that integrate structural, diffusion, and functional imaging would offer a more comprehensive understanding of the neurodevelopmental consequences of premature birth.

One methodological limitation concerns the choice of the cluster threshold in multiple comparison correction. In this study, we applied a relatively liberal threshold of *P* < 0.01, which differs from the conventional standard in surface-based neuroimaging (*P* < 0.001). This decision was made to account for the limited sample size of the PT group, which substantially reduced statistical power and made it difficult to detect significant clusters after multiple comparison correction. We acknowledge that this approach increases the potential risk of false positives. To address this issue, we additionally provide results based on a stricter threshold of *P* < 0.001 in the Supplementary Materials and discuss the discrepancies between the two results as an important limitation (Supplementary Figs 5–8). Future studies with larger sample sizes are expected to resolve this limitation.

Overall, our findings underscore the fact that PT birth induces region-specific disruptions in cortical folding that persist into early childhood and influence structure–function relationships. These alterations exhibit vulnerability that becomes more evident along the functional hierarchy from the primary sensorimotor to the higher-order associative cortices. While certain primary sensorimotor areas, such as the preCG and supramarginal gyrus, exhibit signs of structural recovery, regions implicated in higher-order cognitive processing, including the SFG, STG, and PCG, appear to be persistently altered. The use of folding-sensitive markers such as LGI and SD provides critical insights into the long-term neurodevelopmental consequences of prematurity and supports the design of regionally targeted interventions.

## Supplementary Material

fcag097_Supplementary_Data

## Data Availability

The cohort datasets generated and/or analysed during the current study are not publicly available because of the inability to share personal information according to research ethics but are available from the corresponding author upon reasonable request. Correspondence and requests for materials should be addressed to YHJ (ryanjang93@hanyang.ac.kr) and HJL (blesslee77@hanmail.net). All codes and computational tools used in this study are publicly accessible. The shape-adaptive local gyrification index algorithm is available at: https://github.com/ilwoolyu/LocalGyrificationIndex. The hierarchical spherical deformation framework for cortical surface registration is available at: https://github.com/ilwoolyu/HSD. Statistical analyses were performed using SurfStat (https://www.math.mcgill.ca/keith/surfstat/). A Docker image that integrates cortical morphometry tools, including local gyrification index, sulcal depth and hierarchical spherical deformation, is available at: https://hub.docker.com/r/ilwoolyu/cmorph. These code resources are also provided in the Supplementary Materials.
