# Correction to: Long-term effects of preterm birth on cortical folding trajectories in early childhood

**DOI:** 10.1093/braincomms/fcag314

**Published:** 2026-08-18

This is a correction to: Yong Hun Jang, Jong Min Kim, Bong Gun Lee, Jeong-Kyu Hoh, Gang Yi Lee, Hyun Ho Kim, Ilwoo Lyu, Hyun Ju Lee, Long-term effects of preterm birth on cortical folding trajectories in early childhood, *Brain Communications*, Volume 8, Issue 3, 2026, fcag097, https://doi.org/10.1093/braincomms/fcag097

In the originally published version of this manuscript, MSIT grant numbers RS-2024-00333931 and RS-2025-02216257 were missing from the funding statement.

This error has been corrected.

