# Supplementary material for: Long-term effects of preterm birth on cortical folding trajectories in early childhood
Source: Brain Commun. 2026 May 18;8(3):fcag097. doi: 10.1093/braincomms/fcag097 (PMC13181400; doi:10.1093/braincomms/fcag097)
Supplement: fcag097_Supplementary_Data [file fcag097_supplementary_data.zip › Revision 1.pdf]

OXFORD UNIVERSITY PRESS | Brain Communications

Long-term effects of preterm birth on cortical folding trajectories in early childhood

Journal:	*Brain Communications*
Manuscript ID	BRAINCOM-2025-821.R1
Manuscript Type:	Original Article
Date Submitted by the Author:	25-Dec-2025
Complete List of Authors:	Jang, Yong Hun; Hanyang University College of Medicine, Department of Pediatrics<br>Kim, Jong Min; POSTECH, Graduate School of Artificial Intelligence<br>Lee, Bong Gun; Hanyang University College of Medicine, Department of Orthopaedic Surgery<br>Hoh, Jeong-Kyu; Hanyang University College of Medicine, Department of Obstetrics and Gynaecology<br>Lee, Gang Yi; Hanyang University Graduate School of Biomedical Science and Engineering, Translational Medicine<br>Kim, Hyun Ho; Jeonbuk National University College of Medicine, Department of Paediatrics<br>Lyu, Ilwoo; POSTECH, Graduate School of Artificial Intelligence; POSTECH, Department of Computer Science and Engineering<br>Lee, Hyun Ju; Hanyang University College of Medicine, Department of Paediatrics; Hanyang University, Hanyang Institute of Bioscience and Biotechnology
Keywords:	Preterm Infants, Early Childhood, Cortical Folding, Local Gyrification Index, Sulcal Depth

SCHOLARONE™
Manuscripts

Long-term effects of preterm birth on cortical folding trajectories in early childhood

Yong Hun Jang[1†], Jong Min Kim[2†], Bong Gun Lee[3], Jeong-Kyu Hoh[4], Gang Yi Lee[5], Hyun Ho Kim[6], Ilwoo Lyu[2, 7*] and Hyun Ju Lee[1, 8*]

[†]These authors contributed equally to this work.

For Review Only

Abstract

Cortical folding emerges in the late prenatal period and undergoes rapid reorganization during early childhood. However, the long-term impact of folding alterations associated with preterm birth remains unclear. Herein, we analyzed the structural MRI data of 56 preterm children and 206 full-term peers aged 1–7 years. We derived cortical metrics from the reconstructed cortical surfaces using a vertex-wise computation framework to characterize regional folding patterns. We then conducted a combined analysis of the local gyrification index and sulcal depth to explain folding patterns in the preterm brain. Compared with their full-term peers, preterm children exhibited a region-specific impairment pattern characterized by a significantly reduced local gyrification index and sulcal depth in the bilateral superior temporal gyrus and left superior frontal gyrus ($P < 0.05$). Notably, sulcal depth in the superior temporal cortex showed significant differences between preterm and full-term children in its association with neurodevelopmental outcomes ($P < 0.05$), indicating an atypical structure–function relationship in preterm children. The local gyrification index was significantly reduced in the right isthmus cingulate and posterior cingulate gyri ($P < 0.05$), reflecting a simplified gyral configuration. The study findings suggest several folding patterns that capture diverse mechanisms of morphogenetic disruption, indicating that preterm birth induces persistent

region-specific impairments in cortical folding that may affect neurodevelopmental domains. These folding-sensitive markers provide critical insights into the development of targeted interventions to optimize long-term neurodevelopmental outcomes.

Author affiliations:

[1]Department of Paediatrics, Hanyang University Hospital, Hanyang University College of Medicine, Seoul, 04763, Republic of Korea

[2]Graduate School of Artificial Intelligence, POSTECH, Pohang 37673, South Korea

[3]Department of Orthopaedic Surgery, Hanyang University Hospital, Hanyang University College of Medicine, Seoul, 04763, Republic of Korea

[4]Department of Obstetrics and Gynaecology, Hanyang University Hospital, Hanyang University College of Medicine, Seoul, 04763, Republic of Korea

[5]Department of Translational Medicine, Hanyang University Graduate School of Biomedical Science and Engineering, Seoul 04763, Republic of Korea

[6]Department of Paediatrics, Jeonbuk National University School of Medicine, Jeonju, 54896, Republic of Korea

[7]Department of Computer Science and Engineering, POSTECH, Pohang, 37673, South Korea

[8]Hanyang Institute of Bioscience and Biotechnology, Hanyang University, Seoul, 04763, Republic of Korea

Correspondence to:

Ilwoo Lyu, PhD

Department of Computer Science and Engineering, POSTECH, Pohang, 37673, South Korea

Graduate School of Artificial Intelligence, POSTECH, Pohang, 37673, South Korea

ilwoolyu@postech.ac.kr

For Review Only

Hyun Ju Lee, MD, PhD
Department of Paediatrics, Hanyang University Seoul Hospital, 222-1, Wangsimni-ro, Seongdong-gu, Seoul, 04763, Republic of Korea
blesslee77@hanmail.net

Running title: Preterm Birth and Early Cortical Folding

Keywords: Preterm infants; Early childhood; Cortical folding; Local gyrification index; Sulcal depth

For Review Only

Introduction

Cortical folding in the human brain occurs during the third trimester of gestation and continues throughout infancy and early childhood. Dynamic and progressive folding processes are orchestrated by a complex interaction of genetic programs, mechanical forces, and environmental inputs, facilitating initial cognitive development.[1,2] Despite notable inter-individual variability, sulcal emergence follows a highly stereotyped gene-driven spatiotemporal sequence that is conserved across individuals.[3-10] During the third trimester, primary sulci such as the precentral, calcarine, and cingulate sulci emerge rapidly, followed by secondary and tertiary folding at approximately 32 and 38 weeks postmenstrual age, respectively.[11] Leading theoretical models have proposed that cortical folding is either induced by axonal tension along long-range white matter tracts[12] or continuously modified by local mechanical instability arising from region-specific heterogeneous growth in the outer cortical layers.[13,14] Longitudinal cohort studies have shown that the global gyrification index (GI) increases by approximately 23.7% between term-equivalent age and age two in typically developing children,[15] with annual increases in GI falling below 0.5% after age six, suggesting that gyrification may reach its peak before age six.[16-18]

The emergence of major folding coincides with the period of preterm birth, raising concerns about the vulnerability of cortical folding processes to extrauterine environmental perturbations. At term-equivalent age, preterm infants exhibit fewer complex patterns of secondary and tertiary folding than do full-term infants, indicating an increased vulnerability of cortical folding to environmental morphogenetic factors during this sensitive period.[11] Early disruption of cortical folding may impair the structural coupling between cortical folding complexity and long-range white matter connectivity, thereby altering subsequent regional specialization during neurodevelopment.[19,20] Such deviations from the normative folding

For Review Only

trajectories have been consistently linked to long-term cortical dysmaturation and cognitive impairments.[21-24]

MRI studies comparing preterm and full-term individuals have consistently revealed alterations in the spatial patterns of gene expression[25] and cortical microstructures that persist from term-equivalent age through childhood.[26-29] However, elucidating the specific effects of extrauterine exposure on regional brain development remains challenging due to the multifactorial interplay between genetic, epigenetic, and environmental factors. Recent longitudinal studies examining cortical development in preterm versus full-term populations have typically relied on macrostructural metrics, such as surface area, cortical thickness, and volume.[28,30-35] While these metrics offer valuable insights into group-level anatomical differences and general developmental trends, they are limited in their capacity to comprehensively capture the region-specific variability of early cortical development, morphological complexity of long-term cortical maturation, and associated pathological risk factors. Furthermore, existing theoretical frameworks on cortical folding have predominantly emphasized the early postnatal period (0–2 years of age), with comparatively less attention paid to the subsequent phase of accelerated brain reorganization that occurs between 2 and 7 years of age.[15,36] Given that ongoing brain structural changes and the emergence of various cognitive functions occur from 1 to 7 years of age,[37] the present study aimed to clarify the spatiotemporal differences between preterm and full-term folding patterns.

In light of this need, the present study investigated cortical maturation from 1 to 7 years of age following preterm birth within a biomechanical framework, focusing on two shape-sensitive markers of cortical folding: the local gyrification index (LGI) and sulcal depth (SD). The combination of LGI and SD has emerged as a clinically interpretable measure that offers sensitivity to developmental and pathological variations that may not be reflected in

For Review Only

conventional metrics.[38] From a macro perspective, LGI reflects the proportion of the buried cortex within a given region, capturing local folding complexity,[39] while SD quantifies sulcal invagination to detect subtle morphological changes.[9] Specifically, these morphological indices are particularly responsive to disruptions in early neurodevelopmental processes, including neuronal migration, cortical lamination, and white matter expansion,[40] and later refinements, such as dendritic arborization and thalamocortical innervation.[41-43] These measures reflect cortical complexity that may be associated with differential function, given that cortical folding patterns are influenced by the underlying cytoarchitecture and neural connections. Furthermore, LGI and SD are highly sensitive to structural alterations in regions influenced by postnatal environmental factors and white matter expansion[15,44] and have validated the utility of mapping regionally specific maturation patterns across early developmental windows.[45-49] Moreover, aberrant patterns of cortical folding have been increasingly reported in individuals with psychiatric disorders such as schizophrenia,[50] bipolar disorder,[51] depression,[52] and anxiety.[53] These findings suggest that deviations in early sulcal formation, driven by both genetic programming and environmental perturbations during fetal and infant neurodevelopment, may induce mechanical disequilibrium in cortical morphogenesis, thereby increasing vulnerability to later psychiatric disorders. Taken together, these findings underscore the relevance of folding-based morphometrics as biomarkers of atypical neurodevelopmental trajectories.

This study was based on the hypothesis that children born preterm would exhibit altered trajectories of cortical folding between the ages of 1 and 7 years due to preterm birth-related disruptions in the cortical architecture. To examine this, we evaluated a cross-sectional cohort comprising 56 preterm and 206 full-term children by extracting LGI and SD, along with assessing their neurodevelopmental outcomes. Technically, we utilized a shape-adaptive LGI

For Review Only

measure to increase the sensitivity to microstructural alterations during periods of accelerated cortical reorganization and interpreted it in conjunction with SD. Furthermore, we identified the associations between cortical metrics and neurodevelopmental outcomes to provide clinically meaningful insights.

For Review Only

Materials and methods

Study populations

The present study included preterm and full-term participants aged 1–7 years who were recruited from two independent hospital cohorts for a cross-sectional analysis. A total of 37 preterm infants born at <37 weeks' gestational age (GA) was admitted to the neonatal intensive care unit (NICU) of Hanyang University Hospital and prospectively enrolled in a follow-up project at the Hanyang Inclusive Clinic for Developmental Disorders between 2017 and 2022. Another cohort of 26 preterm infants was retrospectively recruited from the NICU of the Jeonbuk National University Hospital between 2017 and 2021. For the full-term group, 86 infants born at ≥37 weeks of GA were prospectively recruited within 1 week of birth from the newborn nursery at Hanyang University Hospital, and 131 typically developing children were retrospectively recruited from Jeonbuk National University Hospital between 2017 and 2024. The cohorts from both institutions were recruited at tertiary care centers within South Korea's National Health Insurance system, and they shared similar socioeconomic conditions in access to neonatal and long-term developmental services. Preterm infants with known severe bronchopulmonary dysplasia, congenital brain abnormalities, congenital infections, cystic periventricular leukomalacia, diffuse ventriculomegaly, genetic disorders (clinically or radiologically suspected), focal brain lesions, intraventricular hemorrhage (grade II or higher), or punctate white matter injury were excluded from the study. Additionally, 44 children (22 preterm and 22 full-term), recruited at Hanyang University Hospital, underwent standardized cognitive testing at a mean age of 4.45 years using the Wechsler Preschool and Primary Scale of Intelligence, Fourth Edition (WPPSI-IV), administered by trained examiners.

Of the 280 eligible participants, 18 were excluded from the morphometric analysis because of motion artefacts and poor image quality. A total of 262 participants were recruited

For Review Only

157 for the morphometric analysis using suitable MRI data obtained at 1–7 years of postnatal age (PNA). An overview of cohort size, MRI eligibility, and outcome availability is summarized in Supplementary Fig. S1. The study protocol was prospectively approved by the Institutional Review Board of Hanyang University Hospital, and written informed consent was obtained from parents or legal guardians at the time of neonatal enrollment as part of the hospital's neonatal follow-up program. For the Jeonbuk National University Hospital cohorts, the Institutional Review Board granted retrospective approval for the use of de-identified clinical and imaging data, in accordance with the principles outlined in the Declaration of Helsinki.

MRI acquisition

The present study was based on MRI data collected from Jeonbuk University Hospital, and Hanyang University Hospital was included to ensure alignment with the prospective neonatal MRI protocol. Because both hospitals participate in the Korean Neonatal Network, MRI acquisition was collaboratively standardized for research purposes, resulting in a unified single-vendor, single-protocol imaging framework. This approach maintained consistent imaging conditions across sites despite differences in data collection timing, thereby maximizing comparability between cohorts. Individual T1-weighted structural images were acquired using an MRI scanner (Philips Achieva 16-channel phase-array head coil; Best, Netherlands) with a magnetization-prepared rapid gradient echo (MPRAGE) sequence. An experienced pediatrician monitored the pulse oximeter during the MRI to determine the heart and respiratory rates of each participant. The parameters for T1-weighted images were TE = 3.39 ms, TR = 2.10 ms, TI = 1 ms, field of view = 200 mm^2, voxel sizes = 0.9 × 0.9 mm^2, slice thickness = 1 mm, and slice number = 150.

For Review Only

Structural data processing

We processed the T1-weighted structural MRI images using FreeSurfer version 7.4.1 (https://surfer.nmr.mgh.harvard.edu/).[54] The processing pipeline included bias field correction, motion and heterogeneity correction, transformation to the Talairach coordinate system, intensity normalization, skull stripping, white matter and grey matter tissue segmentation, white and pial surface reconstruction, and spherical mapping. Although there are relatively few studies focusing on children, FreeSurfer is still commonly used in this population.[55-57] After the cortical surface reconstruction, we applied a spherically deformed surface registration with minimal distortion.[58] The shape correspondence was then established using the registered spheres, and each sphere was resampled to the 7th level of the icosahedron subdivision (163,842 vertices).

Owing to the complexities involved in cortical surface parcellation in the developing brain, two independent researchers performed both automated and manual quality assessments of all reconstructed imaging data following several processes (Supplementary Text S1).

Morphological feature extraction

Two key morphological features were extracted from the reconstructed cortical surfaces, SD[59] and LGI[60] (Supplementary Fig. S2). For SD and LGI, the cerebral hull surface, which represents the outer contour of the cortex, was used as a reference. SD was defined as the shortest trajectory from the cerebral hull to the cortical surface, obtained by solving a constant-speed Eikonal equation between two surfaces.[59] Since SD is derived from a non-negative distance, its values are strictly positive and represent absolute geometric depth. LGI was computed using a shape-adaptive LGI introduced as a refinement of the conventional FreeSurfer's LGI.[60] A shape-adaptive LGI defines a spatially varying kernel that adapts to the local cortical folding. This is because the adaptive kernel is constructed through anisotropic wavefront propagation guided by a tensor field derived from cortical folding patterns.

For Review Only

Moreover, the kernel size is globally rescaled according to the cortical surface area across different age groups to account for brain size variability. Together, these adaptations show higher reproducibility in multi-scan dataset compared to FreeSurfer's conventional LGI. We applied Gaussian spatial smoothing with a full width at half maximum of 6 mm to mitigate the impact of noise on SD.[61]

Statistical analysis

Demographics

The demographics of the preterm and full-term infants were statistically compared using SPSS 27.0 (SPSS, Chicago, IL) software. We used the Mann–Whitney U test and chi-square analysis to compare the clinical factors between the preterm and full-term groups.

Statistical models

Linear mixed-effects models were designed to investigate group differences between preterm and full-term infants in terms of cortical measurements (SD, LGI) during early childhood. Cortical measurements were used as dependent variables, and the fixed effects comprised three covariates: PNA, sex, and GA. Although the MRI scanners and acquisition protocols were identical at both sites, potential nonbiological site-specific variations could confound subsequent analyses. To address this, we included hospitals as a random effect (random intercept) in the statistical models to absorb potential heterogeneity across sites and to estimate the true biological main and interaction effects more accurately. The following models were analyzed:

Preterm and full-term difference model. We examined the overall effect of cortical measurements that differed between preterm (PT) and full-term (FT) infants while controlling for PNA and sex. We test the following linear mixed model:

For Review Only

$$measure = \beta_0 + \beta_1(PT/FT) + \beta_2 PNA + \beta_3(PT/FT) * PNA + \beta_4 sex + u_{0j} + \epsilon \quad (1)$$

where β represents fixed effects, (PT/FT) represents a binary variable indicating full-term and preterm, u_{0j} denotes the hospital-specific random intercept that captures site-level variability and is assumed to follow $u_{0j} \sim N(0, \sigma_u^2)$, and ϵ represents random error.

GA subgroup differences model. We examined the overall effect of cortical measurements that differ between extremely-to-very preterm (GA < 32 weeks; E-VP) and late preterm (GA ≥ 32 weeks; LP) while controlling for PNA and sex. We test the following linear model:

$$measure = \beta_0 + \beta_1(E\_VP/LP) + \beta_2 PNA + \beta_3(E\_VP/LP) * PNA + \beta_4 sex + u_{0j} + \epsilon \quad (2)$$

where β represents fixed effects, (E_VP/LP) represents a binary variable indicating GA subgroup classification, u_{0j} denotes the hospital-specific random intercept that captures site-level variability and is assumed to follow $u_{0j} \sim N(0, \sigma_u^2)$, and ϵ represents random error.

Validation of linear age modeling. As an additional validation analysis to assess the appropriateness of the linear age term, we evaluated higher-order age effects and compared nested models. The full procedures and results of this supplementary validation are provided in Supplementary Text S2.

Implementation details. We conducted statistical analysis using SurfStat[62] a MATLAB toolbox that enables linear mixed-effects modeling,[63] random field theory statistical correction, and visualization of cortical surfaces. The statistical analysis was conducted via cortical surface registration on a vertex-wise basis, without the need for cortical parcellation. Cortical surface parcellation was used only for visualization purposes by projecting the results onto a template surface. In this process, FreeSurfer’s default cortical atlas, the Desikan-Killiany-Tourville cortical labeling protocol with 31 labels was used.[64] Statistical significance of parameters was

For Review Only

corrected for multiple comparisons using random field theory[65] at the level of 0.05 and cluster threshold (raw *p-value*) = 0.01.

Group comparisons of structure-function associations

To evaluate whether the associations between cortical measures and neurodevelopmental outcomes differed between the preterm and full-term groups, we used linear regression models incorporating a group × cortical measure interaction term. For each cortical metric (SD and LGI) that showed significant group differences, separate models were constructed for the VCI, VSI, FRI, WMI, and FSIQ scores from the WPPSI-IV. The general model specification was:

$$outcome = \beta_0 + \beta_1 measure + \beta_2 group + \beta_3 measure * group + \beta_4 PNA + \beta_5 sex + \epsilon \quad (3)$$

where outcome represents VCI, VSI, FRI, WMI, or FSIQ; measure refers to SD or LGI; and group is coded as PT vs. FT. Analyses were adjusted for PNA and sex, and for analyses involving preterm children, GA was additionally included as a covariate. The interaction coefficient (β_3) was used to test whether the association between cortical measures and cognitive outcomes differed significantly between the groups. All p-values were corrected for multiple comparisons using the FDR procedure.

For Review Only

Results

Participant characteristics

A total of 262 children were included in the study, comprising 56 preterm (mean age, 4.61 ± 1.57 years) and 206 full-term (mean age, 4.36 ± 1.77) participants. The participants were grouped as follows: 8 participants aged 1 years, including 8 full-term (mean age, 1.60 years); 38 participants aged 2 years, including 8 preterm and 34 full-term (mean age, 2.35 years); 50 at age 3 (15 preterm; 35 full-term; mean age, 3.43); 36 at age 4 (8 preterm; 28 full-term; mean age, 4.45); 33 at age 5 (8 preterm; 25 full-term; mean age, 5.51); 51 at age 6 (9 preterm; 42 full-term; mean age, 6.46); and 54 at age 7 (12 preterm; 42 full-term; mean age, 7.36) (Supplementary Fig. S3).

The mean GA was significantly lower in the preterm group than in the full-term group (31.43 ± 3.89 weeks vs. 38.88 ± 1.80 weeks, $P < 0.001$). No significant differences in PNA were observed between groups (4.61 ± 1.57 years vs. 4.36 ± 1.77 years, $P = 0.438$). The proportion of male participants did not significantly differ between the groups (71.4% vs. 59.7%, $P = 0.147$). No significant differences were observed in maternal education levels between the preterm and full-term groups in any category (<12 years, $P = 0.696$; <16 years, $P = 1.000$; >16 years, $P = 1.000$). At follow-up, cognitive performance was assessed using the WPPSI-IV in a subgroup of participants (22 pre-term and 22 full-term children). Compared with their full-term peers, preterm children demonstrated significantly lower scores in Verbal Comprehension Index (VCI: 80.23 ± 24.24 vs. 94.41 ± 12.93, $P = 0.009$), Visual Spatial Index (VSI: 83.95 ± 20.08 vs. 101.77 ± 16.18, $P = 0.002$), Fluid Reasoning Index (FRI: 81.17 ± 21.15 vs. 100.62 ± 17.30, $P = 0.017$), and Working Memory Index (WMI: 82.91 ± 23.97 vs. 99.65 ± 15.03, $P = 0.011$), and Full Scale IQ (FSIQ) was significantly lower in the preterm group (76.45 ± 23.32 vs. 98.14 ± 15.39, $P < 0.001$). Processing Speed Index (PSI) did not show a statistically

For Review Only

significant difference between the groups (80.27 ± 22.73 vs. 87.91 ± 18.81, $P = 0.401$). Supplementary Table S1 shows the characteristics of the participants, including perinatal factors and neurodevelopmental outcomes.

Interpretations of LGI and SD

We performed a combined analysis of LGI and SD to capture these distinct aspects of cortical morphology, as LGI alone cannot distinguish between sulcal depth and width changes.[38] Based on the present results, we noted three characteristic region-specific folding alterations: concurrent reductions in LGI and SD, reduced LGI with preserved SD, and decreased SD while maintaining the width-to-depth ratio (Fig. 1).

Preterm and full-term group differences analysis

Statistical analysis revealed significant differences in cortical measurements between preterm and full-term infants in different regions, as shown in Fig. 2.

The full-term group exhibited significantly higher LGI in seven clusters: the right superior temporal gyrus (STG; anterior and posterior parts), left STG, left superior frontal gyrus (SFG; anterior and middle parts), right posterior cingulate (PCG), isthmus cingulate gyrus (ICG), and right lateral occipital region. SD was significantly higher in the three clusters localized to both the STG and left SFG regions (Table 1). The LGI was significantly higher in the preterm group in one cluster and was localized to the left ICG. No significant differences were observed in SD in this contrast.

Scatterplots for all the identified clusters are shown in Supplementary Fig. S4 (A and B). Most regions showed a lower LGI and/or SD in the preterm group from 1 to 7 years of age. However, LGI in the preterm group showed a significantly greater increase in PNA in the left supramarginal region. SD showed a significantly greater increase in PNA than the preterm group in one cluster localized to the left precentral gyrus (PreCG) (Fig. 3).

For Review Only

E-VP and LP group differences analysis in preterm children

Statistical analysis revealed significant differences in the cortical measurements between the E-VP and LP groups at different regions, as shown in Fig. 4. Scatterplots illustrating all identified clusters are presented in Supplementary Fig. S4 (C and D).

LGI was significantly lower in the E-VP group in two clusters localized to the right STG and left LING. SD was significantly lower in one cluster located in the right STG. No statistically significant clusters were observed for LGI or SD in the E-VP > LP contrast (Table 2).

When examining PNA-related subgroup differences, only one statistically significant cluster was found for LGI, where the E-VP group showed a significantly greater increase in PNA compared to the E-VP group in the right PreCG. No significant interaction effects were observed for SD (Fig. 5).

Group differences in associations between cortical measurements and neurodevelopmental outcomes

Statistical analysis revealed group differences in the associations between cortical measures and neurodevelopmental outcomes based on linear models incorporating group × cortical measure interaction terms (Table 3). In the right superior temporal region, the interaction between SD and VCI was significant (β = –18.48, FDR p = 0.014), whereas the interaction for LGI did not reach statistical significance (β = –11.53, FDR p = 0.822). For WMI, LGI showed a nominal interaction effect (β = –32.47, FDR p = 0.129), while SD demonstrated a significant interaction (β = –20.62, FDR p = 0.014). For FSIQ, the interaction effect was not significant for LGI (β = –19.72, FDR p = 0.543), but SD showed a significant interaction (β = –17.65,

For Review Only

FDR p = 0.031). In the left superior temporal region, LGI demonstrated a nominal interaction effect with WMI ($\beta = -19.95$, FDR $p = 0.129$), whereas SD exhibited a significant interaction ($\beta = -23.51$, FDR $p = 0.004$). Full results are provided in Supplementary Tables S2 and S3.

For Review Only

Discussion

In this study, we applied a cortical surface registration technique to establish intersubject anatomical correspondence and minimize registration-induced distortions. This anatomically precise alignment enabled robust group-wise comparisons of cortical morphometry in early childhood, revealing regionally specific deviations in cortical folding among preterm children. Compared with their full-term counterparts, preterm infants exhibited significantly reduced LGI and SD in the bilateral STG and left SFG, suggesting a region-specific disruption of perinatal cortical morphogenesis. In the right ICG and PCG, the reduced LGI suggested incomplete gyral expansion during secondary and tertiary folding in early infancy. These alterations persisted from the ages of 1 to 7 years, indicating enduring deviations from normative folding trajectories.

Compared to the full-term group, the preterm group exhibited lower LGI and SD in both the STG and left SFG, reflecting altered gyral and sulcal maturation that could potentially restrict experience-dependent plasticity in higher-order cognitive regions. The STG and SFG emerge at approximately 20 weeks GA and undergo rapid morphological changes at approximately 24 weeks.[66] Previous studies using surface-based analyses of cortical gyrification in healthy preterm and full-term infants have shown that folding progression is particularly marked within temporal and frontal association cortices across the perinatal period[11,67], and this regionally elevated growth trajectory appears to extend into the first two years after birth.[15] This developmental profile aligns with a hierarchical maturation process in which association cortices gradually mature over an extended period through synaptic pruning, progressive myelination, and the establishment of long-range associative connections.[25,68,69] Moreover, the expansion of axonal and synaptic architecture imposes increasing tangential growth demands on the cortical sheet, which can manifest as gyrification patterns that facilitate

For Review Only

the efficient spatial embedding of distributed networks.[70] Together, these folding mechanisms form a structural basis that enables experience-dependent refinement after birth and supports the emergence of higher-order cognitive functions. Interestingly, Hill *et al.*[71] proposed that cortical regions related to higher-order cognitive processing maintain comparatively low levels of brain morphological maturity during the fetal period to preserve as window for postnatal experience-dependent neuroplasticity. Ronan *et al.*[72] further reported that these regions undergo spatially heterogeneous tangential expansion after birth, suggesting that such variability may be a typical developmental feature supporting experience-dependent refinement. Given that gyrification is informative in capturing structural variability across cortical regions[73-75], reductions in LGI and SD observed in the STG and SFG suggest that preterm birth may be associated with delayed or incomplete structural maturation, potentially limiting the engagement of neuroplastic mechanisms in regions subserving higher-order cognitive functions. Our suggestions are supported by previous studies. Ball *et al.*[25] suggested that cortical alterations in the STG and SFG observed in preterm infants are associated with genes regulating early maturing inhibitory neurons and that this may fail to follow the hierarchical maturation map after preterm birth. From a macroscopic perspective, Engelhardt *et al.*[76] reported that preterm infants exhibit reduced surface area and lower GIs compared to full-term infants, particularly in the STG and its adjacent regions.

Moreover, Papini *et al.*[77] found reduced LGI in the STG and SFG among adults born very preterm and suggested that early alterations in neural substrates due to preterm birth may lead to qualitative differences in the relationship between cortical folding and adult mental health outcomes. Similarly, we found that the association between SD and VCI, WMI, and FSIQ was markedly reduced in the preterm children (with a similar pattern for WMI in the left STG). These results highlight that preterm birth not only alters folding morphology but also that the atypical folding patterns may associated with early extrauterine exposure can fail to

For Review Only

confer, and may even diminish, the neurodevelopmental advantages observed during normative development. Given that the STG functions as a hub for higher-order auditory associations, aberrant folding in this region could perturb auditory–language pathways and, in turn, manifest as differences in language, verbal working memory, and general intellectual ability.[78-80]

The right STG, identified as differing in both LGI and SD between the preterm and full-term groups, was significantly reduced in the E-VP group in the preterm subgroup analysis, extending the influence of GA on cortical development. Extrauterine exposure in infants with E-VP overlaps with a critical period of early cortical folding, during which cumulative stress may exert long-lasting effects on the neurobiological substrates of gyrification. Collectively, these findings demonstrate that disrupted gyral maturation limits experience-dependent plasticity normally engaged in the temporal association cortices, thereby contributing to altered neurodevelopmental trajectories in preterm infants.

The right ICG and PCG in preterm children displayed a pattern of reduced LGI with preserved SD, characterized by relatively wide but not shallow sulci, indicating simplified higher-order folding due to the disrupted elaboration of secondary and tertiary cortical folds. These atypical cortical folding patterns may be interpreted within the context of the following mechanistic frameworks. First, the differential growth hypothesis posits that asynchronous expansion between cortical layers and adjacent regions leads to folding.[81] Preterm birth may disrupt this equilibrium, limiting the mechanical tension and geometric conditions required for complex cortical folding, particularly the secondary and tertiary folds that typically develop during late gestation. Recent developmental evidence suggests that preterm infants fail to follow the typical trajectory of asynchronous cortical expansion associated with the migration of subplate neurons during the formation of complex secondary and tertiary folds.[11] Given that the cingulate sulcus continues to stabilize structurally beyond the age of seven,[82] early disturbances may result in long-lasting deviations from normative morphogenesis.[19] Second,

For Review Only

according to the tension-based theory, the folding pattern is shaped by mechanical tension generated through long-distance axonal connectivity,[12] and premature birth may weaken these forces owing to reduced myelination and impaired axonal integrity.[83] Supporting this view, previous neuroimaging studies have reported that the cingulate sulcus in very preterm children is shorter and more fragmented than that in their full-term peers, suggesting incomplete development of long-range connectivity.[84] Moreover, the cingulum bundle plays a crucial role in integrating emotional and attentional processing by connecting the medial prefrontal, parietal, and temporal regions, thereby facilitating communication across the networks involved in self-referential thinking, memory retrieval, executive functioning, and emotional regulation.[85-87] Disruption of the integrity or morphogenesis of the cingulate cortex and its associated white matter pathways may compromise a wide range of socioemotional outcomes observed in children born preterm.[83] This converging evidence suggests the potential of ICG and PCG as biomarkers for long-term psychosocial outcomes in preterm infants.

PNA-related cortical maturation of the left PreCG (in SD) showed divergent intergroup developmental patterns during the perinatal period but converged around 5–6 years of age. These regions, the primary visual and motor cortices, are characterized by early structural maturation and low inter-individual variability, as shown in previous studies,[4,7,15,87] and are therefore considered structurally stable during early cortical development. Nevertheless, divergent patterns of early cortical development may reflect altered cortical maturation induced by extrauterine exposure, including disrupted sensory-driven cortical morphogenesis or delayed thalamocortical connectivity in the sensorimotor cortex, which may ultimately impair cortical folding and the efficiency of neural communication through white matter pathways.[88,89] Consequently, the subsequent convergence of developmental trajectories may reflect experience-dependent neuroplasticity and delayed alignment with normative cortical growth patterns in preterm infants. Notably, strong thalamocortical inputs and preserved structural

For Review Only

scaffolding may have contributed to the catch-up maturation.[90] These findings align with those of previous reports indicating compensatory development in sensorimotor hubs[91-93] and suggest that such regions may provide a foundation for the emergence of higher-order cognitive functions.[33,35,94,95]

Technically, traditional methods for computing LGI[54] capture the overall cortical folding patterns reasonably well, but they are sometimes unable to reflect the finer details of local cortical morphology, as they do not explicitly incorporate folding patterns into the computation.[60] To address this limitation, we employed a shape-adaptive LGI[60] method that better reflected the local folding patterns of the cortex. This method has demonstrated improved sensitivity to region-specific developmental changes, particularly those not identified by traditional approaches.[38,96] It is also well-suited for pediatric populations that exhibit substantial inter-individual variability.

Despite the strengths of this study, it has some clinical limitations that must be acknowledged. First, the cross-sectional design limited our ability to directly infer longitudinal developmental trajectories or track intra-individual changes in cortical morphology over time. As such, although we observed age-related patterns and group differences, these findings cannot definitively establish the causality or temporal dynamics of folding development. Second, the preterm sample size was not sufficiently large to capture the broad developmental window (1–7 years) during which cortical maturation progresses rapidly, which may reduce the precision of age-related inferences. These results should be interpreted cautiously and validated in larger cohorts. Third, only a subset of children completed cognitive assessments, limiting the robustness of neurodevelopmental associations. These results should be viewed as preliminary and require replication with broader behavioral sampling. Fourth, our analysis was based solely on T1-weighted imaging and did not include concurrent diffusion tension imaging

For Review Only

462 or other white matter-sensitive modalities. As a result, we were limited in our ability to interpret
463 the observed cortical folding changes in the context of white matter integrity or long-range
464 axonal connectivity,[90,97] both of which are thought to play mechanistic roles in cortical
465 morphogenesis.[36,98,99] Multimodal approaches that integrate structural, diffusion, and
466 functional imaging would offer a more comprehensive understanding of the
467 neurodevelopmental consequences of premature birth.

468 One methodological limitation concerns the choice of the cluster threshold in multiple
469 comparison correction. In this study, we applied a relatively liberal threshold of $p < 0.01$, which
470 differs from the conventional standard in surface-based neuroimaging ($p < 0.001$). This
471 decision was made to account for the limited sample size of the preterm group, which
472 substantially reduced statistical power and made it difficult to detect significant clusters after
473 multiple comparison correction. We acknowledge that this approach increases the potential risk
474 of false positives. To address this issue, we additionally provide results based on a stricter
475 threshold of $p < 0.001$ in the Supplementary Materials and discuss the discrepancies between
476 the two results as an important limitation (Supplementary Fig. S5-8). Future studies with larger
477 sample sizes are expected to resolve this limitation.

478 Overall, our findings underscore the fact that preterm birth induces region-specific
479 disruptions in cortical folding that persist into early childhood and influence structure–function
480 relationships. These alterations exhibit vulnerability that becomes more evident along the
481 functional hierarchy from the primary sensorimotor to the higher-order associative cortices.
482 While certain primary sensorimotor areas, such as the preCG and supramarginal gyrus, exhibit
483 signs of structural recovery, regions implicated in higher-order cognitive processing, including
484 the SFG, STG, and PCG, appear to be persistently altered. The use of folding-sensitive markers

For Review Only

such as LGI and SD provides critical insights into the long-term neurodevelopmental consequences of prematurity and supports the design of regionally targeted interventions.

Data availability

The cohort datasets generated and/or analyzed during the current study are not publicly available because of the inability to share personal information according to research ethics but are available from the corresponding author upon reasonable request. Correspondence and requests for materials should be addressed to YHJ (ryanjang93@hanyang.ac.kr) and HJL (blesslee77@hanmail.net). All codes and computational tools used in this study are publicly accessible. The shape-adaptive local gyrification index algorithm is available at: https://github.com/ilwoolyu/LocalGyrificationIndex. The hierarchical spherical deformation framework for cortical surface registration is available at: https://github.com/ilwoolyu/HSD. Statistical analyses were performed using SurfStat (https://www.math.mcgill.ca/keith/surfstat/). A Docker image that integrates cortical morphometry tools, including local gyrification index, sulcal depth, and hierarchical spherical deformation, is available at: https://hub.docker.com/r/ilwoolyu/cmorph. These code resources are also provided in the Supplementary Materials.

For Review Only

Funding

This work was supported in part by the National Research Foundation of Korea (NRF) under RS-2023-NR077125, RS-2024-00333931, and RS-2025-02216257; in part by the Institute for Information & Communications Technology Planning & Evaluation (IITP) AIGS Program under RS-2019-II191906.

Competing interests

The authors report no competing interests.

Supplementary material

Supplementary material is available at *Brain communications* online. The supplementary material is included in a separate PDF file.

References

1. Cao B, Mwangi B, Passos IC*, et al*. Lifespan gyrification trajectories of human brain in healthy individuals and patients with major psychiatric disorders. *Scientific Reports*. 2017;7(1):511.

2. White T, Su S, Schmidt M, Kao C-Y, Sapiro G. The development of gyrification in childhood and adolescence. *Brain and cognition*. 2010;72(1):36-45.

3. Auzias G, Brun L, Deruelle C, Coulon O. Deep sulcal landmarks: algorithmic and conceptual improvements in the definition and extraction of sulcal pits. *Neuroimage*. 2015;111:12-25.

4. Chi JG, Dooling EC, Gilles FH. Gyral development of the human brain. *Annals of Neurology: Official Journal of the American Neurological Association and the Child Neurology Society*. 1977;1(1):86-93.

5. Garel C, Chantrel E, Brisse H*, et al*. Fetal cerebral cortex: normal gestational landmarks identified using prenatal MR imaging. *American Journal of Neuroradiology*. 2001;22(1):184-189.

6. Habas PA, Scott JA, Roosta A*, et al*. Early folding patterns and asymmetries of the normal human brain detected from in utero MRI. *Cerebral cortex*. 2012;22(1):13-25.

7. Im K, Jo HJ, Mangin J-F, Evans AC, Kim SI, Lee J-M. Spatial distribution of deep sulcal landmarks and hemispherical asymmetry on the cortical surface. *Cerebral cortex*. 2010;20(3):602-611.

8. Le Guen Y, Auzias G, Leroy F*, et al*. Genetic influence on the sulcal pits: on the origin of the first cortical folds. *Cerebral Cortex*. 2018;28(6):1922-1933.

9. Rajagopalan V, Scott J, Habas PA*, et al*. Local tissue growth patterns underlying normal fetal human brain gyrification quantified in utero. *Journal of neuroscience*. 2011;31(8):2878-2887.

10. Yun HJ, Vasung L, Tarui T*, et al*. Temporal patterns of emergence and spatial distribution of sulcal pits during fetal life. *Cerebral Cortex*. 2020;30(7):4257-4268.

11. Dubois J, Lefèvre J, Angleys H*, et al*. The dynamics of cortical folding waves and prematurity-related deviations revealed by spatial and spectral analysis of gyrification. *Neuroimage*. 2019;185:934-946.

12. Essen DCv. A tension-based theory of morphogenesis and compact wiring in the central nervous system. *Nature*. 1997;385(6614):313-318.

13. Tallinen T, Chung JY, Biggins JS, Mahadevan L. Gyrification from constrained cortical expansion. *Proceedings of the National Academy of Sciences*. 2014;111(35):12667-12672.

For Review Only

14. Tallinen T, Chung JY, Rousseau F, Girard N, Lefèvre J, Mahadevan L. On the growth and form of cortical convolutions. *Nature Physics*. 2016;12(6):588-593.

15. Li G, Wang L, Shi F*, et al*. Mapping longitudinal development of local cortical gyrification in infants from birth to 2 years of age. *Journal of Neuroscience*. 2014;34(12):4228-4238.

16. Lenroot RK, Gogtay N, Greenstein DK*, et al*. Sexual dimorphism of brain developmental trajectories during childhood and adolescence. *Neuroimage*. 2007;36(4):1065-1073.

17. Nie J, Li G, Shen D. Development of cortical anatomical properties from early childhood to early adulthood. *Neuroimage*. 2013;76:216-224.

18. Raznahan A, Shaw P, Lalonde F*, et al*. How does your cortex grow? *Journal of Neuroscience*. 2011;31(19):7174-7177.

19. Lefèvre J, Germanaud D, Dubois J*, et al*. Are developmental trajectories of cortical folding comparable between cross-sectional datasets of fetuses and preterm newborns? *Cerebral cortex*. 2015;26(7):3023-3035.

20. Volpe JJ. Dysmaturation of premature brain: importance, cellular mechanisms, and potential interventions. *Pediatric neurology*. 2019;95:42-66.

21. Fleiss B, Gressens P, Stolp HB. Cortical gray matter injury in encephalopathy of prematurity: link to neurodevelopmental disorders. *Frontiers in Neurology*. 2020;11:575.

22. Miller SP, Ferriero DM. From selective vulnerability to connectivity: insights from newborn brain imaging. *Trends in neurosciences*. 2009;32(9):496-505.

23. Rathbone R, Counsell S, Kapellou O*, et al*. Perinatal cortical growth and childhood neurocognitive abilities. *Neurology*. 2011;77(16):1510-1517.

24. Volpe JJ. Encephalopathy of prematurity includes neuronal abnormalities. *Pediatrics*. 2005;116(1):221-225.

25. Ball G, Seidlitz J, O'Muircheartaigh J*, et al*. Cortical morphology at birth reflects spatiotemporal patterns of gene expression in the fetal human brain. *PLoS biology*. 2020;18(11):e3000976.

26. Ajayi-Obe M, Saeed N, Cowan F, Rutherford MA, Edwards AD. Reduced development of cerebral cortex in extremely preterm infants. *The Lancet*. 2000;356(9236):1162-1163.

27. Bouyssi-Kobar M, Brossard-Racine M, Jacobs M, Murnick J, Chang T, Limperopoulos C. Regional microstructural organization of the cerebral cortex is affected by preterm birth. *NeuroImage: Clinical*. 2018;18:871-880.

28. Kelly CE, Thompson DK, Adamson CL*, et al*. Cortical growth from infancy to adolescence in preterm and term-born children. *Brain*. 2024;147(4):1526-1538.

29. Makropoulos A, Aljabar P, Wright R*, et al*. Regional growth and atlasing of the developing human brain. *Neuroimage*. 2016;125:456-478.

30. Monson BB, Anderson PJ, Matthews LG*, et al*. Examination of the pattern of growth of cerebral tissue volumes from hospital discharge to early childhood in very preterm infants. *JAMA pediatrics*. 2016;170(8):772-779.

For Review Only

31. Nam KW, Castellanos N, Simmons A*, et al*. Alterations in cortical thickness development in preterm-born individuals: Implications for high-order cognitive functions. *NeuroImage*. 2015;115:64-75.

32. Rimol LM, Bjuland KJ, Løhaugen GC*, et al*. Cortical trajectories during adolescence in preterm born teenagers with very low birthweight. *Cortex*. 2016;75:120-131.

33. Sripada K, Bjuland KJ, Sølsnes AE*, et al*. Trajectories of brain development in school-age children born preterm with very low birth weight. *Scientific reports*. 2018;8(1):15553.

34. Thompson DK, Matthews LG, Alexander B*, et al*. Tracking regional brain growth up to age 13 in children born term and very preterm. *Nature communications*. 2020;11(1):696.

35. Vandewouw MM, Young JM, Mossad SI*, et al*. Mapping the neuroanatomical impact of very preterm birth across childhood. *Human brain mapping*. 2020;41(4):892-905.

36. Nie J, Li G, Wang L*, et al*. Longitudinal development of cortical thickness, folding, and fiber density networks in the first 2 years of life. *Human brain mapping*. 2014;35(8):3726-3737.

37. Richards JE, Xie W. Brains for all the ages: structural neurodevelopment in infants and children from a life-span perspective. *Advances in child development and behavior*. 2015;48:1-52.

38. Zoltowski AR, Lyu I, Failla M*, et al*. Cortical morphology in autism: findings from a cortical shape-adaptive approach to local gyrification indexing. *Cerebral Cortex*. 2021;31(11):5188-5205.

39. Schaer M, Cuadra MB, Tamarit L, Lazeyras F, Eliez S, Thiran JP. A surface-based approach to quantify local cortical gyrification. *IEEE Trans Med Imaging*. Feb 2008;27(2):161-70. doi:10.1109/tmi.2007.903576

40. Akula SK, Exposito-Alonso D, Walsh CA. Shaping the brain: The emergence of cortical structure and folding. *Developmental cell*. 2023;58(24):2836-2849.

41. Kostović I, Jovanov-Milošević N. The development of cerebral connections during the first 20–45 weeks' gestation. Elsevier; 2006:415-422.

42. Sidman RL, Rakic P. Neuronal migration, with special reference to developing human brain: a review. *Brain research*. 1973;62(1):1-35.

43. Xu X, Sun C, Sun J*, et al*. Spatiotemporal atlas of the fetal brain depicts cortical developmental gradient. *Journal of Neuroscience*. 2022;42(50):9435-9449.

44. Dubois J, Benders M, Cachia A*, et al*. Mapping the early cortical folding process in the preterm newborn brain. *Cerebral cortex*. 2008;18(6):1444-1454.

45. Armstrong E, Schleicher A, Omran H, Curtis M, Zilles K. The ontogeny of human gyrification. *Cerebral cortex*. 1995;5(1):56-63.

46. Kim SH, Lyu I, Fonov VS*, et al*. Development of cortical shape in the human brain from 6 to 24 months of age via a novel measure of shape complexity. *NeuroImage*. 2016;135:163-176.

47. Luders E, Narr KL, Thompson PM*, et al*. Gender differences in cortical complexity. *Nature neuroscience*. 2004;7(8):799-800.

For Review Only

48. Lui JH, Hansen DV, Kriegstein AR. Development and evolution of the human neocortex. *Cell*. 2011;146(1):18-36.

49. Zilles K, Armstrong E, Schleicher A, Kretschmann H-J. The human pattern of gyrification in the cerebral cortex. *Anatomy and embryology*. 1988;179:173-179.

50. Palaniyappan L, Liddle PF. Aberrant cortical gyrification in schizophrenia: a surface-based morphometry study. *Journal of Psychiatry and Neuroscience*. 2012;37(6):399-406.

51. Mirakhur A, Moorhead TW, Stanfield AC*, et al*. Changes in gyrification over 4 years in bipolar disorder and their association with the brain-derived neurotrophic factor valine(66) methionine variant. *Biol Psychiatry*. Aug 1 2009;66(3):293-7. doi:10.1016/j.biopsych.2008.12.006

52. Zhang Y, Yu C, Zhou Y, Li K, Li C, Jiang T. Decreased gyrification in major depressive disorder. *Neuroreport*. Mar 4 2009;20(4):378-80. doi:10.1097/WNR.0b013e3283249b34

53. Molent C, Maggioni E, Cecchetto F*, et al*. Reduced cortical thickness and increased gyrification in generalized anxiety disorder: a 3 T MRI study. *Psychol Med*. Sep 2018;48(12):2001-2010. doi:10.1017/s003329171700352x

54. Fischl B. FreeSurfer. *Neuroimage*. Aug 15 2012;62(2):774-81. doi:10.1016/j.neuroimage.2012.01.021

55. Beelen C, Phan TV, Wouters J, Ghesquière P, Vandermosten M. Investigating the added value of FreeSurfer's manual editing procedure for the study of the reading network in a pediatric population. *Frontiers in human neuroscience*. 2020;14:143.

56. Pulli EP, Silver E, Kumpulainen V*, et al*. Feasibility of FreeSurfer processing for T1-weighted brain images of 5-year-olds: semiautomated protocol of FinnBrain Neuroimaging Lab. *Frontiers in Neuroscience*. 2022;16:874062.

57. Wedderburn CJ, Subramoney S, Yeung S*, et al*. Neuroimaging young children and associations with neurocognitive development in a South African birth cohort study. *Neuroimage*. 2020;219:116846.

58. Lyu I, Kang H, Woodward ND, Styner MA, Landman BA. Hierarchical spherical deformation for cortical surface registration. *Med Image Anal*. Oct 2019;57:72-88. doi:10.1016/j.media.2019.06.013

59. Lyu I, Kang H, Woodward ND, Landman BA. Sulcal Depth-based Cortical Shape Analysis in Normal Healthy Control and Schizophrenia Groups. *Proc SPIE Int Soc Opt Eng*. Mar 2018;10574doi:10.1117/12.2293275

60. Lyu I, Kim SH, Girault JB, Gilmore JH, Styner MA. A cortical shape-adaptive approach to local gyrification index. *Med Image Anal*. Aug 2018;48:244-258. doi:10.1016/j.media.2018.06.009

61. Han X, Jovicich J, Salat D*, et al*. Reliability of MRI-derived measurements of human cerebral cortical thickness: the effects of field strength, scanner upgrade and manufacturer. *Neuroimage*. Aug 1 2006;32(1):180-94. doi:10.1016/j.neuroimage.2006.02.051

62. Worsley KJ, Taylor JE, Carbonell F*, et al*. SurfStat: A Matlab toolbox for the statistical analysis of univariate and multivariate surface and volumetric data using linear mixed effects models and random field theory. *NeuroImage*. 2009/07/01/ 2009;47:S102. doi:https://doi.org/10.1016/S1053-8119(09)70882-1

For Review Only

63. Bates D, Mächler M, Bolker B, Walker S. Fitting Linear Mixed-Effects Models Using lme4. *Journal of Statistical Software*. 10/07 2015;67(1):1 - 48. doi:10.18637/jss.v067.i01

64. Klein A, Tourville J. 101 labeled brain images and a consistent human cortical labeling protocol. *Frontiers in neuroscience*. 2012;6:171.

65. Hagler DJ, Jr., Saygin AP, Sereno MI. Smoothing and cluster thresholding for cortical surface-based group analysis of fMRI data. *Neuroimage*. Dec 2006;33(4):1093-103. doi:10.1016/j.neuroimage.2006.07.036

66. Yun HJ, Lee HJ, Lee JY*, et al*. Quantification of sulcal emergence timing and its variability in early fetal life: hemispheric asymmetry and sex difference. *NeuroImage*. 2022;263:119629.

67. Orasanu E, Melbourne A, Cardoso MJ*, et al*. Cortical folding of the preterm brain: a longitudinal analysis of extremely preterm born neonates using spectral matching. *Brain and behavior*. 2016;6(8):e00488.

68. Sowell ER, Thompson PM, Leonard CM, Welcome SE, Kan E, Toga AW. Longitudinal mapping of cortical thickness and brain growth in normal children. *J Neurosci*. Sep 22 2004;24(38):8223-31. doi:10.1523/jneurosci.1798-04.2004

69. Sydnor VJ, Larsen B, Bassett DS*, et al*. Neurodevelopment of the association cortices: Patterns, mechanisms, and implications for psychopathology. *Neuron*. 2021;109(18):2820-2846.

70. Striedter GF, Srinivasan S, Monuki ES. Cortical folding: when, where, how, and why? *Annual review of neuroscience*. 2015;38(1):291-307.

71. Hill J, Inder T, Neil J, Dierker D, Harwell J, Van Essen D. Similar patterns of cortical expansion during human development and evolution. *Proceedings of the National Academy of Sciences*. 2010;107(29):13135-13140.

72. Ronan L, Voets N, Rua C*, et al*. Differential tangential expansion as a mechanism for cortical gyrification. *Cereb Cortex*. Aug 2014;24(8):2219-28. doi:10.1093/cercor/bht082

73. Zilles K, Schleicher A, Langemann C*, et al*. Quantitative analysis of sulci in the human cerebral cortex: development, regional heterogeneity, gender difference, asymmetry, intersubject variability and cortical architecture. *Human brain mapping*. 1997;5(4):218-221.

74. White T, O'Leary D, Magnotta V, Arndt S, Flaum M, Andreasen NC. Anatomic and functional variability: the effects of filter size in group fMRI data analysis. *Neuroimage*. 2001;13(4):577-588.

75. Mangin J-F, Riviere D, Cachia A*, et al*. A framework to study the cortical folding patterns. *Neuroimage*. 2004;23:S129-S138.

76. Engelhardt E, Inder TE, Alexopoulos D*, et al*. Regional impairments of cortical folding in premature infants. *Annals of neurology*. 2015;77(1):154-162.

77. Papini C, Palaniyappan L, Kroll J, Froudist-Walsh S, Murray RM, Nosarti C. Altered cortical gyrification in adults who were born very preterm and its associations with cognition and mental health. *Biological Psychiatry: Cognitive Neuroscience and Neuroimaging*. 2020;5(7):640-650.

78. Bigler ED, Mortensen S, Neeley ES*, et al*. Superior temporal gyrus, language function, and autism. *Developmental neuropsychology*. 2007;31(2):217-238.

For Review Only

79. Park H, Kang E, Kang H, *et al*. Cross-frequency power correlations reveal the right superior temporal gyrus as a hub region during working memory maintenance. *Brain connectivity*. 2011;1(6):460-472.

80. Yi HG, Leonard MK, Chang EF. The encoding of speech sounds in the superior temporal gyrus. *Neuron*. 2019;102(6):1096-1110.

81. Toro R, Burnod Y. A morphogenetic model for the development of cortical convolutions. *Cerebral cortex*. 2005;15(12):1900-1913.

82. Cachia A, Borst G, Tissier C, *et al*. Longitudinal stability of the folding pattern of the anterior cingulate cortex during development. *Developmental cognitive neuroscience*. 2016;19:122-127.

83. Melbourne A, Kendall GS, Cardoso MJ, *et al*. Preterm birth affects the developmental synergy between cortical folding and cortical connectivity observed on multimodal MRI. *Neuroimage*. Apr 1 2014;89:23-34. doi:10.1016/j.neuroimage.2013.11.048

84. Zhang Y, Inder TE, Neil JJ, *et al*. Cortical structural abnormalities in very preterm children at 7 years of age. *Neuroimage*. 2015;109:469-479.

85. Bubb EJ, Metzler-Baddeley C, Aggleton JP. The cingulum bundle: Anatomy, function, and dysfunction. *Neuroscience & Biobehavioral Reviews*. 2018/09/01/ 2018;92:104-127. doi:https://doi.org/10.1016/j.neubiorev.2018.05.008

86. Leech R, Sharp DJ. The role of the posterior cingulate cortex in cognition and disease. *Brain*. 2014;137(1):12-32.

87. Remer J, Croteau-Chonka E, Dean DC, *et al*. Quantifying cortical development in typically developing toddlers and young children, 1–6 years of age. *NeuroImage*. 2017/06/01/ 2017;153:246-261. doi:https://doi.org/10.1016/j.neuroimage.2017.04.010

88. Cherniak C, Mokhtarzada Z, Rodriguez-Esteban R, Changizi K. Global optimization of cerebral cortex layout. *Proceedings of the National Academy of Sciences*. 2004;101(4):1081-1086.

89. Fischl B, Rajendran N, Busa E, *et al*. Cortical folding patterns and predicting cytoarchitecture. *Cerebral cortex*. 2008;18(8):1973-1980.

90. Rakic P. Specification of cerebral cortical areas. *Science*. 1988;241(4862):170-176.

91. Jang YH, Kim H, Lee JY, Ahn J-H, Chung AW, Lee HJ. Altered development of structural MRI connectome hubs at near-term age in very and moderately preterm infants. *Cerebral Cortex*. 2023;33(9):5507-5523.

92. Toulmin H, Beckmann CF, O'Muircheartaigh J, *et al*. Specialization and integration of functional thalamocortical connectivity in the human infant. *Proceedings of the National Academy of Sciences*. 2015;112(20):6485-6490.

93. Van den Heuvel MP, Sporns O. Network hubs in the human brain. *Trends in cognitive sciences*. 2013;17(12):683-696.

94. Karolis VR, Froudist-Walsh S, Kroll J, *et al*. Volumetric grey matter alterations in adolescents and adults born very preterm suggest accelerated brain maturation. *Neuroimage*. 2017;163:379-389.

95. Mürner-Lavanchy I, Steinlin M, Nelle M, *et al*. Delay of cortical thinning in very preterm born children. *Early human development*. 2014;90(9):443-450.

For Review Only

96. Stoebner ZA, Hett K, Lyu I*, et al*. Comprehensive shape analysis of the cortex in Huntington's disease. *Human brain mapping*. 2023;44(4):1417-1431.

97. O'Leary DD, Schlaggar BL, Tuttle R. Specification of neocortical areas and thalamocortical connections. *Annual review of neuroscience*. 1994;17(1):419-439.

98. Yap P-T, Fan Y, Chen Y, Gilmore JH, Lin W, Shen D. Development trends of white matter connectivity in the first years of life. *PloS one*. 2011;6(9):e24678.

99. Zhu D, Li K, Guo L*, et al*. DICCCOL: dense individualized and common connectivity-based cortical landmarks. *Cerebral cortex*. 2013;23(4):786-800.

For Review Only

Figure legends

Figure 1. Influence of sulcal width and depth on LGI. Variations in LGI can result from changes in sulcal depth, width, or their combination. To analyze different scenarios, consider three characteristics: **A.** half depth (both LGI and SD decreases); **B.** double width (only LGI decreases); **C.** half width and depth (only SD decreases, and LGI does not necessarily decrease). Thus, sulcal depth and LGI need to be considered together to explicitly explain cortical folding differences. Abbreviations: LGI, local gyrification index.

For Review Only

Figure 2. Preterm and full-term group differences in cortical measurements. Regions of statistically significant group differences in LGI (A) and SD (B) are shown, colored according to cluster corrected p-value (bottom scale). 1st and 3rd row indicate regions of lesser LGI and SD in the preterm group than in the full-term group, and vice versa in the 2nd row, at the $P < 0.05$ level after correcting for multiple comparisons via random field theory. Clusters were highlighted with a purple circle when both LGI and SD are significant and highlighted with a red circle when only LGI is significant. The scatter plot displays individual data points with fitted regression lines for each group, and the corresponding statistical test results (t-value, group sizes, p-value, FDR-corrected p-value, and interaction p-value) are reported within the figure. Abbreviations: FT, full-term; PT, preterm; LGI, local gyrification index; SD, sulcal depth.

For Review Only

Figure 3. Statistically significant regions of PNA by group interactions by cortical measurements. Regions of statistically significant PNA by group differences in LGI (A) and SD (B) are shown, colored according to cluster p-value (Bottom scale). First row indicates regions of lesser slope with PNA in the preterm group than in the full-term group and vice versa in the remaining two rows, at the $P < 0.05$ level after correcting for multiple comparisons via random field theory. Clusters were highlighted with a red circle when only LGI is significant and highlighted with a blue circle when only SD is significant. Abbreviations: PNA, postnatal age; FT, full-term; PT, preterm; LGI, local gyrification index; SD, sulcal depth.

For Review Only

Figure 4. Preterm subgroup differences in cortical measurements. Regions of statistically significant group differences in LGI (A) and SD (B) are shown, colored according to cluster corrected p-value (bottom scale). The results indicate regions of lesser LGI and SD in the E-VP group than in the LP group, at the $P < 0.05$ level after correcting for multiple comparisons via random field theory. Clusters were highlighted with a purple circle when both LGI and SD are significant and highlighted with a red circle when only LGI is significant. The scatter plot displays individual data points with fitted regression lines for each group, and the corresponding statistical test results (t-value, group sizes, p-value, FDR-corrected p-value, and interaction p-value) are reported within the figure. Abbreviations: LP, late preterm; E-VP, extremely-to-very preterm; LGI, local gyrification index; SD, sulcal depth.

For Review Only

Figure 5. Statistically significant regions of PNA by preterm subgroup interactions by cortical measurements. Regions of statistically significant PNA by group differences in LGI. The results indicate regions where the slope with PNA is less in the E-VP group than in the LP group for LGI, at the $P < 0.05$ level after correcting for multiple comparisons via random field theory. The cluster was highlighted with a red circle when only LGI is significant. Abbreviations: PNA, postnatal age; LP, late preterm; E-VP, extremely-to-very preterm; LGI, local gyrification index.

For Review Only

Table 1. Group Differences in Cortical Measurements

Index	Cluster	Region	Adj. P-value
**FT > PT**			
LGI	1	Right superior temporal (anterior part)	0.0001<
	2	Left superior frontal (anterior part)	0.0001<
	3	Right posterior cingulate, isthmus cingulate	0.0002
	4	Left superior temporal	0.0003
	5	Right superior temporal (posterior part)	0.0010
	6	Right lateral occipital	0.0013
	7	Left superior frontal sulcus (middle part)	0.0156
SD	1	Right superior temporal (anterior part)	0.0001<
	2	Left superior temporal	0.0001<
	3	Left superior frontal (anterior part)	0.0039
**PT > FT**			
LGI	1	Left isthmus cingulate	0.0013

Cortical measurement index, cluster number per contrast (in ascending order of correct p-value), regions of cluster localization, and corrected p-values. For group differences, the direction of contrast FT > PT indicates that cortical measurement in the full-term infants is greater than that in the preterm infants and vice versa. Abbreviations: PNA, postnatal age; FT, full term; PT, preterm; LGI, local gyrification index; SD, sulcal depth.

For Review Only

Table 2. Differences in Cortical Measurements in the Preterm Subgroup.

Index	Cluster	Region	Adj. P-value
**LP > E-VP**			
LGI	1	Right superior temporal (anterior part)	0.0001<
	2	Left lingual	0.0279
SD	1	Right superior temporal (anterior part)	0.0004

Cortical measurement index, cluster number per contrast (in ascending order of corrected p-value), regions of cluster localization, and corrected p-values. For subgroup differences, the direction of contrast LP > E-VP indicates that the cortical measurement in the LP is greater than that in the E-VP, and vice versa. Abbreviations: PNA, postnatal age; LP, late preterm; E-VP, extremely preterm; LGI, local gyrification index; SD, sulcal depth.

For Review Only

Table 3. Group Differences in the Associations Between Cortical Measures and WPPSI-IV Subsets.

Region	WPPSI Subset	Index	β (group x measures)	p-value	FDR p-value
Right superior temporal	VCI	LGI	-11.53	0.352	0.822
		SD	-18.48	0.005*	0.014*
	WMI	LGI	-32.47	0.032*	0.129
		SD	-20.62	0.009*	0.014*
	FSIQ	LGI	-19.72	0.126	0.543
		SD	-17.65	0.010*	0.031*
Left superior temporal	WMI	LGI	-19.95	0.037*	0.129
		SD	-23.51	0.001*	0.004*

Full results are provided in Supplementary Table S2 and S3. Abbreviations: WPPSI, Wechsler Preschool and Primary Scale of Intelligence; FT, full-term; PT, preterm; LGI, local gyrification index; SD, sulcal depth; VCI, verbal comprehension index; WMI, working memory index; FSIQ, Full-Scale IQ; FDR, false positive discovery. * indicates P < 0.05.

For Review Only

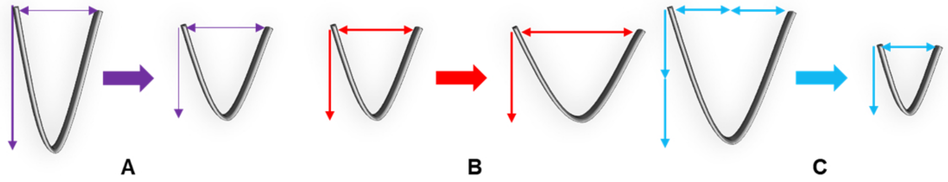

Figure 1. Influence of sulcal width and depth on LGI. Variations in LGI can result from changes in sulcal depth, width, or their combination. To analyze different scenarios, consider three characteristics: A. half depth (both LGI and SD decreases); B. double width (only LGI decreases); C. half width and depth (only SD decreases, and LGI does not necessarily decrease). Thus, sulcal depth and LGI need to be considered together to explicitly explain cortical folding differences. Abbreviations: LGI, local gyrification index.

100x23mm (300 x 300 DPI)

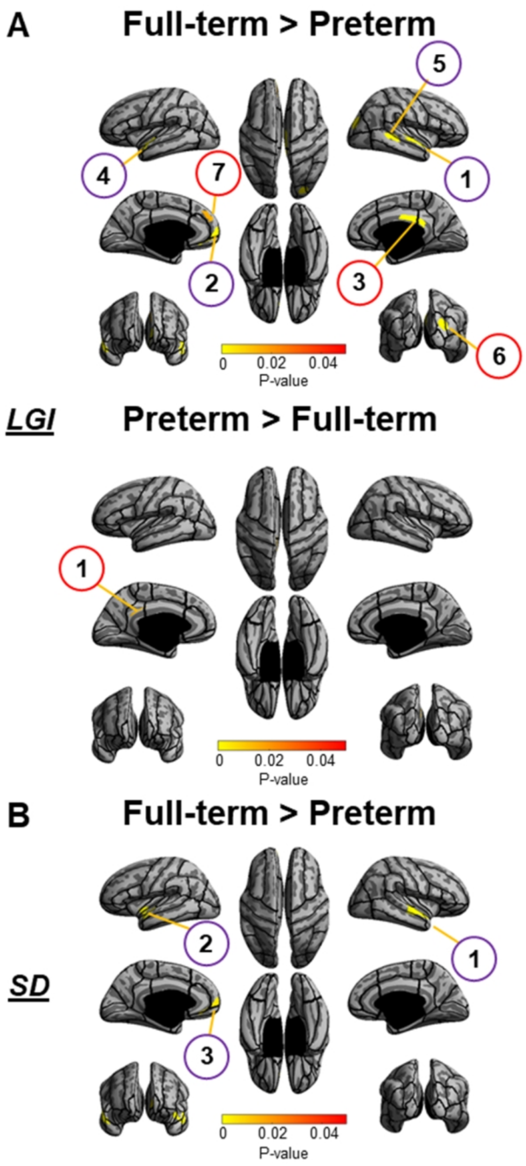

Figure 2. Preterm and full-term group differences in cortical measurements. Regions of statistically significant group differences in LGI (A) and SD (B) are shown, colored according to cluster corrected p-value (bottom scale). 1st and 3rd row indicate regions of lesser LGI and SD in the preterm group than in the full-term group, and vice versa in the 2nd row, at the P < 0.05 level after correcting for multiple comparisons via random field theory. Clusters were highlighted with a purple circle when both LGI and SD are significant and highlighted with a red circle when only LGI is significant. The scatter plot displays individual data points with fitted regression lines for each group, and the corresponding statistical test results (t-value, group sizes, p-value, FDR-corrected p-value, and interaction p-value) are reported within the figure. Abbreviations: FT, full-term; PT, preterm; LGI, local gyrification index; SD, sulcal depth.

79x166mm (300 x 300 DPI)

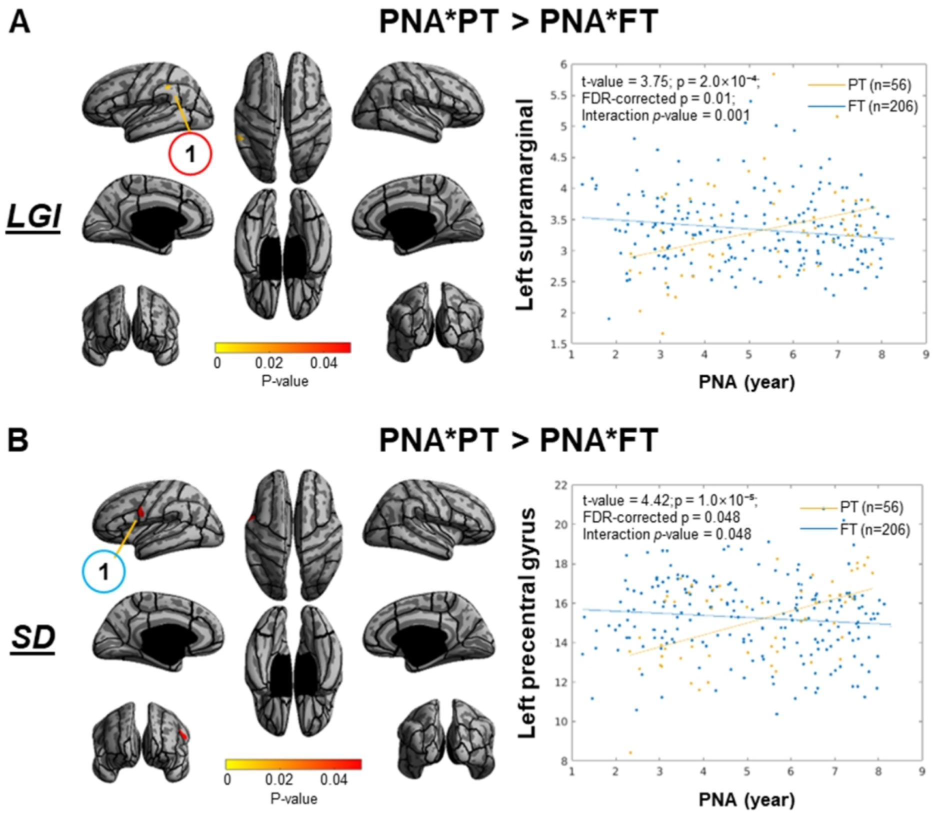

Figure 3. Statistically significant regions of PNA by group interactions by cortical measurements. Regions of statistically significant PNA by group differences in LGI (A) and SD (B) are shown, colored according to cluster p-value (Bottom scale). First row indicates regions of lesser slope with PNA in the preterm group than in the full-term group and vice versa in the remaining two rows, at the $P < 0.05$ level after correcting for multiple comparisons via random field theory. Clusters were highlighted with a red circle when only LGI is significant and highlighted with a blue circle when only SD is significant. Abbreviations: PNA, postnatal age; FT, full-term; PT, preterm; LGI, local gyrification index; SD, sulcal depth.

136x115mm (300 x 300 DPI)

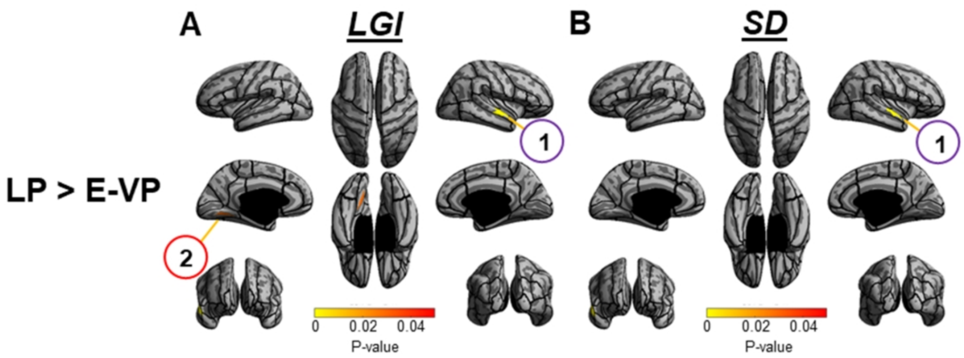

Figure 4. Preterm subgroup differences in cortical measurements. Regions of statistically significant group differences in LGI (A) and SD (B) are shown, colored according to cluster corrected p-value (bottom scale). The results indicate regions of lesser LGI and SD in the E-VP group than in the LP group, at the P < 0.05 level after correcting for multiple comparisons via random field theory. Clusters were highlighted with a purple circle when both LGI and SD are significant and highlighted with a red circle when only LGI is significant. The scatter plot displays individual data points with fitted regression lines for each group, and the corresponding statistical test results (t-value, group sizes, p-value, FDR-corrected p-value, and interaction p-value) are reported within the figure. Abbreviations: LP, late preterm; E-VP, extremely-to-very preterm; LGI, local gyrification index; SD, sulcal depth.

173x67mm (300 x 300 DPI)

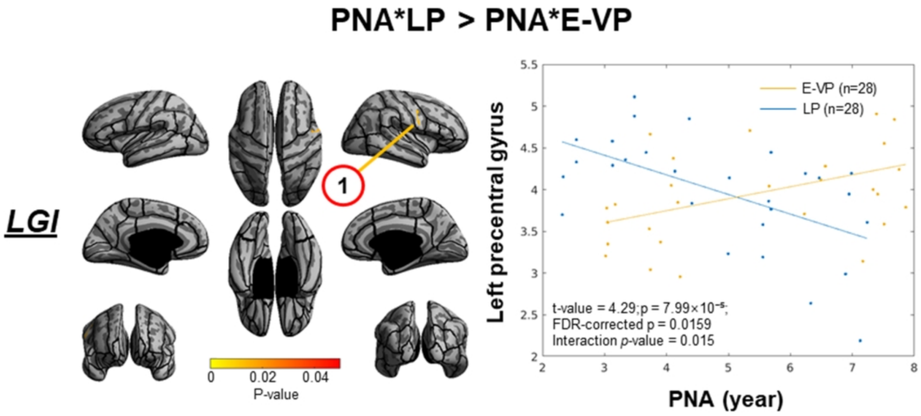

Figure 5. Statistically significant regions of PNA by preterm subgroup interactions by cortical measurements. Regions of statistically significant PNA by group differences in LGI. The results indicate regions where the slope with PNA is less in the E-VP group than in the LP group for LGI, at the $P < 0.05$ level after correcting for multiple comparisons via random field theory. The cluster was highlighted with a red circle when only LGI is significant. Abbreviations: PNA, postnatal age; LP, late preterm; E-VP, extremely-to-very preterm; LGI, local gyrification index.

162x74mm (300 x 300 DPI)

Supplementary Materials

Text S1. Quality assessments protocol

The initial step involved a visual inspection of the raw imaging data to classify issues such as motion artifacts, ghosting, and ringing into three categories: good, moderate, and poor[1-3]. Data classified as "poor" were excluded from further analysis. Given the rapid and extensive brain development that occurs in preschool-aged children, particular care must be taken to analyze contrast imaging and brain morphology in this population[4]. In this study, the remaining images were subjected to visual assessments using Quality Assurance (QA) tools and ENIGMA (Enhancing Neuro Imaging Genetics through Meta-Analysis) algorithms, which provided both quantitative and qualitative information about image quality. The automated assessments were conducted in two key steps: First, subcortical segmentation of regional volumes was analyzed using QA tools to identify individual-level outliers within the dataset. Second, cortical surface segmentation was evaluated by plotting and combining snapshots of inner and outer slices through ENIGMA algorithms, offering qualitative insights. Errors in brain segmentation can occur due to inaccuracies in the normalization of WM intensity in children. To address this, control points were manually adjusted to regulate WM hypointensities, ensuring they remained within a range of 80–110[5]. Finally, to ensure precision, two independent researchers performed a visual review of the reanalyzed images, selecting the final dataset for inclusion.

For Review Only

Text S2. Validation of linear age modeling

To ensure that the linear modeling of age adequately captures developmental trajectories within our cohort, we conducted an empirical validation of the age effect model. During early infancy (0-2 years), cortical growth has been shown to follow highly nonlinear trajectories, often characterized by logarithmic or quadratic patterns. [6,7] However, our dataset primarily includes children aged 1-7 years, a developmental window following the rapid postnatal expansion phase. Prior studies have reported that cortical folding measures already exhibit approximately linear age-related changes even within the 0-2 year period. [8,9] Based on these findings, we adopted a linear age term as an appropriate model for cortical development in our age range.

To verify this assumption, we performed an F-test for nested linear models using SurfStat to assess whether the inclusion of higher-order age terms improved the model fit beyond the linear specification. We test the following linear model (example):

$$measure1 = \beta_0 + \beta_1(PT/FT) + \beta_2 PNA + \beta_3 sex + u_{0j} + \epsilon \; (1)$$

$$measure2 = measure1 + \beta_4 PNA^2 \; (2)$$

This analysis was applied to both the FT–PT and E-VP–LP group comparisons, as well as their interaction terms. The results showed that the quadratic term did not yield any significant clusters after multiple comparison correction. These findings indicate that within our 1-7 year-old sample, cortical folding variations are sufficiently described by a linear age effect, with no additional explanatory benefit from higher-order terms. This supports previous evidence suggesting that cortical maturation during mid-to-late childhood follows an approximately linear developmental trajectory[7]

For Review Only

Table S1. Clinical characteristics

**Variables**	**Preterm (n = 56)**	**Full-term (n = 206)**	***p values***
Gestational age, weeks	31.43 ± 3.89	38.88 ± 1.80	<0.001
Postnatal age, years	4.61 ± 1.57	4.36 ± 1.77	0.438
Male, n (%)	40 (71.4%)	123 (59.7%)	0.147
Maternal education, n (%)	**Preterm (n = 22)**	**Full-term (n = 22)**	
<12 years	5 (22.7%)	3 (13.6%)	0.696
<16 years	15 (68.2%)	16 (72.7%)	1
>16 years	2 (9.1%)	3 (13.6%)	1
Follow-up characteristics			
WPPSI-IV scores	**Preterm (n = 22)**	**Full-term (n = 22)**	
VCI	80.23 ± 24.24	94.41 ± 12.93	0.009
VSI	83.95 ± 20.08	101.77 ± 16.18	0.002
FRI	81.17 ± 21.15	100.62 ± 17.30	0.017
WMI	82.91 ± 23.97	99.65 ± 15.03	0.011
PSI	80.27 ± 22.73	87.91 ± 18.81	0.401
FSIQ	76.45 ± 23.32	98.14 ± 15.39	<0.001

For Review Only

Abbreviations: WPPSI, Wechsler Preschool and Primary Scale of Intelligence; FT, full-term; PT, preterm; WMI, working memory index; VCI, verbal comprehension index; FRI, fluid reasoning index; VSI, visual spatial index; PSI, processing speed index, FSIQ, full scale intelligence quotient.

For Review Only

Table S2. Group difference in correlation strength between LGI and WPPSI-IV subset.

Index	Region	WPPSI Subset	β(group x measure	p-value	FDR p-value
LGI	Right superior temporal (anterior part)	VCI	−11.53	0.352	0.822
	Left superior frontal (anterior part)	VCI	6.52	0.704	0.822
	Right posterior cingulate, isthmus cingulate	VCI	−17.80	0.176	0.822
	Left superior temporal	VCI	−7.43	0.388	0.822
	Right superior temporal (posterior part)	VCI	2.68	0.605	0.822
	Right lateral occipital	VCI	2.47	0.672	0.822
	Left superior frontal sulcus (middle part)	VCI	−0.60	0.963	0.963
	Right superior temporal (anterior part)	VSI	3.92	0.760	0.956
	Left superior frontal (anterior part)	VSI	3.88	0.820	0.956
	Right posterior cingulate, isthmus cingulate	VSI	12.10	0.368	0.839
	Left superior temporal	VSI	−5.98	0.480	0.839
	Right superior temporal (posterior part)	VSI	0.34	0.995	0.995
	Right lateral occipital	VSI	0.04	0.995	0.995
	Left superior frontal sulcus (middle part)	VSI	−23.25	0.069	0.240
	Right superior temporal (anterior part)	FRI	−17.98	0.359	0.502
	Left superior frontal (anterior part)	FRI	−38.84	0.133	0.447
	Right posterior cingulate, isthmus cingulate	FRI	−30.19	0.192	0.447
	Left superior temporal	FRI	−14.21	0.337	0.447
	Right superior temporal (posterior part)	FRI	5.12	0.565	0.660
	Right lateral occipital	FRI	−0.82	0.913	0.913
	Left superior frontal sulcus (middle part)	FRI	−26.01	0.081	0.447
	Right superior temporal (anterior part)	WMI	−32.47	0.032	0.129
	Left superior frontal (anterior part)	WMI	27.29	0.168	0.393
	Right posterior cingulate, isthmus cingulate	WMI	1.89	0.893	0.946
	Left superior temporal	WMI	−19.95	0.037	0.129
	Right superior temporal (posterior part)	WMI	4.64	0.513	0.898
	Right lateral occipital	WMI	−2.43	0.725	0.946
	Left superior frontal sulcus (middle part)	WMI	0.95	0.946	0.946

For Review Only

Right superior temporal (anterior part)	PSI	−32.48	0.236	0.276
Left superior frontal (anterior part)	PSI	−58.84	0.024	0.170
Right posterior cingulate, isthmus cingulate	PSI	−17.78	0.351	0.351
Left superior temporal	PSI	−24.67	0.135	0.236
Right superior temporal (posterior part)	PSI	−13.33	0.222	0.276
Right lateral occipital	PSI	4.47	0.623	0.623
Left superior frontal sulcus (middle part)	PSI	−24.67	0.135	0.236
Right superior temporal (anterior part)	FSIQ	−19.72	0.126	0.543
Left superior frontal (anterior part)	FSIQ	10.69	0.550	0.927
Right posterior cingulate, isthmus cingulate	FSIQ	0.27	0.984	0.984
Left superior temporal	FSIQ	−12.33	0.155	0.543
Right superior temporal (posterior part)	FSIQ	4.72	0.402	0.927
Right lateral occipital	FSIQ	1.62	0.794	0.927
Left superior frontal sulcus (middle part)	FSIQ	−4.00	0.769	0.927

Abbreviations: LGI, local gyrification index; WPPSI, Wechsler Preschool and Primary Scale of Intelligence; FT, full-term; PT, preterm; WMI, working memory index; VCI, verbal comprehension index; FRI, fluid reasoning index; VSI, visual spatial index; PSI, processing speed index, FSIQ, full scale intelligence quotient.

For Review Only

Table S3. Group difference in correlation strength between SD and WPPSI-IV subset.

Index	Region	WPPSI Subset	β(group x measure	p-value	FDR p-value
SD	Right superior temporal (anterior part)	VCI	−18.48	0.005	0.014
	Left superior temporal	VCI	−9.56	0.115	0.173
	Left superior frontal (anterior part)	VCI	−14.32	0.120	0.173
	Right superior temporal (anterior part)	VSI	−10.96	0.114	0.341
	Left superior temporal	VSI	−6.41	0.313	0.469
	Left superior frontal (anterior part)	VSI	−7.52	0.324	0.469
	Right superior temporal (anterior part)	FRI	−14.32	0.120	0.180
	Left superior temporal	FRI	−16.61	0.047	0.140
	Left superior frontal (anterior part)	FRI	−5.91	0.541	0.541
	Right superior temporal (anterior part)	WMI	−20.62	0.009	0.014
	Left superior temporal	WMI	−23.51	0.001	0.004
	Left superior frontal (anterior part)	WMI	−5.73	0.492	0.738
	Right superior temporal (anterior part)	PSI	−21.56	0.087	0.087
	Left superior temporal	PSI	−23.02	0.022	0.067
	Left superior frontal (anterior part)	PSI	−6.34	0.450	0.675
	Right superior temporal (anterior part)	FSIQ	−17.65	0.010	0.031
	Left superior temporal	FSIQ	−9.34	0.130	0.194
	Left superior frontal (anterior part)	FSIQ	−2.16	0.837	0.837

Abbreviations: SD, sulcal depth; WPPSI, Wechsler Preschool and Primary Scale of Intelligence; FT, full-term; PT, preterm; WMI, working memory index; VCI, verbal comprehension index; FRI, fluid reasoning index; VSI, visual spatial index; PSI, processing speed index, FSIQ, full scale intelligence quotient.

For Review Only

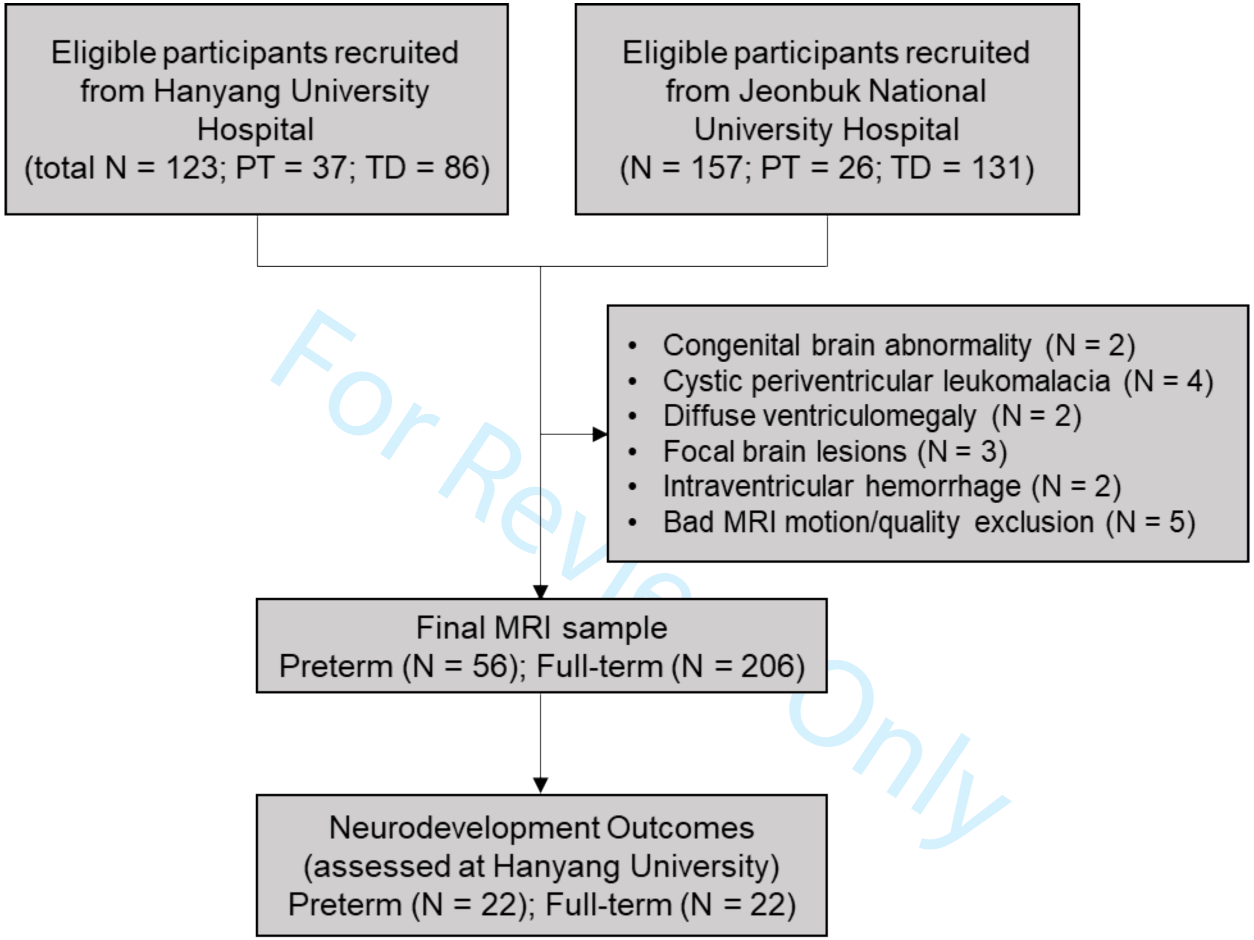

Figure S1. Flow diagram of participant inclusion and outcome availability.

For Review Only

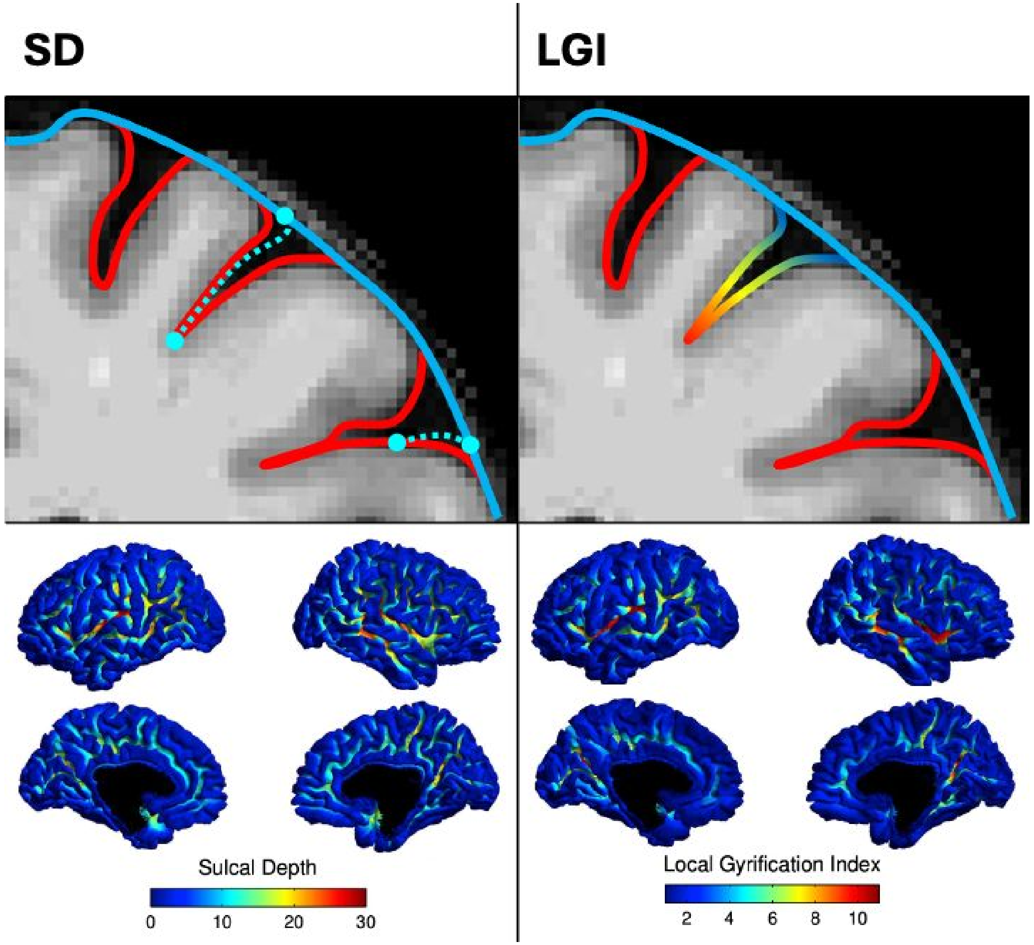

Figure S2. Cortical measurements on an example cortical section (top) and examples of the full feature maps (bottom). For *SD*, the green bars indicate the measured geodesic distance between vertices. For *SD* and *LGI*, the red contour indicates the pial surface and the blue contour indicates the cerebral hull. *LGI* is then defined as the ratio of the (red surface area)/(blue surface are), using a shape-adaptive local kernel. The

gradients for *LGI* and the bottom figures signify where magnitude is expected to be greater (red) and lower (blue). Abbreviations: SD, sulcal depth; LGI, local gyrification index.

For Review Only

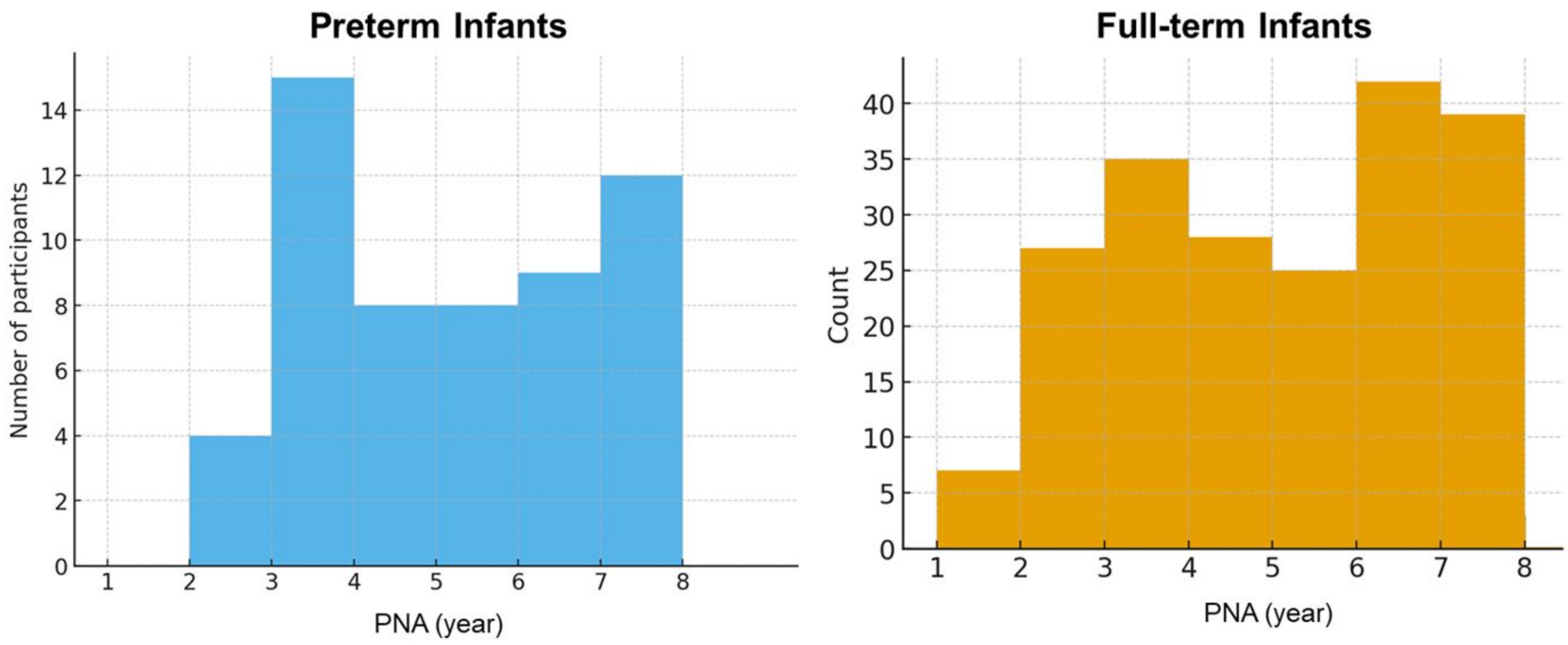

Figure S3. Postnatal age distribution in preterm and full-term groups. Abbreviation: PNA, postnatal age

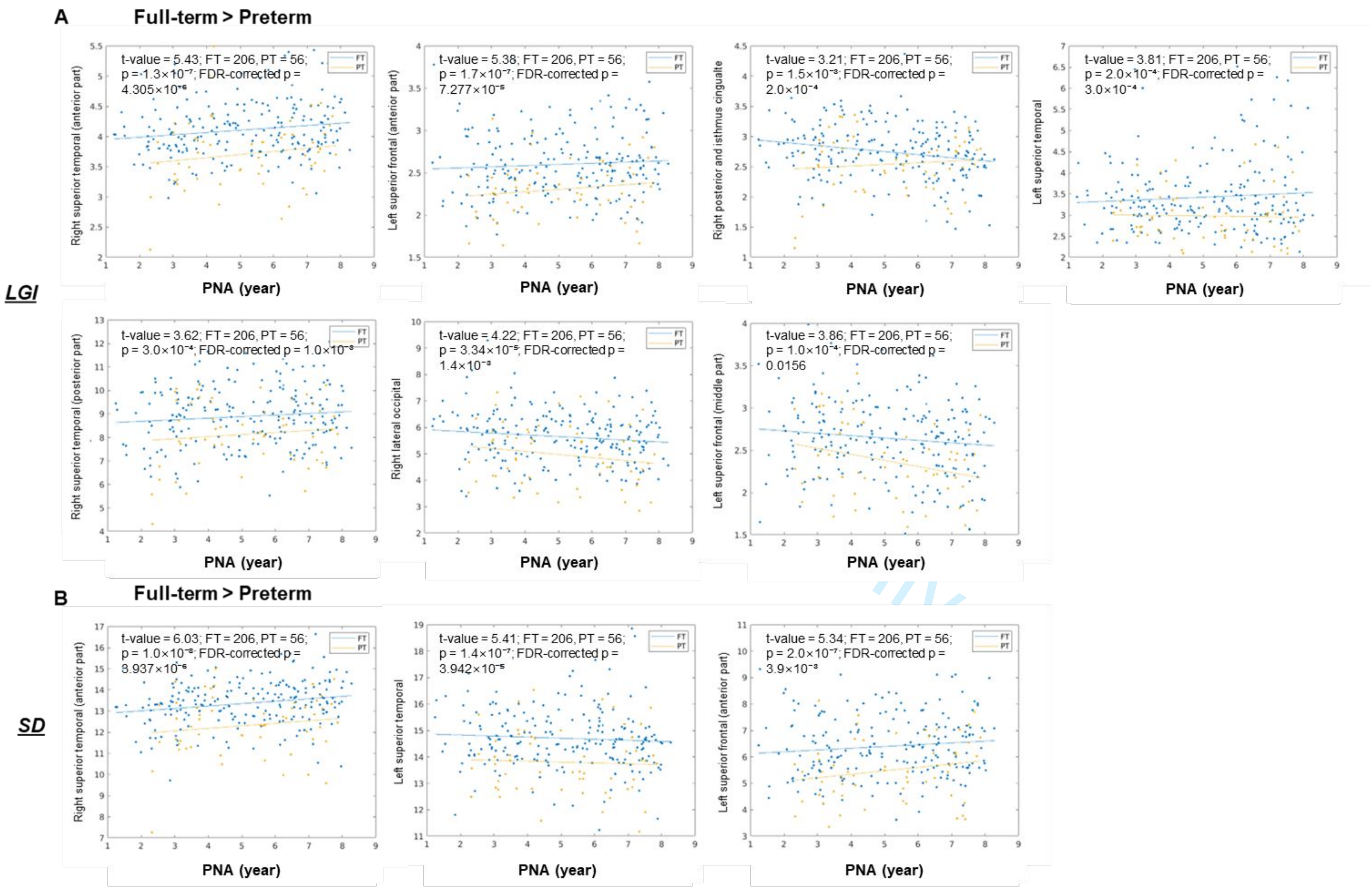
A
Full-term > Preterm
LGI
t-value = 5.43; FT = 206, PT = 56; p = 1.3×10⁻⁷; FDR-corrected p = 4.305×10⁻⁶
Right superior temporal (anterior part)
t-value = 5.38; FT = 206, PT = 56; p = 1.7×10⁻⁷; FDR-corrected p = 7.277×10⁻⁵
Left superior frontal (anterior part)
t-value = 3.21; FT = 206, PT = 56; p = 1.5×10⁻³; FDR-corrected p = 2.0×10⁻⁴
Right posterior and isthmus cingulate
t-value = 3.81; FT = 206, PT = 56; p = 2.0×10⁻⁴; FDR-corrected p = 3.0×10⁻⁴
Left superior temporal
t-value = 3.62; FT = 206, PT = 56; p = 3.0×10⁻⁴; FDR-corrected p = 1.0×10⁻³
Right superior temporal (posterior part)
t-value = 4.22; FT = 206, PT = 56; p = 3.34×10⁻⁵; FDR-corrected p = 1.4×10⁻³
Right lateral occipital
t-value = 3.86; FT = 206, PT = 56; p = 1.0×10⁻⁴; FDR-corrected p = 0.0156
Left superior frontal (middle part)
B
Full-term > Preterm
SD
t-value = 6.03; FT = 206, PT = 56; p = 1.0×10⁻⁸; FDR-corrected p = 3.937×10⁻⁶
Right superior temporal (anterior part)
t-value = 5.41; FT = 206, PT = 56; p = 1.4×10⁻⁷; FDR-corrected p = 3.942×10⁻⁵
Left superior temporal
t-value = 5.34; FT = 206, PT = 56; p = 2.0×10⁻⁷; FDR-corrected p = 3.9×10⁻³
Left superior frontal (anterior part)
FT
PT
PNA (year)

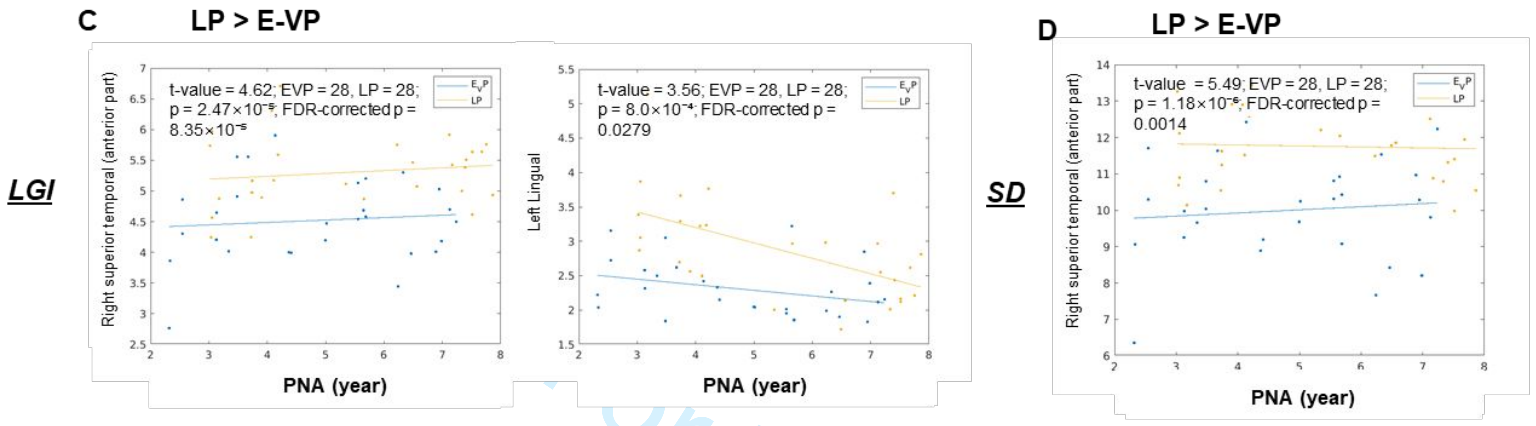

Figure S4. Scatterplot of all clusters in full-term > preterm contrast analysis (A and B) and LP > E-VP contrast analysis (C and D). **A**. Regions in which the local gyrification index was significantly lower in preterm than full-term children. **B.** Regions in which sulcal depth was significantly lower in preterm than full-term children. **C**. Regions in which the local gyrification index was significantly lower in E-VP than LP children. **D.** Regions in which sulcal depth was significantly lower in E-VP than LP children. In each scatter-plot, postnatal age (years) is plotted on the x-axis and the regional LGI or SD on the y-axis. Blue dots = individual full-term or E-VP cases; orange dots = individual preterm or LP cases. The scatter plot displays individual data points with fitted regression lines for each group, and the corresponding statistical test results (t-value, group sizes, p-value, FDR-corrected p-value, and interaction p-value) are reported within the figure. Abbreviations: FT, full-term; PT, preterm; E-VP, extremely-to-very preterm; LP, late preterm; LGI, local gyrification index; SD, sulcal depth; PNA, postnatal age.

For Review Only

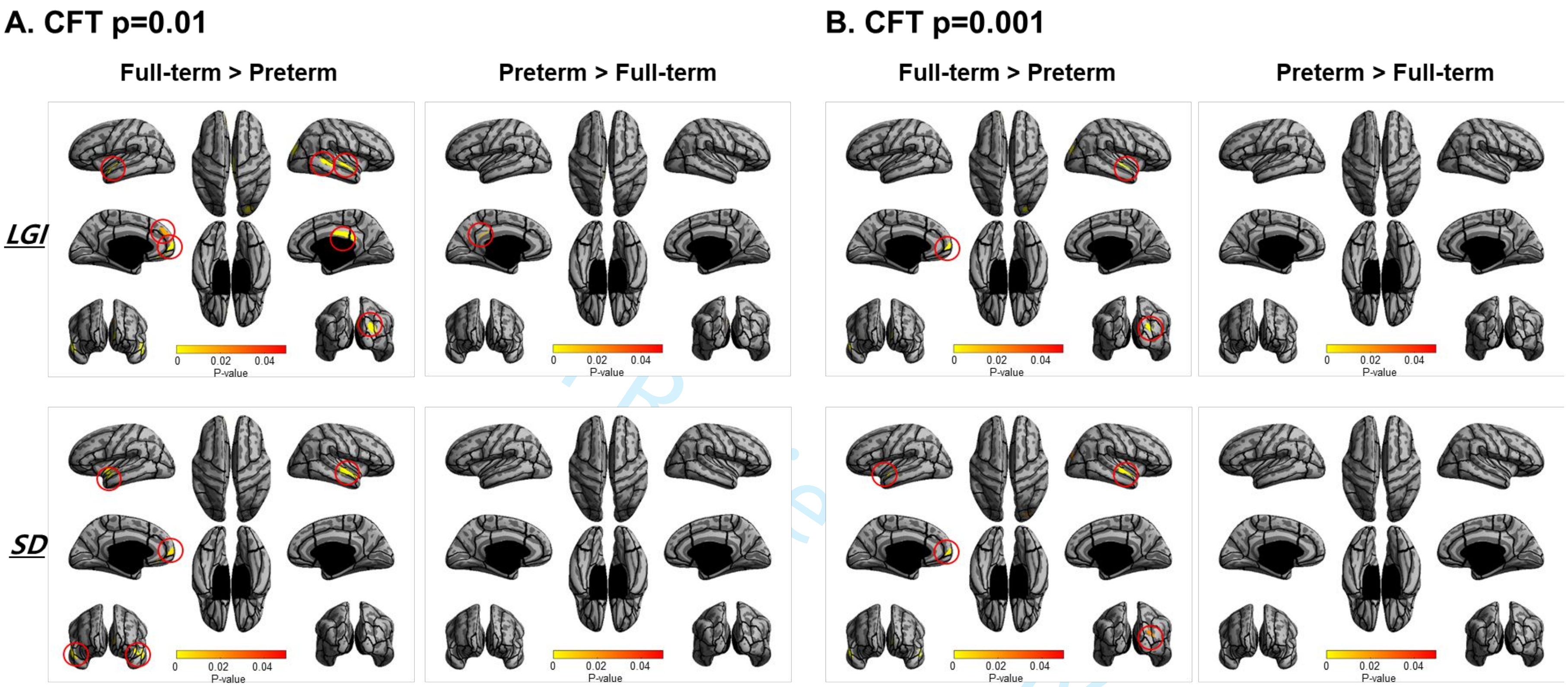

Figure S5. Preterm and full-term group differences in cortical measurements under two cluster-forming thresholds. Regions of statistically significant group differences in LGI and SD are shown for each cluster-forming threshold (CFT = 0.01 [A] and CFT = 0.001 [B]), colored according to the cluster-corrected p-value (bottom scale). Abbreviations: CFT, cluster-forming threshold; FT, full-term infants; PT, preterm infants; LGI, local gyrification index; SD, sulcal depth.

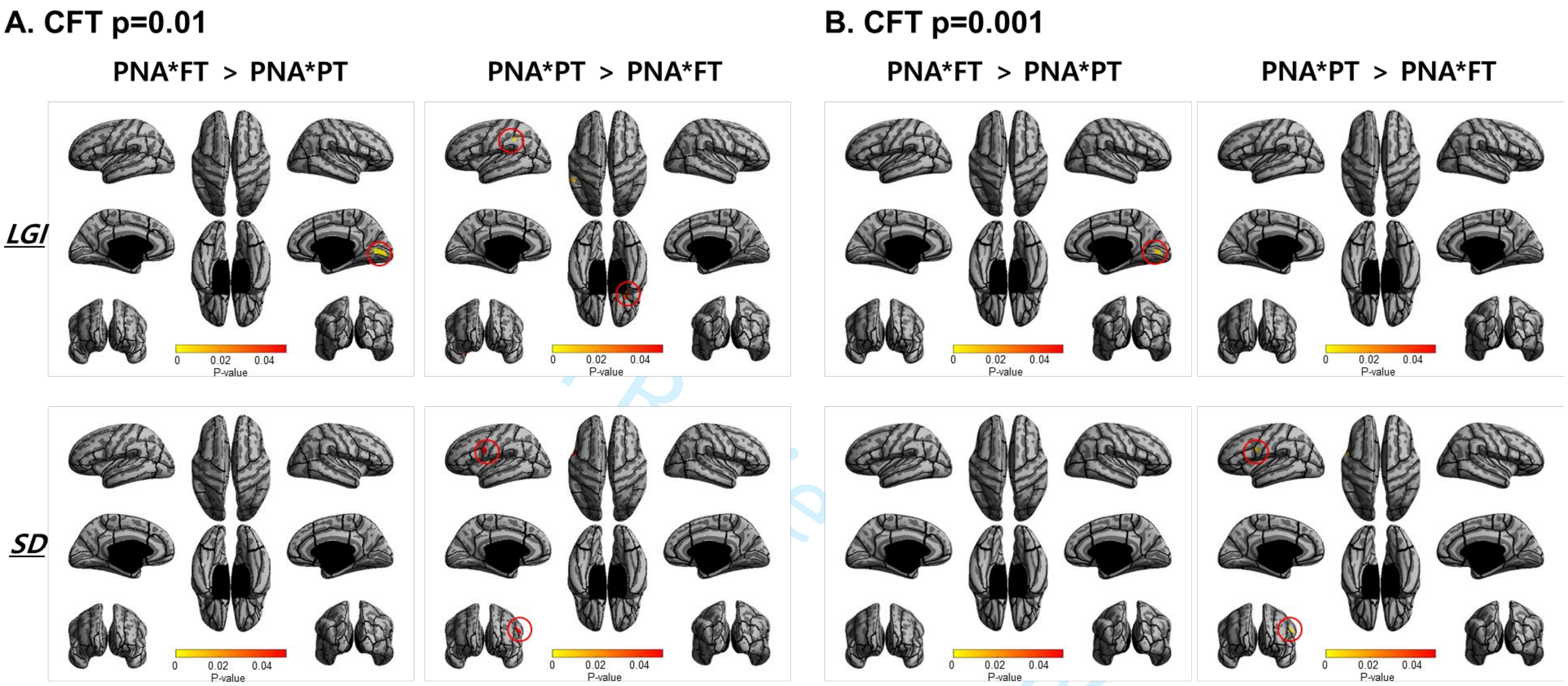

Figure S6. Statistically significant regions of PNA by group interactions by cortical measurements under two cluster-forming thresholds. Regions of statistically significant PNA by group differences in LGI and SD are shown for each cluster-forming threshold (CFT = 0.01 [A] and CFT = 0.001 [B]), colored according to the cluster-corrected p-value (bottom scale). Abbreviations: CFT, cluster-forming threshold; FT, full-term infants; PT, preterm infants; PNA, postnatal age; LGI, local gyrification index; SD, sulcal depth.

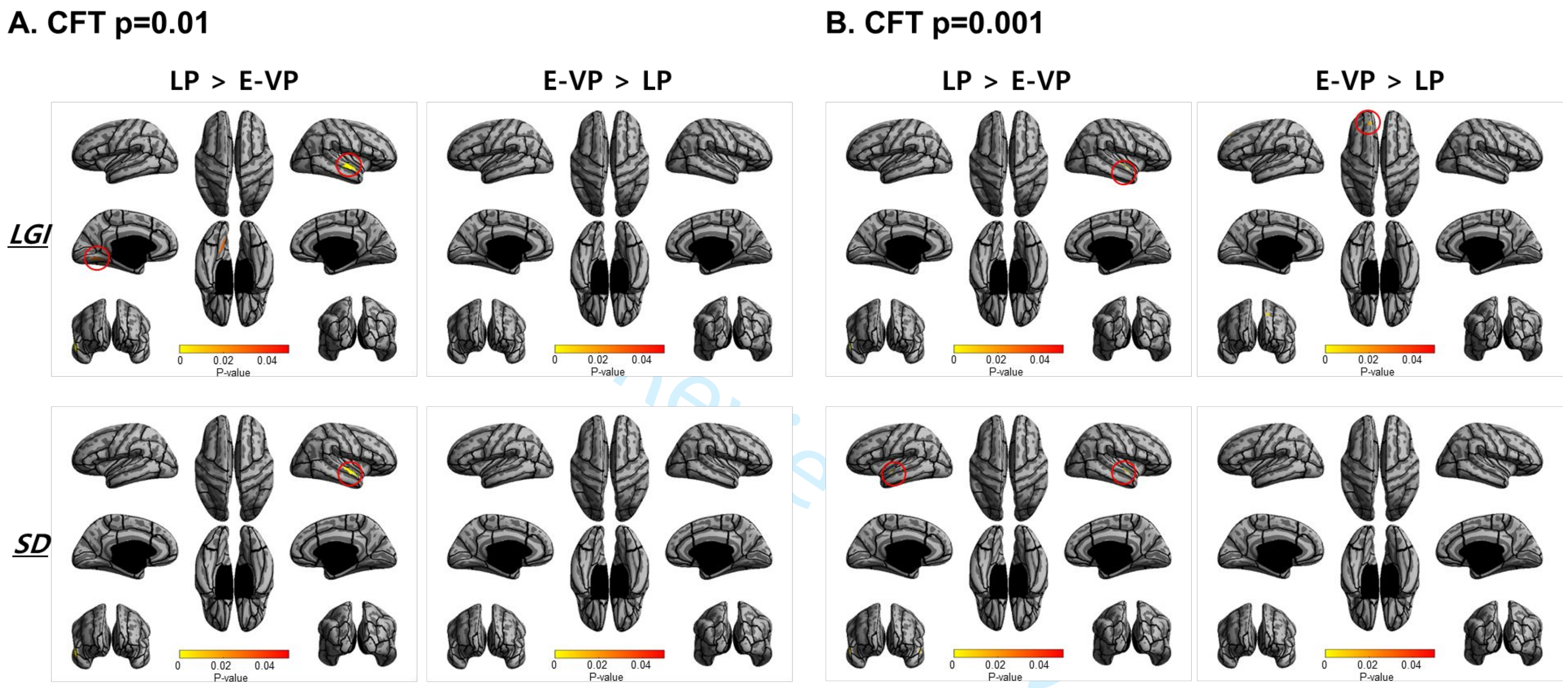

Figure S7. preterm subgroup differences in cortical measurements under two cluster-forming thresholds. Regions of statistically significant preterm subgroup differences in LGI and SD are shown for each cluster-forming threshold (CFT = 0.01 [A] and CFT = 0.001 [B]), colored according to the cluster-corrected p-value (bottom scale). Abbreviations: CFT, cluster-forming threshold; LP, late preterm; E-VP, extremely-to-very preterm; LGI, local gyrification index; SD, sulcal depth.

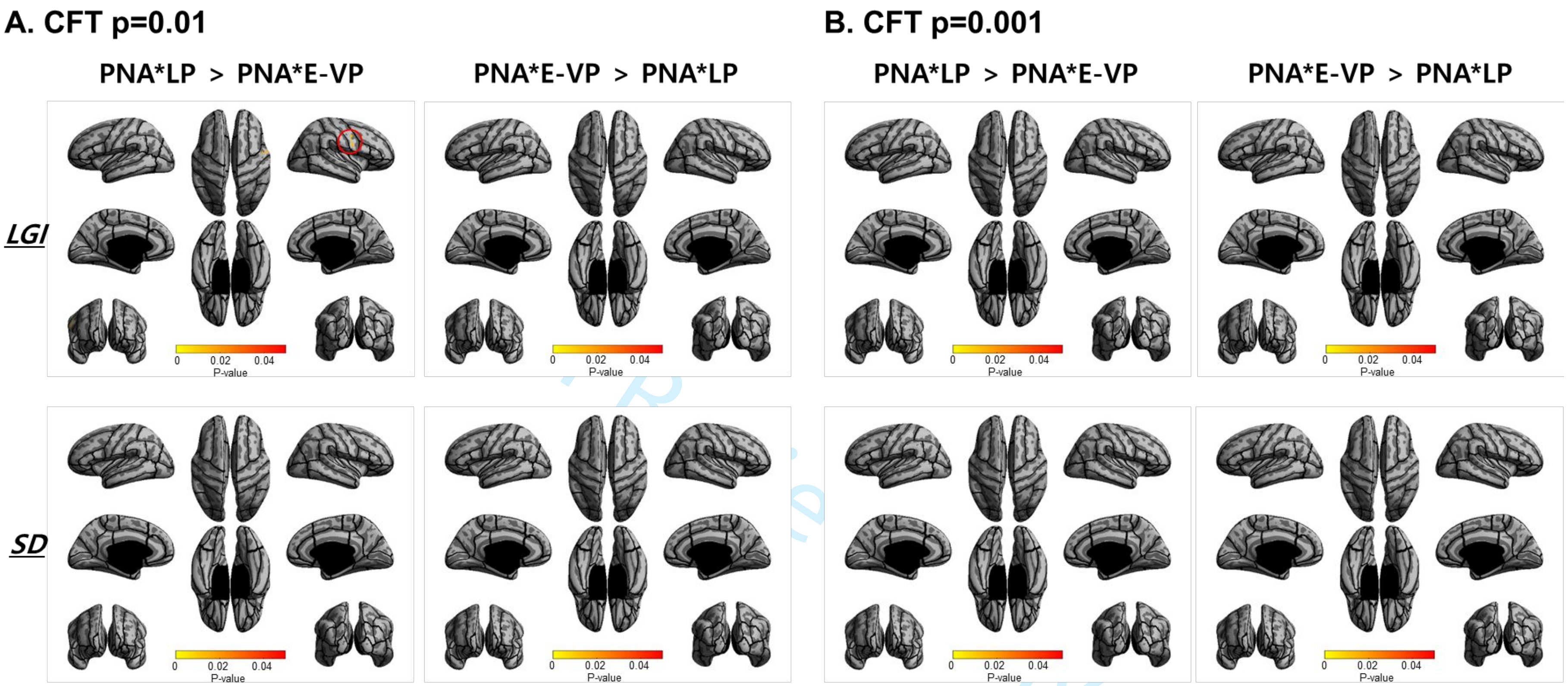

Figure S8. Statistically significant regions of PNA by preterm subgroup interactions by cortical measurements under two cluster-forming thresholds. Regions of statistically significant PNA by preterm subgroup differences in LGI and SD are shown for each cluster-forming threshold (CFT = 0.01 [A] and CFT = 0.001 [B]), colored according to the cluster-corrected p-value (bottom scale). Abbreviations: CFT, cluster-forming threshold; LP, late preterm; E-VP, extremely-to-very preterm; PNA, postnatal age; LGI, local gyrification index; SD, sulcal depth.

References

1. Blumenthal JD, Zijdenbos A, Molloy E, Giedd JN. Motion artifact in magnetic resonance imaging: implications for automated analysis. *Neuroimage*. May 2002;16(1):89-92. doi:10.1006/nimg.2002.1076

2. Shaw P, Eckstrand K, Sharp W*, et al*. Attention-deficit/hyperactivity disorder is characterized by a delay in cortical maturation. *Proc Natl Acad Sci U S A*. Dec 4 2007;104(49):19649-54. doi:10.1073/pnas.0707741104

3. Tisdall MD, Reuter M, Qureshi A, Buckner RL, Fischl B, van der Kouwe AJW. Prospective motion correction with volumetric navigators (vNavs) reduces the bias and variance in brain morphometry induced by subject motion. *Neuroimage*. Feb 15 2016;127:11-22. doi:10.1016/j.neuroimage.2015.11.054

4. Guadalupe T, Mathias SR, vanErp TGM*, et al*. Human subcortical brain asymmetries in 15,847 people worldwide reveal effects of age and sex. *Brain Imaging Behav*. Oct 2017;11(5):1497-1514. doi:10.1007/s11682-016-9629-z

5. Rentería ME. Cerebral asymmetry: a quantitative, multifactorial, and plastic brain phenotype. *Twin Res Hum Genet*. Jun 2012;15(3):401-13. doi:10.1017/thg.2012.13

6. Alex AM, Aguate F, Botteron K*, et al*. A global multicohort study to map subcortical brain development and cognition in infancy and early childhood. *Nature neuroscience*. 2024;27(1):176-186.

7. Remer J, Croteau-Chonka E, Dean DC*, et al*. Quantifying cortical development in typically developing toddlers and young children, 1–6 years of age. *NeuroImage*. 2017/06/01/ 2017;153:246-261. doi:https://doi.org/10.1016/j.neuroimage.2017.04.010

For Review Only

8. Biagioni E, Frisone M, Laroche S, *et al*. Maturation of cerebral electrical activity and development of cortical folding in young very preterm infants. *Clinical neurophysiology*. 2007;118(1):53-59.

9. Shimony JS, Smyser CD, Wideman G, *et al*. Comparison of cortical folding measures for evaluation of developing human brain. *Neuroimage*. 2016;125:780-790.

For Review Only
