# Supplementary material for: Long-term effects of preterm birth on cortical folding trajectories in early childhood
Source: Brain Commun. 2026 May 18;8(3):fcag097. doi: 10.1093/braincomms/fcag097 (PMC13181400; doi:10.1093/braincomms/fcag097)
Supplement: fcag097_Supplementary_Data [file fcag097_supplementary_data.zip › Supplementary_material.docx]

**Supplementary Materials**

**Supplementary Text 1.** Quality assessments protocol

The initial step involved a visual inspection of the raw imaging data to classify issues such as motion artifacts, ghosting, and ringing into three categories: good, moderate, and poor^1-3^. Data classified as "poor" were excluded from further analysis. Given the rapid and extensive brain development that occurs in preschool-aged children, particular care must be taken to analyze contrast imaging and brain morphology in this population^4^. In this study, the remaining images were subjected to visual assessments using Quality Assurance (QA) tools and ENIGMA (Enhancing Neuro Imaging Genetics through Meta-Analysis) algorithms, which provided both quantitative and qualitative information about image quality. The automated assessments were conducted in two key steps: First, subcortical segmentation of regional volumes was analyzed using QA tools to identify individual-level outliers within the dataset. Second, cortical surface segmentation was evaluated by plotting and combining snapshots of inner and outer slices through ENIGMA algorithms, offering qualitative insights. Errors in brain segmentation can occur due to inaccuracies in the normalization of WM intensity in children. To address this, control points were manually adjusted to regulate WM hypointensities, ensuring they remained within a range of 80–110^5^. Finally, to ensure precision, two independent researchers performed a visual review of the reanalyzed images, selecting the final dataset for inclusion.

**Supplementary Text 2.** Validation of linear age modeling

To ensure that the linear modeling of age adequately captures developmental trajectories within our cohort, we conducted an empirical validation of the age effect model. During early infancy (0-2 years), cortical growth has been shown to follow highly nonlinear trajectories, often characterized by logarithmic or quadratic patterns. ^6,7^ However, our dataset primarily includes children aged 1-7 years, a developmental window following the rapid postnatal expansion phase. Prior studies have reported that cortical folding measures already exhibit approximately linear age-related changes even within the 0-2 year period. ^8,9^ Based on these findings, we adopted a linear age term as an appropriate model for cortical development in our age range.

To verify this assumption, we performed an F-test for nested linear models using SurfStat to assess whether the inclusion of higher-order age terms improved the model fit beyond the linear specification. We test the following linear model (example):

$$measure1= \beta_{0}+\beta_{1}\left( PT/FT \right)+\beta_{2}PNA+\beta_{3}sex+u_{0j}+\epsilon(1)$$

$$measure2= measure1+\beta_{4}PNA^{2} (2)$$

This analysis was applied to both the FT–PT and E-VP–LP group comparisons, as well as their interaction terms. The results showed that the quadratic term did not yield any significant clusters after multiple comparison correction. These findings indicate that within our 1-7 year-old sample, cortical folding variations are sufficiently described by a linear age effect, with no additional explanatory benefit from higher-order terms. This supports previous evidence suggesting that cortical maturation during mid-to-late childhood follows an approximately linear developmental trajectory^7^

**Supplementary Table 1.** Clinical characteristics

| **Variables** | **Preterm (n = 56)** | **Full-term (n = 206)** | ***p values*** |
| --- | --- | --- | --- |
| Gestational age, weeks | 31.43 ± 3.89 | 38.88 ± 1.80 | <0.001 |
| Postnatal age, years | 4.61 ± 1.57 | 4.36 ± 1.77 | 0.438 |
| Male, n (%) | 40 (71.4%) | 123 (59.7%) | 0.147 |
| Maternal education, n (%) | **Preterm (n = 22)** | **Full-term (n = 22)** |  |
| <12 years | 5 (22.7%) | 3 (13.6%) | 0.696 |
| <16 years | 15 (68.2%) | 16 (72.7%) | 1 |
| >16 years | 2 (9.1%) | 3 (13.6%) | 1 |
| Follow-up characteristics | |  |  |
| WPPSI-IV scores | **Preterm (n = 22)** | **Full-term (n = 22)** |  |
| VCI | 80.23 ± 24.24 | 94.41 ± 12.93 | 0.009 |
| VSI | 83.95 ± 20.08 | 101.77 ± 16.18 | 0.002 |
| FRI | 81.17 ± 21.15 | 100.62 ± 17.30 | 0.017 |
| WMI | 82.91 ± 23.97 | 99.65 ± 15.03 | 0.011 |
| PSI | 80.27 ± 22.73 | 87.91 ± 18.81 | 0.401 |
| FSIQ | 76.45 ± 23.32 | 98.14 ± 15.39 | <0.001 |

Abbreviations: WPPSI, Wechsler Preschool and Primary Scale of Intelligence; FT, full-term; PT, preterm; WMI, working memory index; VCI, verbal comprehension index; FRI, fluid reasoning index; VSI, visual spatial index; PSI, processing speed index, FSIQ, full scale intelligence quotient.

**Supplementary Table 2.** Group difference in correlation strength between LGI and WPPSI-IV subset.

| **Index** | **Region** | **WPPSI Subset** | **β(group x measure** | **p-value** | **FDR p*-*value** |
| --- | --- | --- | --- | --- | --- |
| LGI | Right superior temporal (anterior part) | VCI | −11.53 | 0.352 | 0.822 |
|  | Left superior frontal (anterior part) | VCI | 6.52 | 0.704 | 0.822 |
|  | Right posterior cingulate, isthmus cingulate | VCI | −17.80 | 0.176 | 0.822 |
|  | Left superior temporal | VCI | −7.43 | 0.388 | 0.822 |
|  | Right superior temporal (posterior part) | VCI | 2.68 | 0.605 | 0.822 |
|  | Right lateral occipital | VCI | 2.47 | 0.672 | 0.822 |
|  | Left superior frontal sulcus (middle part) | VCI | −0.60 | 0.963 | 0.963 |
|  | Right superior temporal (anterior part) | VSI | 3.92 | 0.760 | 0.956 |
|  | Left superior frontal (anterior part) | VSI | 3.88 | 0.820 | 0.956 |
|  | Right posterior cingulate, isthmus cingulate | VSI | 12.10 | 0.368 | 0.839 |
|  | Left superior temporal | VSI | −5.98 | 0.480 | 0.839 |
|  | Right superior temporal (posterior part) | VSI | 0.34 | 0.995 | 0.995 |
|  | Right lateral occipital | VSI | 0.04 | 0.995 | 0.995 |
|  | Left superior frontal sulcus (middle part) | VSI | −23.25 | 0.069 | 0.240 |
|  | Right superior temporal (anterior part) | FRI | −17.98 | 0.359 | 0.502 |
|  | Left superior frontal (anterior part) | FRI | −38.84 | 0.133 | 0.447 |
|  | Right posterior cingulate, isthmus cingulate | FRI | −30.19 | 0.192 | 0.447 |
|  | Left superior temporal | FRI | −14.21 | 0.337 | 0.447 |
|  | Right superior temporal (posterior part) | FRI | 5.12 | 0.565 | 0.660 |
|  | Right lateral occipital | FRI | −0.82 | 0.913 | 0.913 |
|  | Left superior frontal sulcus (middle part) | FRI | −26.01 | 0.081 | 0.447 |
|  | Right superior temporal (anterior part) | WMI | −32.47 | 0.032 | 0.129 |
|  | Left superior frontal (anterior part) | WMI | 27.29 | 0.168 | 0.393 |
|  | Right posterior cingulate, isthmus cingulate | WMI | 1.89 | 0.893 | 0.946 |
|  | Left superior temporal | WMI | −19.95 | 0.037 | 0.129 |
|  | Right superior temporal (posterior part) | WMI | 4.64 | 0.513 | 0.898 |
|  | Right lateral occipital | WMI | −2.43 | 0.725 | 0.946 |
|  | Left superior frontal sulcus (middle part) | WMI | 0.95 | 0.946 | 0.946 |
|  | Right superior temporal (anterior part) | PSI | −32.48 | 0.236 | 0.276 |
|  | Left superior frontal (anterior part) | PSI | −58.84 | 0.024 | 0.170 |
|  | Right posterior cingulate, isthmus cingulate | PSI | −17.78 | 0.351 | 0.351 |
|  | Left superior temporal | PSI | −24.67 | 0.135 | 0.236 |
|  | Right superior temporal (posterior part) | PSI | −13.33 | 0.222 | 0.276 |
|  | Right lateral occipital | PSI | 4.47 | 0.623 | 0.623 |
|  | Left superior frontal sulcus (middle part) | PSI | −24.67 | 0.135 | 0.236 |
|  | Right superior temporal (anterior part) | FSIQ | −19.72 | 0.126 | 0.543 |
|  | Left superior frontal (anterior part) | FSIQ | 10.69 | 0.550 | 0.927 |
|  | Right posterior cingulate, isthmus cingulate | FSIQ | 0.27 | 0.984 | 0.984 |
|  | Left superior temporal | FSIQ | −12.33 | 0.155 | 0.543 |
|  | Right superior temporal (posterior part) | FSIQ | 4.72 | 0.402 | 0.927 |
|  | Right lateral occipital | FSIQ | 1.62 | 0.794 | 0.927 |
|  | Left superior frontal sulcus (middle part) | FSIQ | −4.00 | 0.769 | 0.927 |

Abbreviations: LGI, local gyrification index; WPPSI, Wechsler Preschool and Primary Scale of Intelligence; FT, full-term; PT, preterm; WMI, working memory index; VCI, verbal comprehension index; FRI, fluid reasoning index; VSI, visual spatial index; PSI, processing speed index, FSIQ, full scale intelligence quotient.

**Supplementary Table 3.** Group difference in correlation strength between SD and WPPSI-IV subset.

| **Index** | **Region** | **WPPSI Subset** | **β(group x measure** | **p-value** | **FDR p*-*value** |
| --- | --- | --- | --- | --- | --- |
| SD | Right superior temporal (anterior part) | VCI | −18.48 | 0.005 | 0.014 |
|  | Left superior temporal | VCI | −9.56 | 0.115 | 0.173 |
|  | Left superior frontal (anterior part) | VCI | −14.32 | 0.120 | 0.173 |
|  | Right superior temporal (anterior part) | VSI | −10.96 | 0.114 | 0.341 |
|  | Left superior temporal | VSI | −6.41 | 0.313 | 0.469 |
|  | Left superior frontal (anterior part) | VSI | −7.52 | 0.324 | 0.469 |
|  | Right superior temporal (anterior part) | FRI | −14.32 | 0.120 | 0.180 |
|  | Left superior temporal | FRI | −16.61 | 0.047 | 0.140 |
|  | Left superior frontal (anterior part) | FRI | −5.91 | 0.541 | 0.541 |
|  | Right superior temporal (anterior part) | WMI | −20.62 | 0.009 | 0.014 |
|  | Left superior temporal | WMI | −23.51 | 0.001 | 0.004 |
|  | Left superior frontal (anterior part) | WMI | −5.73 | 0.492 | 0.738 |
|  | Right superior temporal (anterior part) | PSI | −21.56 | 0.087 | 0.087 |
|  | Left superior temporal | PSI | −23.02 | 0.022 | 0.067 |
|  | Left superior frontal (anterior part) | PSI | −6.34 | 0.450 | 0.675 |
|  | Right superior temporal (anterior part) | FSIQ | −17.65 | 0.010 | 0.031 |
|  | Left superior temporal | FSIQ | −9.34 | 0.130 | 0.194 |
|  | Left superior frontal (anterior part) | FSIQ | −2.16 | 0.837 | 0.837 |

Abbreviations: SD, sulcal depth; WPPSI, Wechsler Preschool and Primary Scale of Intelligence; FT, full-term; PT, preterm; WMI, working memory index; VCI, verbal comprehension index; FRI, fluid reasoning index; VSI, visual spatial index; PSI, processing speed index, FSIQ, full scale intelligence quotient.


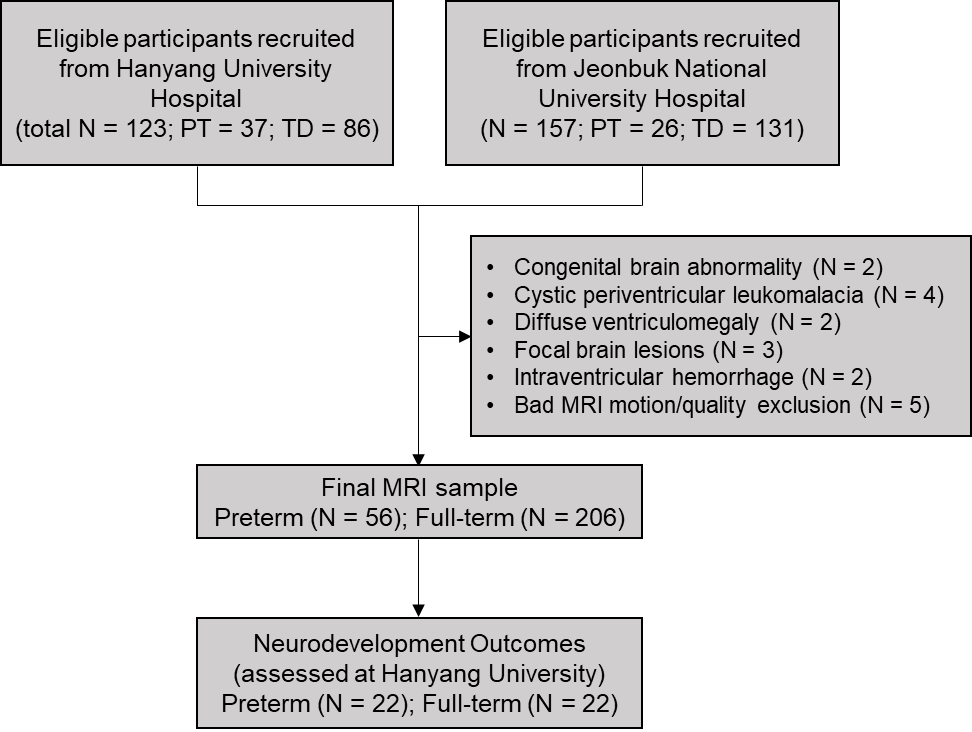


**Supplementary Figure 1.** Flow diagram of participant inclusion and outcome availability**.**

**
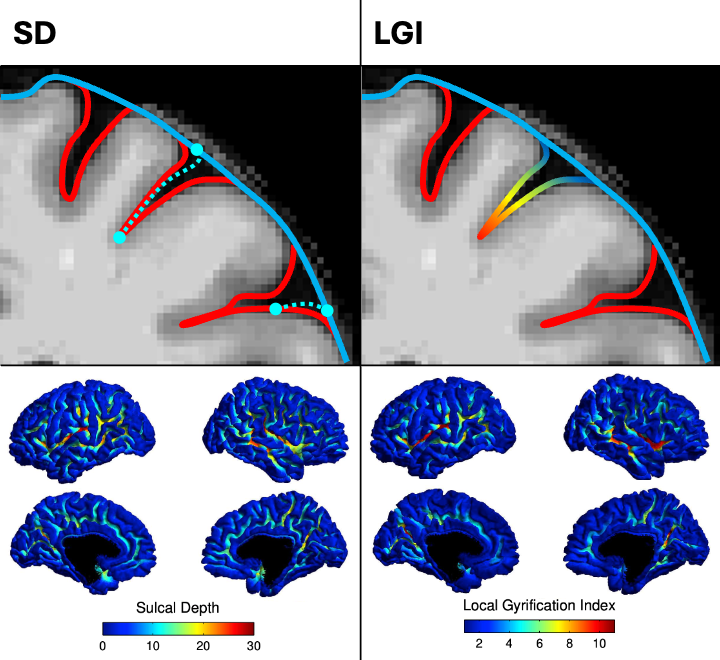
**

**Supplementary Figure 2.** Cortical measurements on an example cortical section (top) and examples of the full feature maps (bottom). For *SD*, the green bars indicate the measured geodesic distance between vertices. For *SD* and *LGI*, the red contour indicates the pial surface and the blue contour indicates the cerebral hull. *LGI* is then defined as the ratio of the (red surface area)/(blue surface are), using a shape-adaptive local kernel. The gradients for *LGI* and the bottom figures signify where magnitude is expected to be greater (red) and lower (blue). Abbreviations: SD, sulcal depth; LGI, local gyrification index.


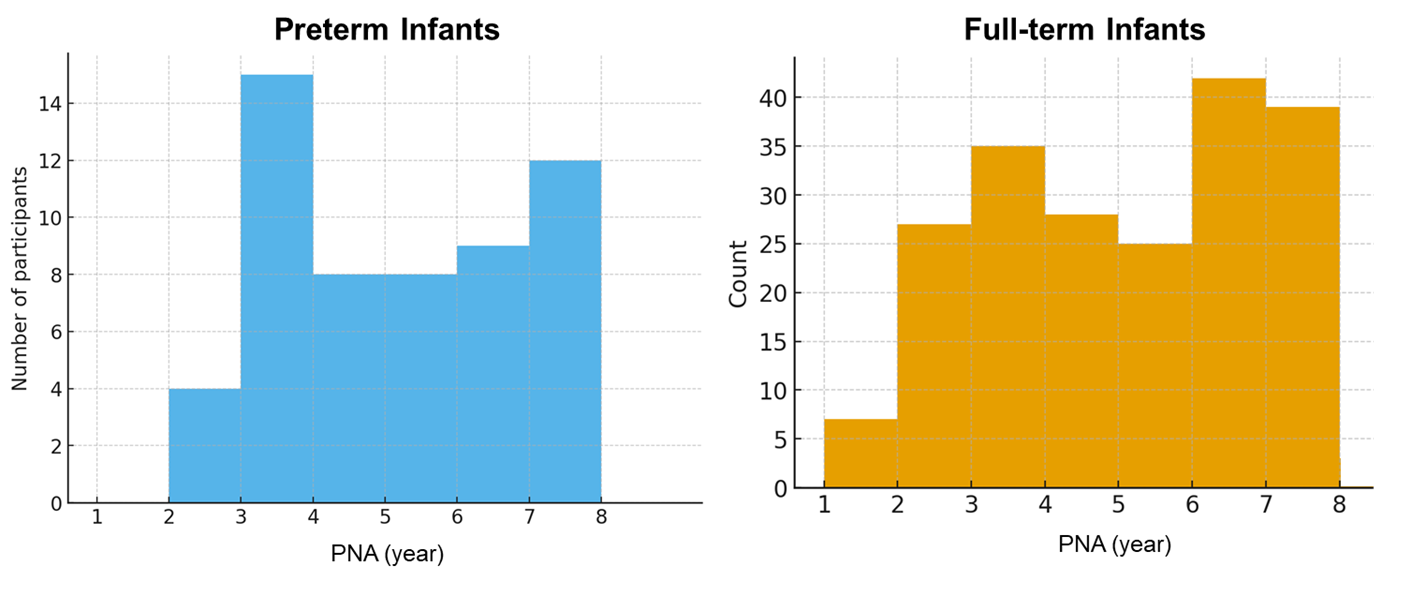


**Supplementary Figure 3.** Postnatal age distribution in preterm and full-term groups. Abbreviation: PNA, postnatal age

**
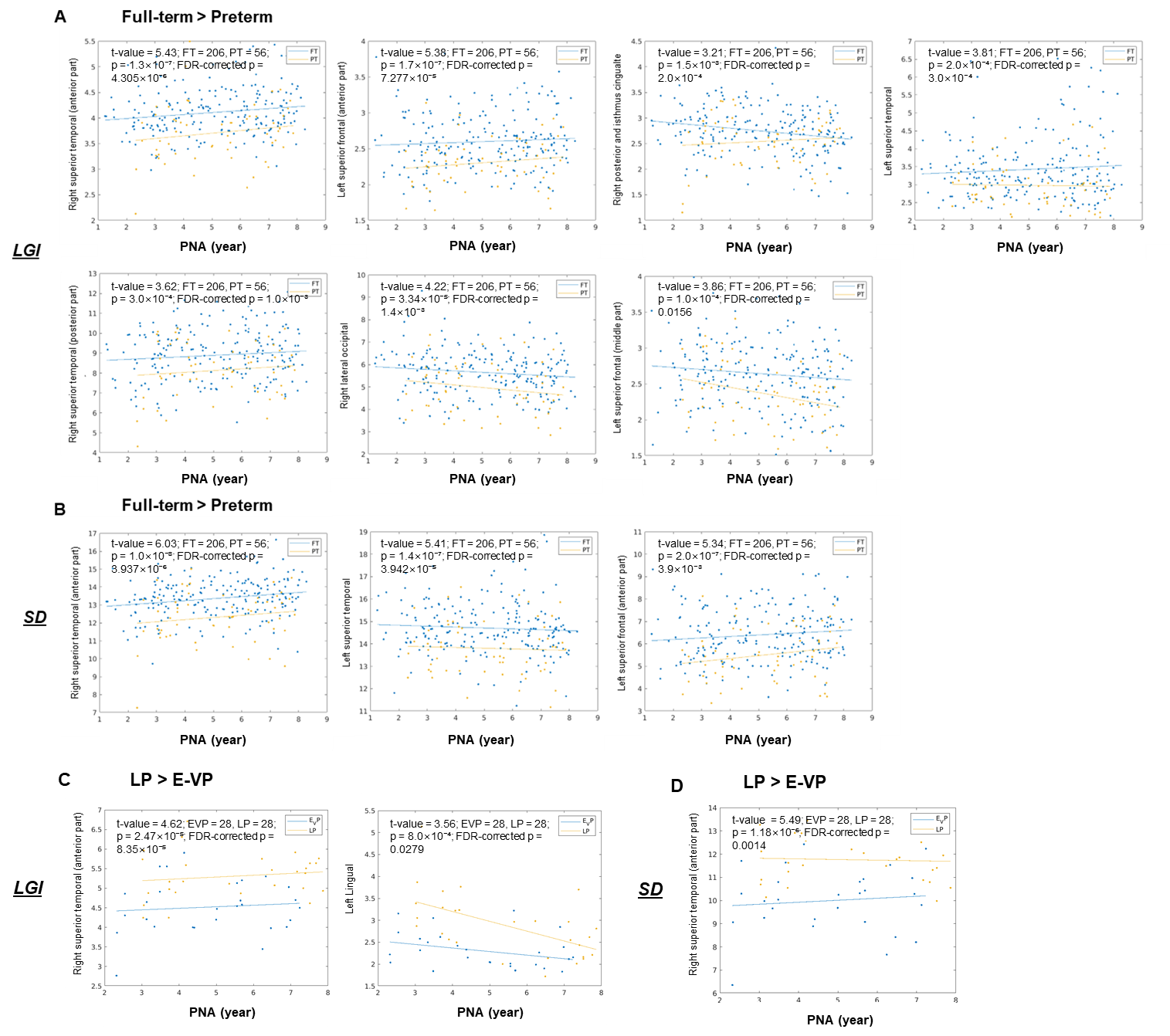
**


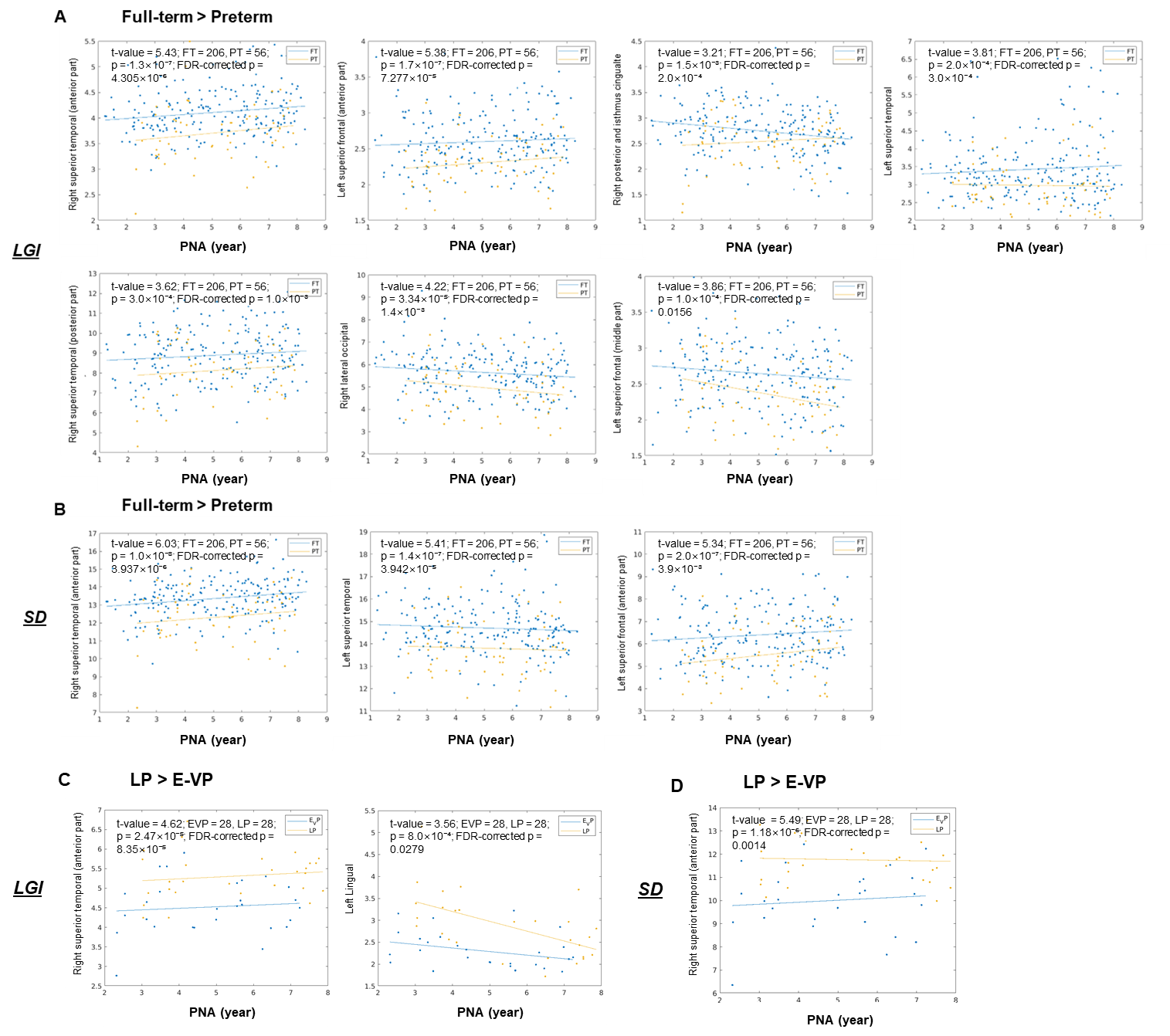


**Supplementary Figure 4.** Scatterplot of all clusters in full-term > preterm contrast analysis (A and B) and LP > E-VP contrast analysis (C and D). **A**. Regions in which the local gyrification index was significantly lower in preterm than full-term children. **B.** Regions in which sulcal depth was significantly lower in preterm than full-term children. **C**. Regions in which the local gyrification index was significantly lower in E-VP than LP children. **D.** Regions in which sulcal depth was significantly lower in E-VP than LP children. In each scatter-plot, postnatal age (years) is plotted on the x-axis and the regional LGI or SD on the y-axis. Blue dots = individual full-term or E-VP cases; orange dots = individual preterm or LP cases. The scatter plot displays individual data points with fitted regression lines for each group, and the corresponding statistical test results (t-value, group sizes, p-value, FDR-corrected p-value, and interaction p-value) are reported within the figure. Abbreviations: FT, full-term; PT, preterm; E-VP, extremely-to-very preterm; LP, late preterm; LGI, local gyrification index; SD, sulcal depth; PNA, postnatal age.

**
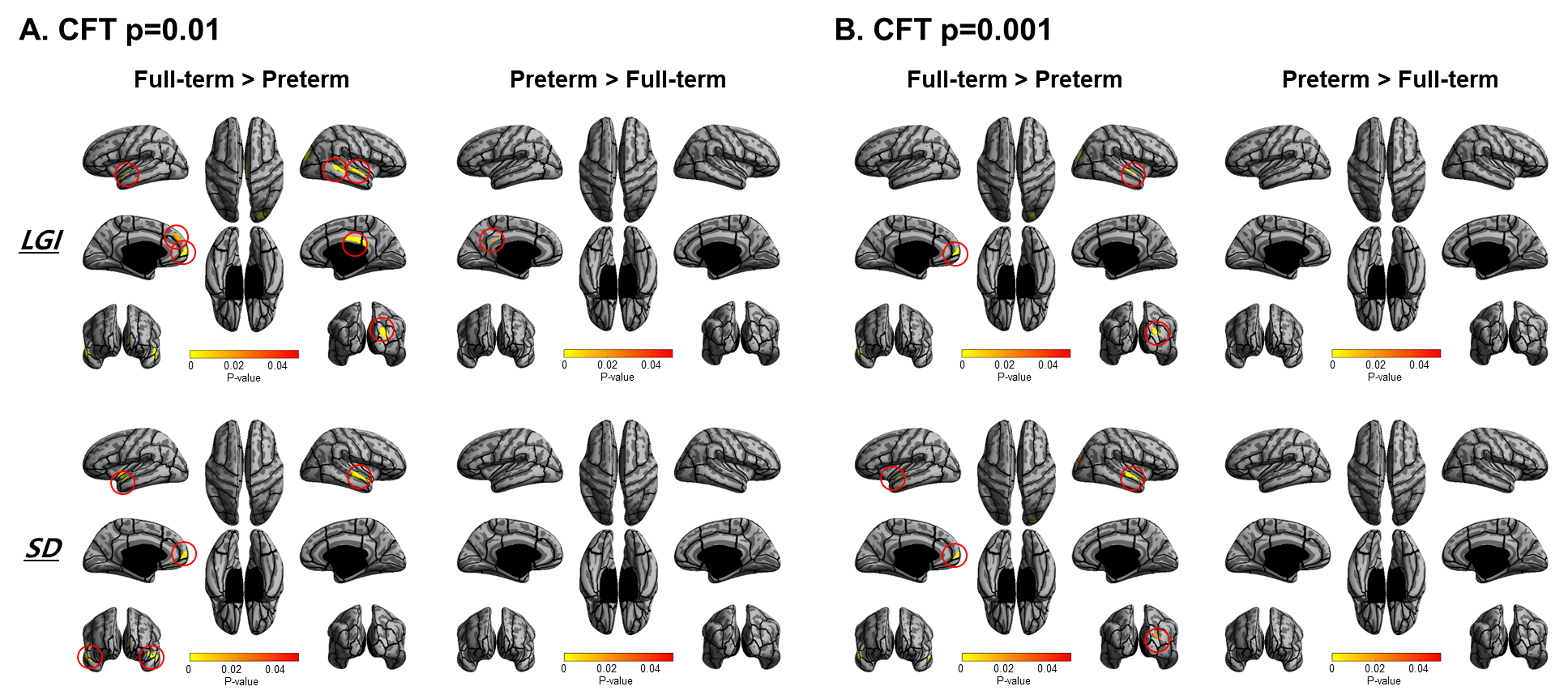
**

**Supplementary Figure 5. Preterm and full-term group differences in cortical measurements under two cluster-forming thresholds.** Regions of statistically significant group differences in LGI and SD are shown for each cluster-forming threshold (CFT = 0.01 [A] and CFT = 0.001 [B]), colored according to the cluster-corrected p-value (bottom scale). Abbreviations: CFT, cluster-forming threshold; FT, full-term infants; PT, preterm infants; LGI, local gyrification index; SD, sulcal depth.

**
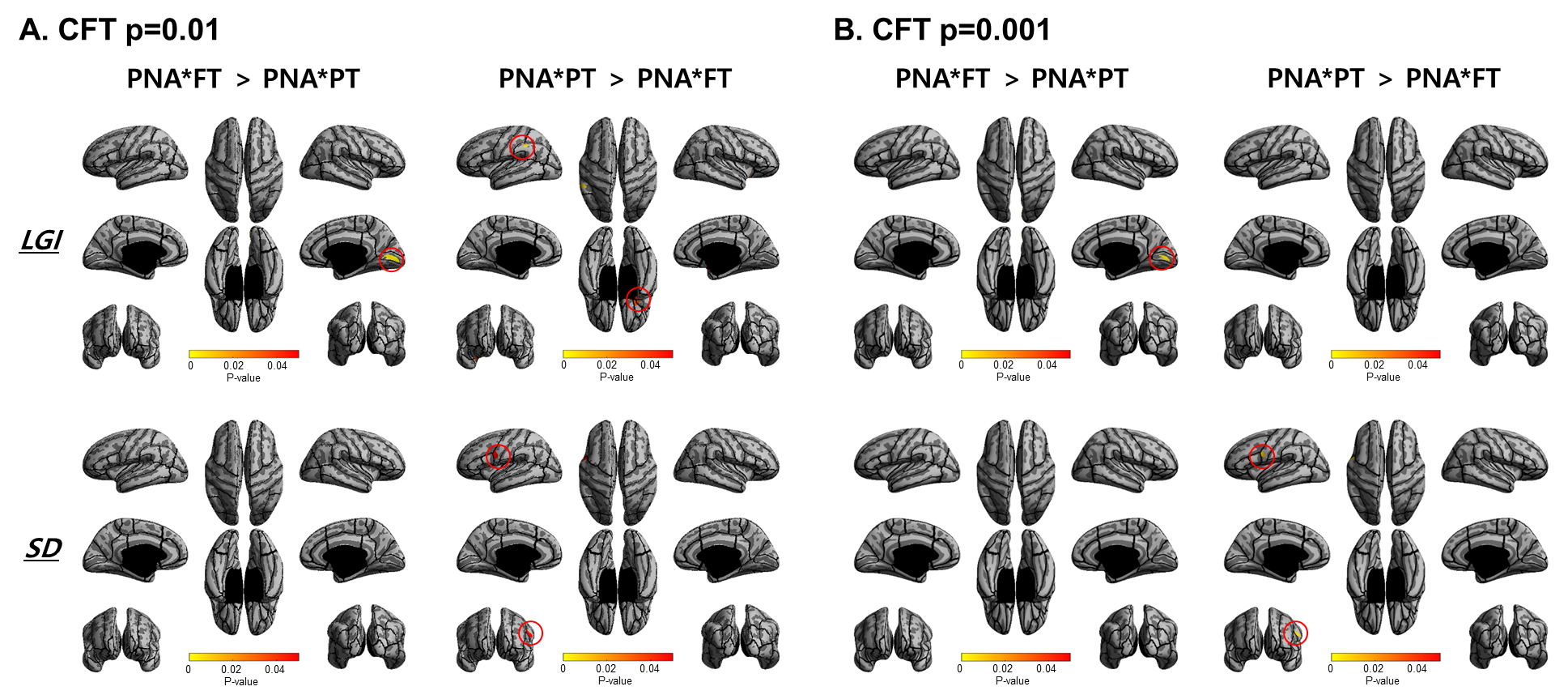
**

**Supplementary Figure 6. Statistically significant regions of PNA by group interactions by cortical measurements under two cluster-forming thresholds.** Regions of statistically significant PNA by group differences in LGI and SD are shown for each cluster-forming threshold (CFT = 0.01 [A] and CFT = 0.001 [B]), colored according to the cluster-corrected p-value (bottom scale). Abbreviations: CFT, cluster-forming threshold; FT, full-term infants; PT, preterm infants; PNA, postnatal age; LGI, local gyrification index; SD, sulcal depth.


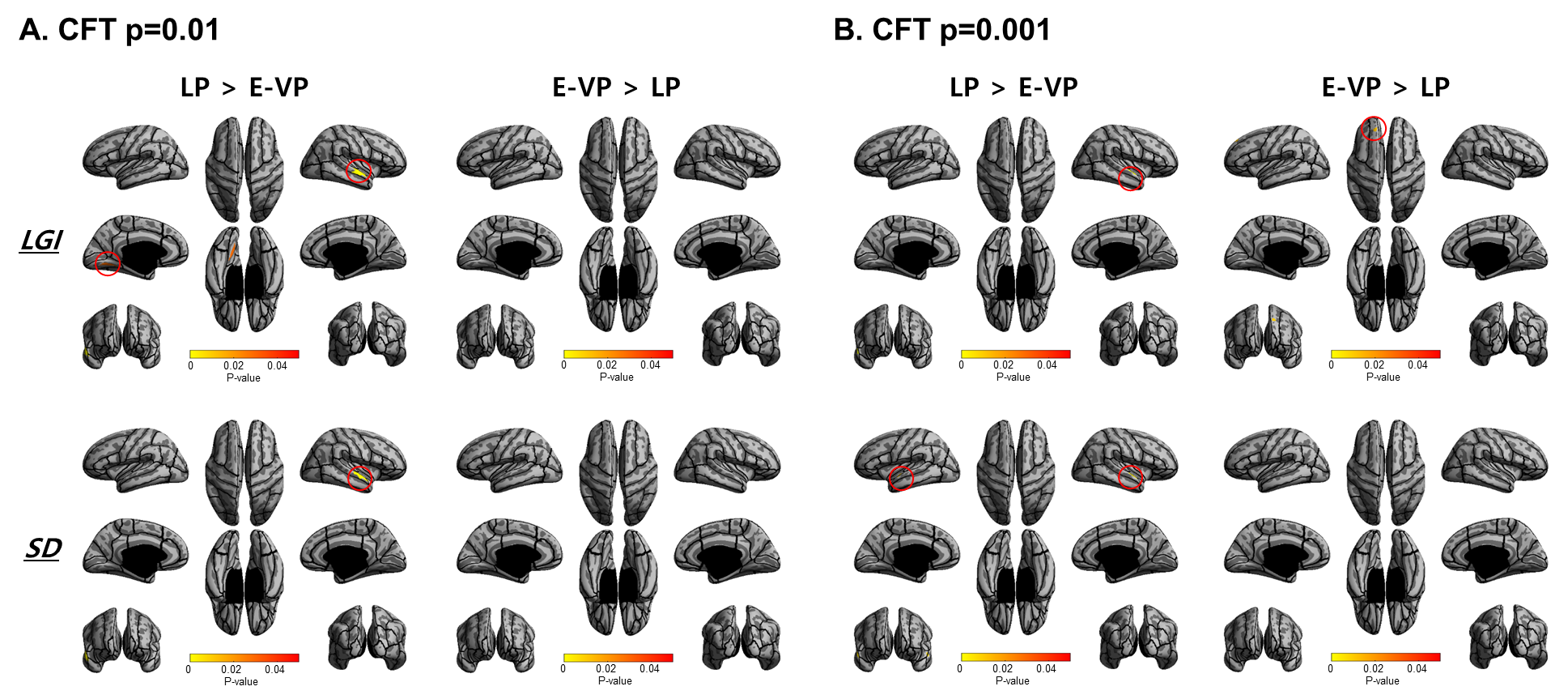


**Supplementary Figure 7. preterm subgroup differences in cortical measurements under two cluster-forming thresholds.** Regions of statistically significant preterm subgroup differences in LGI and SD are shown for each cluster-forming threshold (CFT = 0.01 [A] and CFT = 0.001 [B]), colored according to the cluster-corrected p-value (bottom scale). Abbreviations: CFT, cluster-forming threshold; LP, late preterm; E-VP, extremely-to-very preterm; LGI, local gyrification index; SD, sulcal depth.

**
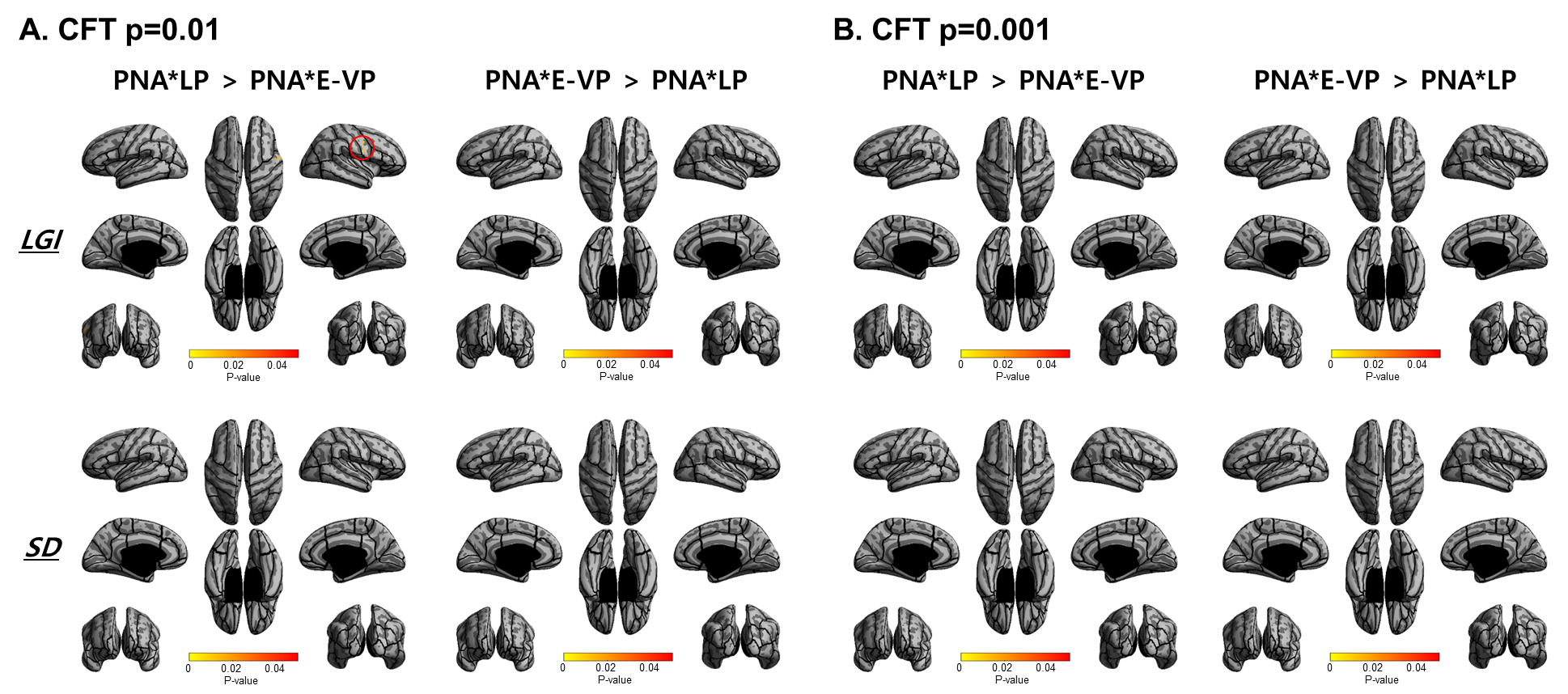
**

**Supplementary Figure 8. Statistically significant regions of PNA by preterm subgroup interactions by cortical measurements under two cluster-forming thresholds.** Regions of statistically significant PNA by preterm subgroup differences in LGI and SD are shown for each cluster-forming threshold (CFT = 0.01 [A] and CFT = 0.001 [B]), colored according to the cluster-corrected p-value (bottom scale). Abbreviations: CFT, cluster-forming threshold; LP, late preterm; E-VP, extremely-to-very preterm; PNA, postnatal age; LGI, local gyrification index; SD, sulcal depth.

**References**

1. Blumenthal JD, Zijdenbos A, Molloy E, Giedd JN. Motion artifact in magnetic resonance imaging: implications for automated analysis. *Neuroimage*. May 2002;16(1):89-92. doi:10.1006/nimg.2002.1076

2. Shaw P, Eckstrand K, Sharp W*, et al*. Attention-deficit/hyperactivity disorder is characterized by a delay in cortical maturation. *Proc Natl Acad Sci U S A*. Dec 4 2007;104(49):19649-54. doi:10.1073/pnas.0707741104

3. Tisdall MD, Reuter M, Qureshi A, Buckner RL, Fischl B, van der Kouwe AJW. Prospective motion correction with volumetric navigators (vNavs) reduces the bias and variance in brain morphometry induced by subject motion. *Neuroimage*. Feb 15 2016;127:11-22. doi:10.1016/j.neuroimage.2015.11.054

4. Guadalupe T, Mathias SR, vanErp TGM*, et al*. Human subcortical brain asymmetries in 15,847 people worldwide reveal effects of age and sex. *Brain Imaging Behav*. Oct 2017;11(5):1497-1514. doi:10.1007/s11682-016-9629-z

5. Rentería ME. Cerebral asymmetry: a quantitative, multifactorial, and plastic brain phenotype. *Twin Res Hum Genet*. Jun 2012;15(3):401-13. doi:10.1017/thg.2012.13

6. Alex AM, Aguate F, Botteron K*, et al*. A global multicohort study to map subcortical brain development and cognition in infancy and early childhood. *Nature neuroscience*. 2024;27(1):176-186.

7. Remer J, Croteau-Chonka E, Dean DC*, et al*. Quantifying cortical development in typically developing toddlers and young children, 1–6 years of age. *NeuroImage*. 2017/06/01/ 2017;153:246-261. doi:https://doi.org/10.1016/j.neuroimage.2017.04.010

8. Biagioni E, Frisone M, Laroche S*, et al*. Maturation of cerebral electrical activity and development of cortical folding in young very preterm infants. *Clinical neurophysiology*. 2007;118(1):53-59.

9. Shimony JS, Smyser CD, Wideman G*, et al*. Comparison of cortical folding measures for evaluation of developing human brain. *Neuroimage*. 2016;125:780-790.
