# Supplementary material for: Long-term effects of preterm birth on cortical folding trajectories in early childhood
Source: Brain Commun. 2026 May 18;8(3):fcag097. doi: 10.1093/braincomms/fcag097 (PMC13181400; doi:10.1093/braincomms/fcag097)
Supplement: fcag097_Supplementary_Data [file Original Submission - Author's Response.pdf › Original Submission - Author's Response.pdf]

Hanyang University College of Medicine

222, Wangsimli-ro
Seongdong-ku
Seoul
04763
Republic of Korea
Tel: +82-2-2290-8574

14th, December 2025

Tara Spires-Jones, PhD
Editor-in-Chief
Brain Communications

Dear Prof. Tara Spires-Jones,

Re: BRAINCOM-2025-821, "Long-term effects of preterm birth on cortical folding trajectories in early childhood"

We would like to submit our revised article entitled, “Long-term effects of preterm birth on cortical folding trajectories in early childhood” for consideration for publication as a full-length article in *Brain Communications.*

We have made appropriate corrections and clarifications. Again, each of the co-authors has reviewed and agreed with each of the changes made to this manuscript in the revision.

Please find these revisions detailed below.

GENERAL

The major concerns raised by the reviewers and the corresponding revisions in the manuscript are summarized as follows:

1. **Methodological clarity of cortical folding metrics**: Reviewers questioned the transparency and validity of the processing pipeline, including the use of FreeSurfer in a pediatric cohort, the implementation of shape-adaptive LGI, and the non–FreeSurfer–based computation of sulcal depth.
   A. We clarified the LGI/SD computation pipeline, detailed the pediatric QC procedures, and explicitly stated that metrics were derived using our own vertex-wise framework rather than default FreeSurfer outputs.
2. **Sample size and generalizability:** Concerns were raised regarding the relatively small preterm sample, the broad developmental age range, and the limited number of participants with neurodevelopmental assessments, all of which may constrain the robustness and generalizability of the findings.
   A. We added explicit statements acknowledging the modest preterm sample and limited neurodevelopmental data, emphasizing that neurodevelopmental findings are preliminary and should be interpreted cautiously.
3. **Modeling of developmental trajectories:** Reviewers requested justification for treating age as a linear effect, noting that early brain development can follow nonlinear trajectories.
   A. We justified the use of a linear age term by adding nested model testing results showing no improvement with nonlinear terms within the 1–7-year range.
4. **Handling of site effects:** The use of covariate adjustment rather than harmonization methods such as ComBat required clearer justification, particularly given the multi-site design.
   A. We added justification for using covariate adjustment, noting harmonized MRI acquisition protocols across sites and potential risks of biological variance loss with ComBat.
5. **Statistical thresholding and false-positive risk:** The cluster-defining threshold of $p < 0.01$ was viewed as liberal for surface-based neuroimaging, raising concerns about inflated false positives.
   A. We included supplementary analyses using a stricter $p < 0.001$ threshold and acknowledged the higher false-positive risk associated with the chosen threshold.
6. **Interpretative strength and clarity:** Reviewers noted that several statements in the Discussion were overly strong or insufficiently supported, and requested more balanced wording and clearer conceptual grounding for the interpretation of LGI/SD findings and structure–function relationships.
   A. We revised the Discussion by softening overstated claims about STG structure–function relationships, clarifying that neurodevelopmental finding is preliminary, and expanding the developmental context for LGI/SD and cortical folding theories to ensure balanced and well-supported interpretations.

SPECIFIC

Thank you very much for all the valuable comments that have helped improve the manuscript. We have addressed the point-by-point comments below:

A three-column table has been provided to summarize our revisions, including (i) /Reviewers' suggestions, questions, or comments, (ii) summary of responses from the authors, and (iii) changed parts with yellow highlight.

In the present paper, the sentences and paragraphs mainly in the abstract sections have been overall changed in accordance with the reviewer's suggestion. Please check the attached "Revised_manuscript_for_reviewer" for details.

Comment from Reviewer #1	Author's Response	Changes in the manuscript
**Comment 1**. Several aspects of the methods, particularly concerning Freesurfer, are unclear or confusing.	Thank you for your thoughtful comment. Your concern regarding the methodology, particularly the use of FreeSurfer in pediatric analyses, is valid. We agree with this point and have provided a detailed response to the reviewer's question in the revised manuscript.	-
**Comment 1.a.** Freesurfer is generally accepted to work well on adult humans. However, this study focused on children from 2-7. How was this method verified to work well on this subject set?	Thank you for raising the important point regarding the applicability of FreeSurfer to a 1–7-year-old pediatric cohort. We agree with the reviewer that FreeSurfer is known to perform best on adult human brains. Although there are relatively few studies focusing on children, FreeSurfer is still commonly used in this population [1,2,3]. In our study, all MRI data underwent extensive quality assurance (QA) procedures as also detailed in the supplementary materials.<br><br>Quality control was performed by two independent raters experienced in pediatric neuroimaging. All outputs from both the segmentation stage and subsequent cortical parcellation stage were cross-reviewed. Because common issues in children arise from intensity-normalization failures that can propagate to inaccurate segmentation and incorrect	**In the Materials and methods section (lines 186 to 187):**<br>**Added:** Although there are relatively few studies focusing on children, FreeSurfer is still commonly used in this population.[55-57].

	cortical parcellation—particularly in regions such as the temporal lobe—we specifically screened for these problems using the ENIGMA QC protocol, which highlights typical segmentation and parcellation errors. Based on prior experience with a range of pediatric mis-segmentation and mis-parcellation cases, we reprocessed or corrected datasets when necessary. Although we acknowledge that a fully standardized validation framework for FreeSurfer in this specific age range is still lacking, our QC procedures followed approaches commonly used in previous pediatric studies [1–3], and we adopted comparable principles to assess the suitability of our segmentation and parcellation outputs.<br><br>[1] Wedderburn, Catherine J., et al. "Neuroimaging young children and associations with neurocognitive development in a South African birth cohort study." *Neuroimage* 219 (2020): 116846.<br>[2] Pulli, Elmo P., et al. "Feasibility of FreeSurfer processing for T1-weighted brain images of 5-year-olds: semiautomated protocol of FinnBrain Neuroimaging Lab." *Frontiers in Neuroscience* 16 (2022): 874062.<br>[3] Beelen, Caroline, et al. "Investigating the added value of FreeSurfer's manual editing procedure for the study of the reading network in a pediatric population." *Frontiers in human neuroscience* 14 (2020): 143.	
**Comment 1.b.** The calculations of SD Comments a cerebral hull surface – was this calculated using Freesurfer? If so, Freesurfer's calculations of sulcal depth are actually based on a hypothetical midcortical surface, such that gyri	Thank you for highlighting the need for clarification regarding the basis of our sulcal depth calculations. FreeSurfer's sulcal depth (or *sulc*) measure is known as *average convexity,* which represents the signed distance that a vertex on the white matter surface moves during the inflation process. [4]. In our study, we rather followed [5] for sulcal depth computation. Unlike FreeSurfer's approach, this method creates	**In the Materials and methods section (lines 198 to 201):**<br>**Before:** SD refers to the shortest Laplacian trajectory from the cerebral hull to its corresponding cortical surface vertex.[56]<br>**After:** SD was defined as the shortest trajectory from the cerebral hull to the cortical surface, obtained by solving a constant-speed Eikonal equation between

have negative sulcal depth. Please clarify.	the cerebral hull of the pial surface and computes geometric distance between them. In this approach, sulcal depth was defined as the shortest trajectory from the cerebral hull to the cortical surface, obtained by solving a constant-speed Eikonal equation between the two surfaces. Therefore, our sulcal depth measure differs fundamentally in both definition and physical interpretation. The sulcal depth is derived from a non-negative distance and strictly positive (≥ 0). The detailed mathematical formulation and procedural description of the sulcal depth computation can be found in Section 2.2 (“Sulcal Depth”) of reference [5].<br><br>[4] Fischl, Bruce, Martin I. Sereno, and Anders M. Dale. "Cortical surface-based analysis: II: inflation, flattening, and a surface-based coordinate system." *Neuroimage* 9.2 (1999): 195-207.<br>[5] Lyu, Ilwoo, et al. "Sulcal depth-based cortical shape analysis in normal healthy control and schizophrenia groups." *Proceedings of Spie--the International Society for Optical Engineering*. Vol. 10574. 2018.	two surfaces.[59] Since SD is derived from a non-negative distance, its values are strictly positive and represent absolute geometric depth.
**Comment 1.c.** What cortical atlas was used for parcellation? How many regions does it include? How was it applied?	Thank you for your question regarding the cortical parcellation atlas and its implementation in our analysis. The statistical analysis was conducted via cortical surface registration on a vertex-wise basis, without the need for cortical parcellation. Cortical surface parcellation was used only for visualization purposes by projecting the results onto a template surface. In this process, FreeSurfer’s default cortical atlas, the Desikan-Killiany-Tourville cortical labeling protocol with 31 labels [6] was used. Specifically, we used the “lh.aparc.DKTatlas.annot” file located in the label directory of each subject.	**In the Materials and methods section (lines 246 to 250):**<br>**Added:** The statistical analysis was conducted via cortical surface registration on a vertex-wise basis, without the need for cortical parcellation. Cortical surface parcellation was used only for visualization purposes by projecting the results onto a template surface. In this process, FreeSurfer’s default cortical atlas, the Desikan-Killiany-Tourville cortical labeling protocol with 31 labels was used.[64]

<table>
<tr><td></td><td>[6] Klein, Arno, and Jason Tourville. "101 labeled brain images and a consistent human cortical labeling protocol." Frontiers in neuroscience 6 (2012): 171.</td><td></td></tr>
<tr><td>Comment 1.d. L132 Comments a “shape-adaptive” LGI measure. What is this, and how was it measured?</td><td>Thank you for pointing out the need to clarify the definition and implementation of the shape-adaptive LGI measure. The shape-adaptive LGI was first introduced by [1], as a refinement of the conventional FreeSurfer’s local gyrification index. The conventional approach uses a Euclidean sphere to compute the ratio between the cortical (pial) surface area and its corresponding outer hull within a local kernel (see Fig.R1).
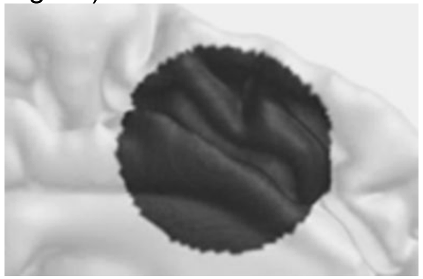
Figure R1. Conventional kernel based on a Euclidean sphere.
The conventional approach has several limitations: First, the conventional approach uses an unadjusted, fixed kernel size regardless of brain size. This leads to inconsistent results even for same cortical shapes. While some studies have adjusted these metrics in linear models, linear adjustments less suitable because the metric captures the area ratio. Second, the conventional kernel shape ignores the geometry of sulci and gyri, often spanning multiple folds and thus blurring spatial specificity. As a result, conventional approach fails to capture functional homogeneity that typically exists within individual sulcal or gyral regions.</td><td>In the Materials and methods section (lines 201 to 208):
Before: LGI is a ratio representing the proportion of the cortical surface relative to the cerebral hull. [57]

After: LGI was computed using a shape-adaptive LGI introduced as a refinement of the conventional FreeSurfer’s LGI.[60] A shape-adaptive LGI defines a spatially varying kernel that adapts to the local cortical folding. This is because the adaptive kernel is constructed through anisotropic wavefront propagation guided by a tensor field derived from cortical folding patterns. Moreover, the kernel size is globally rescaled according to the cortical surface area across different age groups to account for brain size variability. Together, these adaptations show higher reproducibility in multi-scan dataset compared to FreeSurfer’s conventional LGI.</td></tr>
</table>

	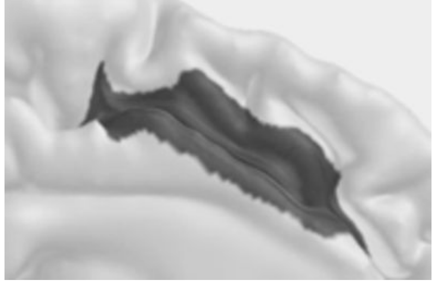<br>Figure R2. Shape-adaptive kernel adapts to the local cortical folding geometry.<br><br>On the other hand, the shape-adaptive LGI defines a spatially varying kernel that adapts to the local cortical folding (see Fig. R2). This is because the adaptive kernel is constructed through anisotropic wavefront propagation guided by a tensor field derived from cortical folding patterns. Moreover, the kernel size is globally rescaled according to cortical surface area across different age groups to account for brain-size variablity. Together, these adaptations show higher reproducibilty in multi-scan dataset compared to FreeSurfer's conventional LGI.<br><br>[1] Lyu, Ilwoo, et al. "A cortical shape-adaptive approach to local gyrification index." *Medical image analysis* 48 (2018): 244-258.	
**Comment 2.** The study included subjects recruited from two hospitals. However, beyond including a random effect for the hospitals, there is no discussion about the two hospitals. In particular, I am interested to know if there are any demographic, geographic, socioeconomic, etc. differences between the two that	Thank you for raising this important point regarding potential demographic, geographic, or socioeconomic differences between the two hospitals. Individual-level socioeconomic indicators were not available in our dataset, which we acknowledge as a limitation. Both study sites, however, are tertiary care centers located in metropolitan regions of South Korea and operate within the same universal National Health Insurance system, which minimizes financial barriers to high-acuity neonatal services. In addition, both centers	**In the Materials and methods section (lines 147 to 149):**<br>**Added:** The cohorts from both institutions were recruited at tertiary care centers within South Korea's National Health Insurance system, and they shared similar socioeconomic conditions in access to neonatal and long-term developmental services.

might be of interest when studying neurodevelopment?	provide standardized access to post-discharge neurodevelopmental follow-up and early intervention programs under the national insurance framework, which further mitigates socioeconomic disparities in subsequent care.<br><br>Neonatal care in Korea is delivered through a nationally regionalized NICU network, and tertiary centers have been described as providing broadly comparable levels of intensive neonatal support [1]. National system-wide investment has reduced regional disparities and improved accessibility to intensive neonatal care, with evidence suggesting that neonatal outcomes are more strongly associated with NICU level and case volume than with underlying socioeconomic or geographic differences [2,3]. Importantly, both hospitals in our study are high-level tertiary NICU institutions with similar care capacity and admission volume ranges, indicating that differences in NICU level or case load are unlikely to introduce systematic bias between sites.<br><br>Therefore, while we cannot entirely exclude subtle unmeasured variation, the structure of the Korean perinatal care system, combined with the comparable NICU level and service capacity across the two hospitals, suggests that major socioeconomic or care-access disparities affecting neurodevelopmental outcomes are unlikely. In this context, the random-effect term for recruitment site is expected to have captured most institution-related variance, and residual site-driven confounding is likely to be minimal.<br><br>[1] Chang, Y. S., Park, W. S., Chung, S. H., & Lee, M. H. (2011). Regionalization of neonatal intensive care	

	in Korea. Korean Journal of Pediatrics, 54(12), 481–488. https://doi.org/10.3345/kjp.2011.54.12.481<br><br>[2] Shim, J. W., Kim, M. J., Kim, E. K., Park, H. K., Lee, S. M., Kim, S. S., & Chang, Y. S. (2013). The impact of neonatal care resources on regional variation in neonatal mortality among very low birthweight infants in Korea. Paediatric and Perinatal Epidemiology, 27(2), 216–225. https://doi.org/10.1111/ppe.12034<br><br>[3] Song, I. G., Lee, S. M., Chung, S. H., Kim, E. S., Kim, C. H., Park, W. S., & Chang, Y. S. (2018). Improved regional disparities in neonatal care by government investment in Korea. Journal of Korean Medical Science, 33(e43), 1–7. https://doi.org/10.3346/jkms.2018.33.e43	
**Comment 3.** Several claims in the paper were not well supported or explained.	-	-
**Comment 3.a.** "Preterm birth not only alters folding morphology in the STG but also fundamentally reshapes the relationship between folding metrics and neurodevelopmental outcomes."	We thank the reviewer for this comment. We agree that the original expression was unnecessarily strong and not fully integrated with the surrounding context. To resolve this, we made a minor adjustment to the wording so that the statement fits more appropriately within the flow of the Discussion.<br><br>The results related to this discussion have been reviewed and revised according to our response to Reviewer 2, Comment 6, and the corresponding updates have been incorporated into both the Results and Discussion sections.<br><br>Furthermore, regarding the neurodevelopmental analyses, we acknowledge that only a subset of preterm and full-term children completed standardized testing. We therefore clarified that these	**In the Discussion section (lines 387 to 392):**<br>**Before:** Similarly, we found that preterm birth not only alters folding morphology in the STG but also fundamentally reshapes the relationship between folding metrics and neurodevelopmental outcomes. In the right STG, a higher LGI and deeper SD were positively associated with VCI, WMI, and FSIQ in full-term children, whereas these same metrics exhibited moderate to strong negative correlations in the preterm group. This inversion indicates that the atypical folding patterns resulting from early extrauterine exposure fail to confer and may even compromise the neurodevelopmental advantages observed during typical development.<br><br>**After:** Similarly, we found that the association between SD and VCI, WMI, and FSIQ was markedly reduced in

	findings should be interpreted cautiously and considered preliminary evidence linking cortical folding alterations to emerging neurodevelopmental profiles.<br><br>We believe that these revisions improve the consistency of the narrative and more clearly convey the implications and limitations of our results. The manuscript has been updated accordingly.	the preterm children (with a similar pattern for WMI in the left STG). These results highlight that preterm birth not only alters folding morphology but also that the atypical folding patterns may associated with early extrauterine exposure can fail to confer, and may even diminish, the neurodevelopmental advantages observed during normative development.

**Comment 3.b.** "Compared to the full-term group, the preterm group exhibited lower LGI and SD in both the STG and left SFG, indicating impaired gyral and sulcal maturation, which potentially limits experience-dependent plasticity in higher-order cognitive regions."	We sincerely thank the reviewer for this valuable and insightful comment. We understand that the reviewer found the interpretive strength of the original argument to be limited, particularly in the transition between the observed reductions in LGI/SD and their developmental significance. Although the initial version presented relevant literature, we agree that the mechanistic linkage between gyrification metrics and postnatal neuroplastic potential required further conceptual grounding to support the stated implication.<br><br>To address this, we revised the discussion by integrating additional developmental rationale and structural frameworks. Specifically, we supplemented the paragraph with evidence showing that gyrification within temporal and frontal association cortices follows a perinatal growth trajectory that continues after birth, and we clarified how this trajectory reflects prolonged maturation through synaptic pruning, myelination, and long-range connectivity formation. We further articulated the theoretical basis in which tangential expansion and axonal–synaptic growth pressures contribute to folding architecture, thereby strengthening the logical bridge between altered LGI/SD and constraints on experience-dependent plasticity. These revisions were made while retaining the original structure and intent, but now more clearly anchor our interpretation within neurodevelopmental mechanisms and prior empirical findings.<br><br>We believe that these changes improve readability and conceptual continuity, and better justify why reduced LGI/SD in the STG and SFG may reflect delayed or incomplete maturation in domains supporting higher-order cognition. We appreciate the reviewer's guidance, which helped us refine the	**In the Discussion section (lines 353 to 355):**<br>**Before:** Compared to the full-term group, the preterm group exhibited lower LGI and SD in both the STG and left SFG, indicating impaired gyral and sulcal maturation, which potentially limits experience-dependent plasticity in higher-order cognitive regions.<br><br>**After:** Compared to the full-term group, the preterm group exhibited lower LGI and SD in both the STG and left SFG, reflecting altered gyral and sulcal maturation that could potentially restrict experience-dependent plasticity in higher-order cognitive regions.<br><br>**In the Discussion section (lines 357 to 378):**<br>**Before:** These regions are structurally important owing to their close association with higher-order cognitive functions during postnatal development.[63] Ronan *et al.*[64] reported that cortical regions related to complex cognitive processes exhibit spatially heterogeneous rates of tangential expansion, while Hill *et al.* [65] suggested that these regions maintain relatively low levels of structural maturity during the prenatal period to maximize experience-dependent neural plasticity after birth. In this context, experience-dependent neuroplasticity is promoted in typical brain development; however, reductions in LGI and SD observed in the STG and SFG suggest that preterm birth may be associated with delayed or incomplete structural maturation, potentially limiting the engagement of neuroplastic mechanisms in regions subserving higher-order cognitive functions.<br><br>**After:** Previous studies using surface-based analyses of cortical gyrification in healthy preterm and full-term infants have shown that folding progression is particularly marked within temporal and frontal association cortices across the perinatal period[11,67],

	argument and reinforce the developmental interpretation of our findings.	and this regionally elevated growth trajectory appears to extend into the first two years after birth.[15] This developmental profile aligns with a hierarchical maturation process in which association cortices gradually mature over an extended period through synaptic pruning, progressive myelination, and the establishment of long-range associative connections.[25,68,69] Moreover, the expansion of axonal and synaptic architecture imposes increasing tangential growth demands on the cortical sheet, which can manifest as gyrification patterns that facilitate the efficient spatial embedding of distributed networks.[70] Together, these folding mechanisms form a structural basis that enables experience-dependent refinement after birth and supports the emergence of higher-order cognitive functions. Interestingly, Hill *et al.*[71] proposed that cortical regions related to higher-order cognitive processing maintain comparatively low levels of brain morphological maturity during the fetal period to preserve as window for postnatal experience-dependent neuroplasticity. Ronan *et al.*[72] further reported that these regions undergo spatially heterogeneous tangential expansion after birth, suggesting that such variability may be a typical developmental feature supporting experience-dependent refinement. Given that gyrification is informative in capturing structural variability across cortical regions[73-75], reductions in LGI and SD observed in the STG and SFG suggest that preterm birth may be associated with delayed or incomplete structural maturation, potentially limiting the engagement of neuroplastic mechanisms in regions subserving higher-order cognitive functions.

**Comment 4.** 4. A few wordings are unclear or possibly incorrect:	-	-
**Comment 4.a.** Paragraph starting on L371: two theories of cortical folding are introduced here to explain the abnormal folding patterns in preterm children. However, these theories are not only relevant to certain regions of the brain or certain pathologies; rather, they are hypothesized to be the driving force for cortical folding throughout the brain. It might make sense to discuss how these mechanisms are perturbed or changed in premature birth, but the discussion as it stands is misleading.	We thank the reviewer for this helpful clarification. We agree that the original wording could unintentionally suggest that these theoretical models directly explain region-specific abnormalities. To prevent this misunderstanding, we revised the introductory sentence so that the theories are presented as broader mechanistic frameworks within which the observed atypical folding patterns may be interpreted. The sentence now reads:<br>"These atypical cortical folding patterns may be interpreted within the context of the following mechanistic frameworks."<br>This wording more accurately reflects the intended scope of the theories while maintaining consistency with the overall discussion. The manuscript has been updated accordingly.	**In the Discussion section (lines 406 to 407):**<br>**Before:** Two prevailing theories of cortical folding contextualize these findings.<br><br>**After:** These atypical cortical folding patterns may be interpreted within the context of the following mechanistic frameworks.
**Comment 4.b. L16:** "global" impairment, while the results showed changes in only a few select regions	Thank you for this insightful comment. We agree that the term "global impairment pattern" does not accurately represent our findings, which are region-specific rather than global. Accordingly, we have revised the Abstract and replaced this phrase with "region-specific impairment pattern" to more precisely reflect the anatomical distribution of the observed effects. We have also applied the same revision to the corresponding sentence in the first paragraph of the Discussion for consistency.	**In the Abstract (Results) section (lines 16 to 18):**<br>**Before:** Compared with their full-term peers, preterm children exhibited a global impairment pattern characterized by a significantly reduced local gyrification index and sulcal depth in the bilateral superior temporal gyrus and left superior frontal gyrus (*P* < 0.05).<br><br>**After:** Compared with their full-term peers, preterm children exhibited a region-specific impairment pattern characterized by a significantly reduced local gyrification index and sulcal depth in the bilateral superior temporal gyrus and left superior frontal gyrus (*P* < 0.05).<br>**In the Discussion section (lines 347 to 349):**<br>**Before:** Compared with their full-term counterparts, preterm infants exhibited significantly reduced LGI and SD in the bilateral STG and left SFG, suggesting a global disruption of perinatal cortical morphogenesis

		**After:** Compared with their full-term counterparts, preterm infants exhibited significantly reduced LGI and SD in the bilateral STG and left SFG, suggesting a region-specific disruption of perinatal cortical morphogenesis.
**Comment 4.c. L71:** What is meant by "asymmetrical" growth? Perhaps heterogeneous instead?	We appreciate the reviewer's careful observation. The term "asymmetrical" may indeed cause confusion in this context, as our intention was to describe region-specific heterogeneous growth rather than hemispheric asymmetry. To avoid misunderstanding, we have replaced "asymmetrical" with "heterogeneous" to better reflect the intended meaning. The manuscript has been updated accordingly.	**In the Introduction section (lines 68 to 71):**<br>**Before:** Leading theoretical models have proposed that cortical folding is either induced by axonal tension along long-range white matter tracts[12] or continuously modified by local mechanical instability arising from region-specific asymmetrical growth in the outer cortical layers.<br><br>**After:** Leading theoretical models have proposed that cortical folding is either induced by axonal tension along long-range white matter tracts[12] or continuously modified by local mechanical instability arising from region-specific heterogeneous growth in the outer cortical layers.[13,14]

Comment from Reviewer #2	Author's Response	Described or changed parts
**Comment 1**. The preterm group includes only 56 participants across a broad age range (2–7 years), which is a period of rapid and non-linear brain development. With such a small sample, it's hard to know whether the study can really capture meaningful age-related differences. On top of that, only 22 preterm and 22 full-term children completed cognitive testing, yet these results are highlighted as a key finding in the abstract. These small samples challenge the generalizability of the	We thank the reviewer for this important comment. We fully agree that the preterm sample spans a broad developmental window (1–7 years) and that the number of children who completed neurodevelopmental assessments is relatively small. We have clarified these issues in the revised manuscript and now explicitly acknowledge that the modest sample size limits the precision with which non-linear age-related effects can be characterized.<br><br>Regarding the neurodevelopmental analyses, we recognize that only a subset of preterm and full-term children completed standardized testing. We agree that these results should be interpreted cautiously and primarily as preliminary evidence linking cortical	**In the Discussion (Limitation) section (lines 458 to 463):**<br>**Added:** Second, the preterm sample size was not sufficiently large to capture the broad developmental window (1–7 years) during which cortical maturation progresses rapidly, which may reduce the precision of age-related inferences. These results should be interpreted cautiously and validated in larger cohorts. Third, only a subset of children completed cognitive assessments, limiting the robustness of neurodevelopmental associations. These results should be viewed as preliminary and require replication with broader behavioral sampling.

findings, and this should be clearly acknowledged as a major limitation.	folding alterations to neurodevelopmental profiles. We have revised the Abstract, Results, and Limitations to ensure that these findings are presented with appropriate restraint and without overstating their generalizability.<br><br>In the revised manuscript, we now emphasize that (1) the age range and sample size limit the ability to draw conclusions about precise developmental timing, (2) the neurodevelopmental results are exploratory and require replication in larger cohorts, and (3) future studies with longitudinal designs are needed to fully delineate developmental trajectories in preterm children.	
**Comment 2**. Relatedly, the authors treat age as a linear effect, even though early brain development is well known to follow nonlinear patterns. At the very least, the authors should explain why they chose a linear model and consider whether nonlinear terms (like quadratic or cubic) would better capture age-related effects.	We appreciate the reviewer's insightful comment regarding the potential nonlinearity of age-related effects in brain development. During early infancy (0–2 years), cortical growth is known to follow highly nonlinear trajectories, typically described by logarithmic, quadratic, or other higher-order models [8,9]. However, our cohort primarily includes children aged 1 to 7 years, a developmental window following the rapid postnatal expansion phase. Indeed, cortical folding measures such as those reported by [10,11] display largely linear age-related changes.<br>Building on this, we adopted a linear age term as an appropriate model for describing cortical development within our age range.<br>To empirically validate this assumption, we performed an F-test for nested linear model implemented in SurfStat to examine whether higher-order age terms significantly improved the model fit beyond a linear specification. For example, we compared the following nested models:<br><br>$measure1 = \beta_0 + \beta_1(PT/FT) + \beta_2 PNA + \beta_4 sex + u_{0j} + \epsilon \ (1)$	**In the Supplementary Text 2:**<br>**Added:** To ensure that the linear modeling of age adequately captures developmental trajectories within our cohort, we conducted an empirical validation of the age effect model. During early infancy (0-2 years), cortical growth has been shown to follow highly nonlinear trajectories, often characterized by logarithmic or quadratic patterns.[6,7] However, our dataset primarily includes children aged 1-7 years, a developmental window following the rapid postnatal expansion phase. Prior studies have reported that cortical folding measures already exhibit approximately linear age-related changes even within the 0-2 year period.[8,9] Based on these findings, we adopted a linear age term as an appropriate model for cortical development in our age range.<br>To verify this assumption, we performed an F-test for nested linear models using SurfStat to assess whether the inclusion of higher-order age terms improved the model fit beyond the linear specification. We test the following linear model (example):<br>$measure1 = \beta_0 + \beta_1(PT/FT) + \beta_2 PNA + \beta_4 sex + u_{0j} + \epsilon \ (1)$

	$$measure2 = measure1 + \beta_4 PNA^2$$ The F-test procedure was applied to both FT-PT and E-VP-LP group comparisons, as well as to each corresponding interaction term. The results revealed that the quadratic term did not yield any significant clusters in any of the analysis after multiple comparison correction (RFT, cluster threshold=0.01). These results indicate that within our 1 to 7-year-old sample, the age-related variations in cortical folding are sufficiently captured by a linear age effect, with no additional explanatory benefit from higher-order terms. This finding aligns with prior literature showing that cortical maturation during mid-to-late childhood follows an approximately linear developmental trajectory [8].<br><br>[8] Remer, Justin, et al. "Quantifying cortical development in typically developing toddlers and young children, 1–6 years of age." *Neuroimage* 153 (2017): 246-261.<br>[9] Alex, Ann M., et al. "A global multicohort study to map subcortical brain development and cognition in infancy and early childhood." *Nature neuroscience* 27.1 (2024): 176-186.<br>[10] Biagioni, E., et al. "Maturation of cerebral electrical activity and development of cortical folding in young very preterm infants." *Clinical neurophysiology* 118.1 (2007): 53-59.<br>[11] Shimony, Joshua S., et al. "Comparison of cortical folding measures for evaluation of developing human brain." *Neuroimage* 125 (2016): 780-790.	$$measure2 = measure1 + \beta_4 PNA^2 \ (2)$$ This analysis was applied to both the FT–PT and E-VP–LP group comparisons, as well as their interaction terms. The results showed that the quadratic term did not yield any significant clusters after multiple comparison correction. These findings indicate that within our 1-7 year-old sample, cortical folding variations are sufficiently described by a linear age effect, with no additional explanatory benefit from higher-order terms. This supports previous evidence suggesting that cortical maturation during mid-to-late childhood follows an approximately linear developmental trajectory[7]
**Comment 3**. The manuscript states that "written informed consent was obtained from all participants," which doesn't seem accurate given the age range. Some children were scanned as young as 2 years, and	Thank you for this important comment and for pointing out the ambiguity. We confirm that written informed consent was not obtained directly from the children, but rather from parents or legal guardians for all participants. For the preterm group, consent was obtained during the neonatal hospitalization	**In the Materials and methods section (lines 161 to 164):**<br>**Before:** The study protocol was prospectively approved by the Institutional Review Board of Hanyang University Hospital, and written informed

others were recruited within the first week of life. Please clarify how consent/assent was obtained from guardians and participants.	period shortly after birth. For the full-term comparison group, consent was obtained at the time of outpatient enrollment. We have revised the Methods to clarify these procedures to avoid misunderstanding.	consent was obtained from all participants prior to participation.<br><br>**After:** The study protocol was prospectively approved by the Institutional Review Board of Hanyang University Hospital, and written informed consent was obtained from parents or legal guardians at the time of neonatal enrollment as part of the hospital's neonatal follow-up program.
**Comment 4**. The authors address site effects using a covariate approach. I'm curious why more robust harmonization methods (e.g., ComBat) weren't considered, given that it has been shown to outperform covariate adjustment (e.g., Fortin et al., NeuroImage, 2018).	We thank the reviewer for raising the important issue of site effects and the potential use of ComBat for harmonization. Prior studies demonstrate that ComBat yields the strongest advantages in settings with substantial scanner- or protocol-related heterogeneity across many sites [1,2]. However, we selected a covariate-based approach for the following methodological considerations.<br><br>First, because both institutions participate in the Korean Neonatal Network (https://www.knn.or.kr/main), MRI acquisition was collaboratively standardized for research purposes, resulting in a unified single-vendor, single-protocol imaging framework. Consequently, both imaging sites used fully harmonized MRI acquisition protocols, and cohort construction was explicitly controlled to maintain protocol equivalence.<br><br>Second, recent methodological work has shown that ComBat may inadvertently remove biologically meaningful variance when biological covariates (e.g., age) are partially collinear with site.<br>Benkarim et al. explicitly caution that "ComBat might also remove the effects of other factors (e.g., age and sex) if they are collinear with site," leading to unintended loss of variance related to true developmental or clinical factors [3]. Similarly,	**In the Materials and methods section (lines 170 to 172):**<br>**Added:** Because both hospitals participate in the Korean Neonatal Network, MRI acquisition was collaboratively standardized for research purposes, resulting in a unified single-vendor, single-protocol imaging framework.

	harmonization frameworks reviewed by Wachinger et al. emphasize that aggressive site-equating procedures can “easily remove relevant subject-specific information” in certain scenarios [4]. Given that our study focuses on early morphological development (LGI and SD), preserving intrinsic biological variability was critical.<br><br>In summary, because (1) both imaging sites used intentionally harmonized acquisition protocols with minimal scanner-related variability and (2) ComBat carries a documented risk of removing biological variance when site structure overlaps with developmental covariates, we determined that covariate adjustment was the most appropriate method for this study.<br><br>[1] Fortin, J. P., Cullen, N., Sheline, Y. I., Taylor, W. D., Aselcioglu, I., Cook, P. A., … Shinohara, R. T. (2018). Harmonization of cortical thickness measurements across scanners and sites. NeuroImage, 167, 104–120.<br><br>[2] Pomponio, R., Erus, G., Habes, M., Doshi, J., Srinivasan, D., Mamourian, E., … Davatzikos, C. (2020). Harmonization of large MRI datasets for the analysis of brain imaging patterns throughout the lifespan. NeuroImage, 208, 116450.<br><br>[3] Benkarim, O., Villalón-Reina, J. E., Llera, A., Abdulkadir, A., Urchs, S., Langs, G., … Valk, S. L. (2022). Population heterogeneity in clinical cohorts affects the predictive accuracy of brain imaging. PLOS Biology, 20(12), e3001627.<br><br>[4] Wachinger, C., Rieckmann, A., & Reuter, M. (2020). Detect and correct bias in multisite	

	neuroimaging datasets. arXiv preprint arXiv:2010.05063.	
**Comment 5**. The authors use a cluster-defining threshold of p < 0.01, which is more liberal than the default in SurfStat (p < 0.001) and the standard in surface-based neuroimaging. This choice increases the risk of false positives, as shown in Eklund et al. (2016, PNAS). At minimum, results using the more conservative threshold should be included in the supplement. If findings don't hold up, that should be clearly stated as a major limitation	Thank you for raising this important point regarding our cluster-defining threshold and the need to address potential inflation of false positives. We acknowledge the reviewer's comment regarding the cluster-defining threshold employed in our manuscript. While no single threshold is universally endorsed within the statistical or neuroscience communities, we did not provide justification for our choice of $p < 0.01$ in the original submission. It is widely recognized that the $p < 0.001$ threshold is a conventional standard in surface-based neuroimaging. In our case, we adopted a slightly more liberal threshold ($p < 0.01$) to partially compensate for the limited sample size of the preterm group, which substantially reduced statistical power and made it difficult to detect significant clusters after multiple comparison correction. Nonetheless, we fully acknowledge that this choice increases the potential risk of false positives. In response to the reviewer's suggestion, we have included supplementary analyses using a stricter threshold of $p < 0.001$ and have discussed the discrepancies between the two sets of results as an important limitation. We expect that with larger datasets, future studies will achieve stronger statistical power and yield more stable and reproducible findings even under more conservative thresholds.	**In the Discussion (Limitation) section (lines 471 to 480):**<br>**Added:** One methodological limitation concerns the choice of the cluster threshold in multiple comparison correction. In this study, we applied a relatively liberal threshold of p < 0.01, which differs from the conventional standard in surface-based neuroimaging (p < 0.001). This decision was made to account for the limited sample size of the preterm group, which substantially reduced statistical power and made it difficult to detect significant clusters after multiple comparison correction. We acknowledge that this approach increases the potential risk of false positives. To address this issue, we additionally provide results based on a stricter threshold of p < 0.001 in the Supplementary Materials and discuss the discrepancies between the two results as an important limitation (Supplementary Fig. S5-8). Future studies with larger sample sizes are expected to resolve this limitation.
**Comment 6**. The authors use Fisher's r-to-z transformation to compare correlation strength between groups. Since linear models were already used elsewhere, it's unclear why interaction terms weren't used instead. That approach would allow	We thank the reviewer for this insightful comment. We agree that a linear-model framework incorporating group × cortical measure interaction terms provides a more coherent and interpretable approach for assessing group differences while maintaining consistent covariate adjustment. In line with this recommendation, we reanalyzed all structure–function associations using interaction	**In the Abstract (Results) section (lines 18 to 21):**<br>**Before:** Notably, cortical morphometric measures in the right superior temporal gyrus were positively associated with neurodevelopmental outcomes in full-term children ($P < 0.05$); however, this relationship was reversed in preterm children ($P < 0.05$). These opposing associations differed significantly between

for consistent covariate control and more interpretable results.	models that included postnatal age, and sex as covariates (and gestational age for the preterm group where applicable).<br><br>The updated results reveal that sulcal depth (SD) showed significant interaction effects across multiple neurodevelopmental domains in the right STG (VCI: $\beta = -18.48$, FDR $p = 0.014$; WMI: $\beta = -20.62$, FDR $p = 0.014$; FSIQ: $\beta = -17.65$, FDR $p = 0.031$), and a similar pattern was observed for WMI in the left STG ($\beta = -23.51$, FDR $p = 0.004$). In contrast, the interactions involving LGI did not reach statistical significance after FDR correction. These findings are now fully reflected in the revised Results (Table 3; Supplementary Tables 2 and 3).<br><br>Corresponding changes have been made throughout the manuscript to ensure methodological consistency and clarity of interpretation. We appreciate the reviewer's guidance, which has strengthened both the analytical coherence and interpretability of the work.	the two groups, indicating an atypical structure–function relationship in preterm children.<br><br>**After:** Notably, sulcal depth in the superior temporal cortex showed significant differences between preterm and full-term children in its association with neurodevelopmental outcomes ($P < 0.05$), indicating an atypical structure–function relationship in preterm children.<br><br>**In the Materials and methods section (lines 253 to 266):**<br>**Added:**<br>**Group comparisons of structure-function associations**<br>To evaluate whether the associations between cortical measures and neurodevelopmental outcomes differed between the preterm and full-term groups, we used linear regression models incorporating a group × cortical measure interaction term. For each cortical metric (SD and LGI) that showed significant group differences, separate models were constructed for the VCI, VSI, FRI, WMI, and FSIQ scores from the WPPSI-IV. The general model specification was:<br>$outcome = \beta_0 + \beta_1 measure + \beta_2 group + \beta_3 measure * group + \beta_4 PNA + \beta_5 sex + \epsilon, (3)$<br>where outcome represents VCI, VSI, FRI, WMI, or FSIQ; measure refers to SD or LGI; and group is coded as PT vs. FT. Analyses were adjusted for PNA and sex, and for analyses involving preterm children, GA was additionally included as a covariate. The interaction coefficient (β_3) was used to test whether the association between cortical measures and cognitive outcomes differed significantly between the groups. All p-values were corrected for multiple comparisons using the FDR procedure.

		**In the Results section (lines 328 to 340):**<br>**Added:**<br>**Group differences in associations between cortical measurements and neurodevelopmental outcomes**<br>Statistical analysis revealed group differences in the associations between cortical measures and neurodevelopmental outcomes based on linear models incorporating group × cortical measure interaction terms (Table 3). In the right superior temporal region, the interaction between SD and VCI was significant (β = –18.48, FDR p = 0.014), whereas the interaction for LGI did not reach statistical significance (β = –11.53, FDR p = 0.822). For WMI, LGI showed a nominal interaction effect (β = –32.47, FDR p = 0.129), while SD demonstrated a significant interaction (β = –20.62, FDR p = 0.014). For FSIQ, the interaction effect was not significant for LGI (β = –19.72, FDR p = 0.543), but SD showed a significant interaction (β = –17.65, FDR p = 0.031). In the left superior temporal region, LGI demonstrated a nominal interaction effect with WMI (β = –19.95, FDR p = 0.129), whereas SD exhibited a significant interaction (β = –23.51, FDR p = 0.004). Full results are provided in Supplementary Tables S2 and S3.<br><br>**In the Discussion section (lines 387 to 389):**<br>**Before:** Similarly, we found that preterm birth not only alters folding morphology in the STG but also fundamentally reshapes the relationship between folding metrics and neurodevelopmental outcomes. In the right STG, a higher LGI and deeper SD were positively associated with VCI, WMI, and FSIQ in full-term children, whereas these same metrics exhibited moderate to strong negative correlations in the preterm group.

		**After:** Similarly, we found that the association between SD and VCI, WMI, and FSIQ was markedly reduced in the preterm children (with a similar pattern for WMI in the left STG).
**Comment 7**. Units are missing in several places – for example, "years" for PNA, "weeks" for GA, and units for LGI/SD on color bars and axes. Please make sure all measures are clearly labeled.	Thank you for pointing this out. We agree with the reviewer that unit annotations were insufficient in several sections. We have reviewed the manuscript and figures comprehensively and added missing units where appropriate, including "years" for PNA, "weeks" for GA, and unit labels for LGI and SD on all relevant color bars and axes.	-
**Comment 8**. The stated age range throughout the manuscript is 2–7 years, but the PNA plot includes values <2 years. Please clarify the discrepancy.	We appreciate the reviewer's careful observation. Upon rechecking the dataset, we identified that a small subset of typically developing children (n = 7) had been enrolled between 15 and 24 months of age. The originally stated age range of 2–7 years was an inadvertent misstatement. We have therefore corrected all relevant sections of the manuscript to consistently indicate an age range of 1–7 years.	-
**Comment 9**. Abstract, Background: "…early extrauterine exposure…" – if this means preterm birth, consider simplifying for clarity.	We thank the reviewer for the helpful suggestion. We agree that the phrase "early extrauterine exposure" may be unclear, and we have simplified it to "preterm birth" in the Abstract and Background for greater clarity.	**In the Abstract (Background) section (lines 9 to 10):**<br>**Before:** However, the long-term impact of early extrauterine exposure-induced alterations in folding patterns remains unclear.<br><br>**After:** However, the long-term impact of folding alterations associated with preterm birth remains unclear.
**Comment 10**. Abstract, Methods: The claim of a more robust and accurate methodology is unclear, as SD and LGI via FreeSurfer is a standard approach for measuring cortical folding. Consider clarifying	Thank you for raising this point. Although our study extracted LGI and SD using the approach described in the Methods (see our responses to Reviewer 1, comments 1.b and 1.d), we acknowledge that the original wording in the Abstract may have unintentionally suggested reliance on standard FreeSurfer-derived metrics, as the reviewer noted.	**In the Abstract (Methods) section (lines 12 to 13):**<br>**Before:** We employed a robust methodology that more accurately captured microstructural alterations in cortical folding to derive cortical metrics.<br><br>**After:** We derived cortical metrics from the reconstructed cortical surfaces using a vertex-wise

what's novel or revising the language.	The LGI and SD metrics were derived through our own vertex-wise surface–registration pipeline, as described in the Methods. Cortical parcellation was applied only for visualization by projecting vertex-wise statistical results onto a template surface. We have clarified this distinction to prevent misunderstanding regarding the analytic approach.	computation framework to characterize regional folding patterns.
**Comment 11**. Abstract, Results: "global impairment pattern" – consider revising, as findings are region-specific, not global.	Thank you for this insightful comment. We agree that the term "global impairment pattern" does not accurately represent our findings, which are region-specific rather than global. Accordingly, we have revised the Abstract and replaced this phrase with "region-specific impairment pattern" to more precisely reflect the anatomical distribution of the observed effects. We have also applied the same revision to the corresponding sentence in the first paragraph of the Discussion for consistency.	**In the Abstract (Results) section (lines 16 to 18):**<br>**Before:** Compared with their full-term peers, preterm children exhibited a global impairment pattern characterized by a significantly reduced local gyrification index and sulcal depth in the bilateral superior temporal gyrus and left superior frontal gyrus ($P < 0.05$).<br><br>**After:** Compared with their full-term peers, preterm children exhibited a region-specific impairment pattern characterized by a significantly reduced local gyrification index and sulcal depth in the bilateral superior temporal gyrus and left superior frontal gyrus ($P < 0.05$).<br>**In the Discussion section (lines 347 to 349):**<br>**Before:** Compared with their full-term counterparts, preterm infants exhibited significantly reduced LGI and SD in the bilateral STG and left SFG, suggesting a global disruption of perinatal cortical morphogenesis<br><br>**After:** Compared with their full-term counterparts, preterm infants exhibited significantly reduced LGI and SD in the bilateral STG and left SFG, suggesting a region-specific disruption of perinatal cortical morphogenesis
**Comment 12**. Abstract, Results: all but the group difference of the right occipital region is provided – unclear whether this is intentional.	We thank the reviewer for this thoughtful comment. Although a significant group difference was also observed in the right lateral occipital cortex, its exclusion from the Abstract and the main Results text was intentional. In the present study, our a priori	-

<table>
<tr>
<td></td>
<td>hypotheses and developmental framework focused on three axes, namely late-maturing higher-order association cortices (STG and SFG), cingulate hubs supporting socioemotional and default-mode integration (ICG and PCG), and early-maturing primary sensorimotor cortices showing partial catch-up maturation (PreCG and primary visual cortex).</td>
<td></td>
</tr>
<tr>
<td>Comment 13. Introduction, Line 93: “as metrics” can be removed, as “metrics” is already specified early in the sentence.</td>
<td>Thank you for the helpful suggestion. We agree with the reviewer’s comment, and we have removed “as metrics” to avoid redundancy.</td>
<td>-</td>
</tr>
<tr>
<td>Comment 14. Methods: The sample description is dense and spans multiple paragraphs. Consider including a participant flow diagram in the Supplemental to summarize recruitment, exclusions, and final sample composition.</td>
<td>Thank you for the helpful suggestion. We agree that the sample description was dense in the Methods section. To improve clarity, we have added a participant flow diagram summarizing recruitment, exclusion, and final sample composition. This has been included as Supplementary Figure 1.</td>
<td>In the Supplementary Figure S1:
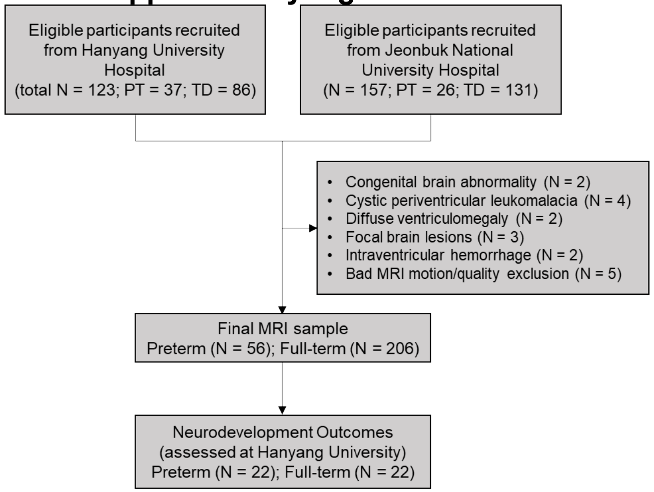

Figure S1. Flow diagram of participant inclusion and outcome availability.

An overview of cohort size, MRI eligibility, and outcome availability is summarized in Supplementary Fig. S1.</td>
</tr>
<tr>
<td>Comment 15. Methods: PNA isn’t defined in the Statistical models section.</td>
<td>Thank you for the reviewer’s attentive observation. We have now clarified the definition of PNA in the MRI acquisition subsection of the Methods to avoid ambiguity and ensure consistency in terminology throughout the manuscript.</td>
<td>In the Materials and methods section (lines 158 to 160):
Before: A total of 262 participants were recruited for the morphometric analysis using suitable MRI data obtained at 2–7 years old.</td>
</tr>
</table>

		**After:** A total of 262 participants were recruited for the morphometric analysis using suitable MRI data obtained at 1–7 years of postnatal age (PNA).
**Comment 16**. Results: A histogram of participant ages would be more informative than listing sample sizes per age bin.	Thank you for the helpful suggestion to improve the visibility of our sample characteristics. We have now generated group-specific histograms of participant age distributions, which are presented in Supplementary Figure 3 to provide a clearer overview than categorical bin counts.	**In the Supplementary Figure S3:** 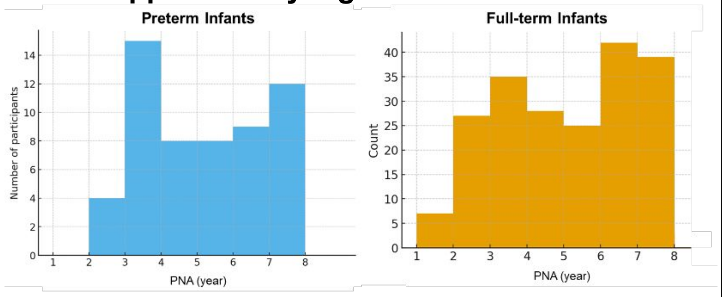 **Figure S3.** Postnatal age distribution in preterm and full-term groups. Abbreviation: PNA, postnatal age
**Comment 17**. Results: It would be helpful to use a different colour scheme to distinguish between PT > FT and FT > PT (e.g., blue and red).	Thank you for this helpful suggestion. We agree that distinct color schemes can aid in visual interpretation. In our current figure, however, the FT > PT and PT > FT contrasts are already separated into different rows/panels, allowing direct visual comparison without overlapping information. Additionally, the p-value maps follow a conventional heat-scale representation, in which warmer colors correspond to stronger significance, which was retained following internal consensus among co-authors to maintain consistency and interpretability.<br><br>Therefore, after discussion, we have decided to keep the current color scale and contrast layout unchanged while ensuring that contrast labels remain clearly indicated in the figure and caption. We appreciate the reviewer’s insight, and we hope this explanation clarifies our rationale.	-

Comment from Scientific Editorial team #3	Reply
**Comment 1**. Please add an Abbreviated Summary (up to 50 words) of your paper that captures the main purpose and conclusions of the work. The summary should state the main results written in the third person perspective (‘[1st author surname] et al. report that …’) and conclusion. This summary will be used in the contents list online and should not contain any abbreviations other than those on the accepted abbreviations list and accepted gene/protein names, making it accessible to the non-specialist. Please upload it as a Word document. Allowed abbreviations are: AIDS; ANOVA; AMPA; ATP; A,T,C,G; CNS; CSF; CT; DNA; ECG; EEG; EMG; GABA; HIV; MRI; NMDA; PET; PCR; RNA.	**Completed**
**Comment 2**. Please provide a Graphical Abstract. A graphical abstract is a visual representation of the central finding or methodology of the paper. It will be displayed in the table of contents. Authors should strive to make the graphical abstract informative, interesting, visually appealing, and straightforward (no caption needed, no abbreviations except gene names). The graphical abstract must not contain any elements for which you do not hold the copyright, such as figures or images from other articles or websites etc. The technical requirements for the graphical abstracts are:<br>- Size: 120 x 120 mm (300-600dpi) or 3000 x 3000 pixels<br>- Font: Arial 12-16 pt<br>- File type: tiff or PDF<br>- Aspect ratio: 1:1 preferred	**-**
**Comment 3**. Data availability: Thank you for providing a data availability statement. Please also provide any codes generated and used in this work within the supplementary material or through a repository, whose link has to be made available within the data availability statement.	**Completed**
**Comment 4**. Figure 1: Please ensure all annotations used in the figure are defined within the figure legend so that they may be easily interpreted as stand-alone figures.	**Completed**
**Comment 5**. Figures 2 and 4: For each statistical analysis part of a figure, please report in the figure legend the statistical test used and details of the sample size (N=) used in the statistical test.	**Completed**
**Comment 6**. Figures 2, 3, 4 and 5, Supplementary Figure S2: Please increase the font size of the text in the figures. Please also consider increasing the sizes of the subfigure panels. This is to ensure that the figures are easily viewable when fitted on a typeset journal page.	**Completed**
**Comment 7**. Figures 3, 4 and 5: Please ensure that all heatmap scale bars are labelled with titles and their appropriate units.	**Completed**

**Comment 8**. Figures 3 and 5: For each statistical analysis part of a figure, please report in the figure legend the statistical test used and details of the sample size (N=) used in the statistical test. For individual data points shown, please describe what each data point represents.	**Completed**
**Comment 9**. Figures 3 and 5, Supplementary Figure S1: Please use upper case letters (A, B, C, etc.) to label panels/ subfigures in the top left corner (no brackets or full stops) and then in the figure legend describe using the letters what is shown in each panel, unless it is self-explanatory.	**Completed**
**Comment 10**. Supplementary Figure S1: Please revisit your colour choice in this figure as the green-red combination is not colour-blind friendly.	**Completed**
**Comment 11**. Supplementary Figures S1 and S2: For each statistical analysis part of a figure, please report in the figure legend the statistical test used and relevant test statistic values alongside p values (for example F or t values for ANOVAs and t-tests). Also, please provide details of the sample size (N=).	**Completed**
**Comment 12**. Please note our policy on code availability. If any codes were generated for data analysis for this manuscript (e.g. using R, MATLAB, or similar), please make this code available either via an online repository (and provide a link within the data availability statement), or within the manuscript.	**Completed**
**Comment 13**. As a reminder at this revision stage, if you plan to edit the current figures and tables, or to make new figures and tables, please ensure that these items fit a typeset journal page (the area available for a figure or table is 170 mm x 210 mm), or are uploaded as supplementary material. Please view your figures at this size and make sure that all lettering and text are large enough to read.	**Completed**
**Comment 14**. Kindly note that if you have used BioRender software to create images in this manuscript, please provide the associated license for each image. If it is used in the graphical abstract, please add the phrase 'created in BioRender' within the image, and the link created after making the figure publicly available on the BioRender website. If it is used in a figure within the paper, please add the phrase 'created in Biorender' within the figure legend, and the link created after making the figure publicly available on the BioRender website.	**N/A**

We thank the editors and reviewers once again for the constructive review of our paper. We hope that the revised manuscript will better meet your esteemed journal's publication requirements.

Thank you very much for your valuable time and consideration.
Sincerely,
Ilwoo Lyu and Hyun Ju Lee
