# Supplementary material for: Long-term effects of preterm birth on cortical folding trajectories in early childhood
Source: Brain Commun. 2026 May 18;8(3):fcag097. doi: 10.1093/braincomms/fcag097 (PMC13181400; doi:10.1093/braincomms/fcag097)
Supplement: fcag097_Supplementary_Data [file Original Submission - Decision Letter.pdf › Original Submission - Decision Letter.pdf]

Original Submission - Decision Letter

15-Sep-2025

Dear Dr. Lee,

Thank you for your recent submission BRAINCOM-2025-821 entitled "Long-term effects of preterm birth on cortical folding trajectories in early childhood" to Brain Communications.

Your manuscript has been reviewed, and while the reviewers and editorial team appreciate many aspects of the paper, they raise some concerns that you will need to address in order for the paper to be considered for publication.

If you can address these concerns, I invite you to submit a revised manuscript and a response to each of the reviewers' comments. The reviewer(s)' comments are included at the bottom of this message.

In addition to the reviewers' comments, please also address any comments from our Scientific Editor added below to comply with our requirements for presentation of scientific data at Brain Communications. Please note we will not accept the paper unless you comply with our policies on data analysis and presentation as noted in the comments from the Scientific Editor.

Please format your revised manuscript according to our Instructions to Authors https://academic.oup.com/braincomms/pages/General_Instructions
A summary of key points is attached to help with this. Please note that there can be substantial delays to publication if you don't resubmit in line with our requirements.

To revise your manuscript, click the link below or log into https://mc.manuscriptcentral.com/braincom and enter your Author Centre, where you will find your manuscript title listed under "Manuscripts with Decisions." Under "Actions," click on "Create a Revision." Your manuscript number will be appended to denote a revision.

*** PLEASE NOTE: This is a two-step process. After clicking on the link, you will be directed to a webpage to confirm. ***

https://mc.manuscriptcentral.com/braincom?URL_MASK=cb18123193024914abdcecdf4e120326

Please upload a revised manuscript with changes tracked with track changes or in coloured text.

When submitting your revised manuscript, you will be asked to respond to the reviewer(s) comments. Please be as specific as possible in your response to all of the comments.

The editorial team at Brain Communications aims to increase transparency in scientific publishing and to demonstrate the value that peer review brings to the scientific process. Please therefore consider whether you are willing to have reviewer(s)' comments on your paper and your response published as supplementary data with the paper if it is accepted. This is entirely optional and will only be done if both the authors and reviewers agree. While submitting your revision there will be an opportunity to review or update the answer you gave previously, if necessary.

IMPORTANT: Your original files are available to you when you upload your revised manuscript. Please delete any redundant files before completing the submission.

Because we are trying to facilitate timely publication of manuscripts submitted to Brain Communications, we would appreciate your revised manuscript as soon as possible. If it is not possible for you to resubmit within 3 months, the option to submit a revision will expire, and we would then consider your revised paper as a new submission. However, if you need a little more time, please get in touch and we can extend this deadline for you.

Please note that Brain Communications is a fully open access journal, and if your article is accepted for publication, an open access charge will be payable (this does not apply to letters and scientific commentaries). Details can be found in our Instructions for Authors: https://academic.oup.com/braincomms/pages/General_Instructions

Once again, thank you for submitting your manuscript to Brain Communications and I look forward to receiving your revision.

Best wishes

Professor Tara Spires-Jones
Founding Editor, Brain Communications

Reviewer(s)' Comments to Author:

Reviewer: 1

Comments to the Author
The authors examine the long-term impact of preterm birth on cortical folding in early childhood by analyzing MRI data from 56 preterm and 206 full-term children aged 2-7 years, using local gyrification index and sulcal depth to detect microstructural and regional change. The authors' results show reductions in LGI and SD in preterm children, emphasizing the bilateral superior temporal gyrus and left superior frontal gyrus, with reversed structure-function relationships where higher folding metrics correlate negatively with neurodevelopmental outcomes like verbal comprehension and IQ, unlike in full-term peers. The authors conclude that preterm birth induces region-specific disruptions in cortical morphogenesis that affect cognition, highlighting folding-sensitive markers as potential tools for targeted interventions to improve long-term outcomes.

The paper is likely to be of interest to the neuroimaging community. However, I have several concerns that should be addressed before publication:

1. Several aspects of the methods, particularly concerning Freesurfer, are unclear or confusing.
   a. Freesurfer is generally accepted to work well on adult humans. However, this study focused on children from 2-7. How was this method verified to work well on this subject set?
   b. The calculations of SD mentions a cerebral hull surface – was this calculated using Freesurfer? If so, Freesurfer's calculations of sulcal depth are actually based on a hypothetical midcortical surface, such that gyri have negative sulcal depth. Please clarify.
   c. What cortical atlas was used for parcellation? How many regions does it include? How was it applied?
   d. L132 mentions a "shape-adaptive" LGI measure. What is this, and how was it measured?

2. The study included subjects recruited from two hospitals. However, beyond including a random effect for the hospitals, there is no discussion about the two hospitals. In particular, I am interested to know if there are any demographic, geographic, socioeconomic, etc. differences between the two that might be of interest when studying neurodevelopment?

3. Several claims in the paper were not well supported or explained.
   a. "Preterm birth not only alters folding morphology in the STG but also fundamentally reshapes the relationship between folding metrics and neurodevelopmental outcomes."
   b. "Compared to the full-term group, the preterm group exhibited lower LGI and SD in both the STG and left SFG, indicating impaired gyral and sulcal maturation, which potentially limits experience-dependent plasticity in higher-order cognitive regions."

4. A few wordings are unclear or possibly incorrect:
   a. Paragraph starting on L371: two theories of cortical folding are introduced here to explain the abnormal folding patterns in preterm children. However, these theories are not only relevant to certain regions of the brain or certain pathologies; rather, they are hypothesized to be the driving force for cortical folding throughout the brain. It might make sense to discuss how these mechanisms are perturbed or changed in premature birth, but the discussion as it stands is misleading.
   b. L16: “global” impairment, while the results showed changes in only a few select regions
   c. L71: What is meant by “asymmetrical” growth? Perhaps heterogeneous instead?

Reviewer: 2

Comments to the Author
Major Comments:
1.
The preterm group includes only 56 participants across a broad age range (2–7 years), which is a period of rapid and non-linear brain development. With such a small sample, it’s hard to know whether the study can really capture meaningful age-related differences. On top of that, only 22 preterm and 22 full-term children completed cognitive testing, yet these results are highlighted as a key finding in the abstract. These small samples challenge the generalizability of the findings, and this should be clearly acknowledged as a major limitation.

2. Relatedly, the authors treat age as a linear effect, even though early brain development is well known to follow nonlinear patterns. At the very least, the authors should explain why they chose a linear model and consider whether nonlinear terms (like quadratic or cubic) would better capture age-related effects.

3. The manuscript states that “written informed consent was obtained from all participants,” which doesn’t seem accurate given the age range. Some children were scanned as young as 2 years, and others were recruited within the first week of life. Please clarify how consent/assent was obtained from guardians and participants.

4. The authors address site effects using a covariate approach. I'm curious why more robust harmonization methods (e.g., ComBat) weren’t considered, given that it has been shown to outperform covariate adjustment (e.g., Fortin et al., NeuroImage, 2018).

5. The authors use a cluster-defining threshold of $p < 0.01$, which is more liberal than the default in SurfStat ($p < 0.001$) and the standard in surface-based neuroimaging. This choice increases the risk of false positives, as shown in Eklund et al. (2016, PNAS). At minimum, results using the more conservative threshold should be included in the supplement. If findings don’t hold up, that should be clearly stated as a major limitation.

6. The authors use Fisher’s r-to-z transformation to compare correlation strength between groups. Since linear models were already used elsewhere, it’s unclear why interaction terms weren’t used instead. That approach would allow for consistent covariate control and more interpretable results.

Minor Comments:

7. Units are missing in several places – for example, “years” for PNA, “weeks” for GA, and units for LGI/SD on color bars and axes. Please make sure all measures are clearly labeled.

8. The stated age range throughout the manuscript is 2–7 years, but the PNA plot includes values <2 years. Please clarify the discrepancy.

9. Abstract, Background: “…early extrauterine exposure…” – if this means preterm birth, consider simplifying for clarity.

10. Abstract, Methods: The claim of a more robust and accurate methodology is unclear, as SD and LGI via FreeSurfer is a standard approach for measuring cortical folding. Consider clarifying what's novel or revising the language.

11. Abstract, Results: "global impairment pattern" – consider revising, as findings are region-specific, not global.

12. Abstract, Results: all but the group difference of the right occipital region is provided – unclear whether this is intentional.

13. Introduction, Line 93: "as metrics" can be removed, as "metrics" is already specified early in the sentence.

14. Methods: The sample description is dense and spans multiple paragraphs. Consider including a participant flow diagram in the Supplemental to summarize recruitment, exclusions, and final sample composition.

15. Methods: PNA isn't defined in the Statistical models section.

16. Results: A histogram of participant ages would be more informative than listing sample sizes per age bin.

17. Results: It would be helpful to use a different colour scheme to distinguish between PT > FT and FT > PT (e.g., blue and red).

Comments from the Scientific Editorial team:

-Please add an Abbreviated Summary (up to 50 words) of your paper that captures the main purpose and conclusions of the work. The summary should state the main results written in the third person perspective ('[1st author surname] et al. report that …') and conclusion. This summary will be used in the contents list online and should not contain any abbreviations other than those on the accepted abbreviations list and accepted gene/protein names, making it accessible to the non-specialist. Please upload it as a Word document. Allowed abbreviations are: AIDS; ANOVA; AMPA; ATP; A,T,C,G; CNS; CSF; CT; DNA; ECG; EEG; EMG; GABA; HIV; MRI; NMDA; PET; PCR; RNA.

-Please provide a Graphical Abstract. A graphical abstract is a visual representation of the central finding or methodology of the paper. It will be displayed in the table of contents. Authors should strive to make the graphical abstract informative, interesting, visually appealing, and straightforward (no caption needed, no abbreviations except gene names). The graphical abstract must not contain any elements for which you do not hold the copyright, such as figures or images from other articles or websites etc. The technical requirements for the graphical abstracts are:
- Size: 120 x 120 mm (300-600dpi) or 3000 x 3000 pixels
- Font: Arial 12-16 pt
- File type: tiff or PDF
- Aspect ratio: 1:1 preferred

-Data availability: Thank you for providing a data availability statement. Please also provide any codes generated and used in this work within the supplementary material or through a repository, whose link has to be made available within the data availability statement.

-Figure 1: Please ensure all annotations used in the figure are defined within the figure legend so that they may be easily interpreted as stand-alone figures.

-Figures 2 and 4: For each statistical analysis part of a figure, please report in the figure legend the statistical test used and details of the sample size (N=) used in the statistical test.

-Figures 2, 3, 4 and 5, Supplementary Figure S2: Please increase the font size of the text in the figures. Please also consider increasing the sizes of the subfigure panels. This is to ensure that the figures are easily viewable when fitted on a typeset journal page.

-Figures 3, 4 and 5: Please ensure that all heatmap scale bars are labelled with titles and their appropriate units.

-Figures 3 and 5: For each statistical analysis part of a figure, please report in the figure legend the statistical test used and details of the sample size (N=) used in the statistical test. For individual data points shown, please describe what each data point represents.

-Figures 3 and 5, Supplementary Figure S1: Please use upper case letters (A, B, C, etc.) to label panels/ subfigures in the top left corner (no brackets or full stops) and then in the figure legend describe using the letters what is shown in each panel, unless it is self-explanatory.

-Supplementary Figure S1: Please revisit your colour choice in this figure as the green-red combination is not colour-blind friendly.

-Supplementary Figures S1 and S2: For each statistical analysis part of a figure, please report in the figure legend the statistical test used and relevant test statistic values alongside p values (for example F or t values for ANOVAs and t-tests). Also, please provide details of the sample size (N=).

-Please note our policy on code availability. If any codes were generated for data analysis for this manuscript (e.g. using R, MATLAB, or similar), please make this code available either via an online repository (and provide a link within the data availability statement), or within the manuscript.

-As a reminder at this revision stage, if you plan to edit the current figures and tables, or to make new figures and tables, please ensure that these items fit a typeset journal page (the area available for a figure or table is 170 mm x 210 mm), or are uploaded as supplementary material. Please view your figures at this size and make sure that all lettering and text are large enough to read.

-Kindly note that if you have used BioRender software to create images in this manuscript, please provide the associated license for each image. If it is used in the graphical abstract, please add the phrase 'created in BioRender' within the image, and the link created after making the figure publicly available on the BioRender website. If it is used in a figure within the paper, please add the phrase 'created in Biorender' within the figure legend, and the link created after making the figure publicly available on the BioRender website.
