# Supplementary material for: Long-term effects of preterm birth on cortical folding trajectories in early childhood
Source: Brain Commun. 2026 May 18;8(3):fcag097. doi: 10.1093/braincomms/fcag097 (PMC13181400; doi:10.1093/braincomms/fcag097)
Supplement: fcag097_Supplementary_Data [file Revision 1 - Author's Response.pdf › Revision 1 - Author's Response.pdf]

Author's Response to Decision Letter for (BRAINCOM-2025-821.R1)

Response to the Editorial Office

We thank the Editorial Office for the careful evaluation of our manuscript and for the helpful suggestions provided. We have revised the manuscript and figures accordingly, as detailed below.

[Graphical Abstract]
In response to the Editorial Office's request, we have revised the graphical abstract to improve clarity and self-contained interpretability. Additional annotations have been added to clearly convey the main research question and the key finding of the study. A concise sentence summarizing the principal result has been incorporated directly into the graphical abstract, ensuring that the central message is communicated through the visual alone, without the need for a separate caption. The revised graphical abstract is now more informative, visually clear, and aligned with the Editorial Office's guidance.

[Figure Legends and Statistical Reporting]
We have revised the legends of Figures 2–5 to ensure complete and transparent reporting of the statistical analyses. The specific statistical method used in each figure is now explicitly stated, namely linear mixed models, along with the relevant covariates included in each analysis.

In addition, the figure legends now clearly describe what each data point represents and report the number of data points included in each group. For figures displaying individual-level data, we specify that each data point corresponds to an individual infant and provide the corresponding group-specific sample sizes.

With respect to Figure 2, we identified that a description of a scatter plot was included in the legend due to a figure misidentification. This text has been removed from the Figure 2 legend and appropriately relocated to the legends of Figures 3 and 5, where the scatter plots are presented. This revision ensures consistency between the figures and their legends.

We trust that these revisions adequately address the Editorial Office's comments and improve the clarity and completeness of the manuscript.
