# Supplementary material for: Long-term effects of preterm birth on cortical folding trajectories in early childhood
Source: Brain Commun. 2026 May 18;8(3):fcag097. doi: 10.1093/braincomms/fcag097 (PMC13181400; doi:10.1093/braincomms/fcag097)
Supplement: fcag097_Supplementary_Data [file Revision 1 - Decision Letter.pdf › Revision 1 - Decision Letter.pdf]

Revision 1 - Decision Letter

03-Feb-2026

Dear Dr. Lee,

Thank you for your recent submission BRAINCOM-2025-821.R1
entitled "Long-term effects of preterm birth on cortical folding trajectories in early childhood" to Brain Communications.

I am pleased to let you know that we would like to provisionally accept your manuscript entitled "Long-term effects of preterm birth on cortical folding trajectories in early childhood" for publication in Brain Communications. There are still some minor points for you to address before final acceptance, but further peer-review will not be necessary. However, please note that we will not accept the paper unless you comply with our policies on data analysis and presentation as noted in the comments from the Scientific Editor.

Please respond to the comments shown below from reviewers and/or our Scientific Editor and submit a revised version.

Please format your revised manuscript according to our Instructions to Authors https://academic.oup.com/braincomms/pages/General_Instructions
A summary of key points is attached to help with this. Please note that there can be substantial delays to publication if you don't resubmit in line with our requirements.

To revise your manuscript, click the link below or log into https://mc.manuscriptcentral.com/braincom and enter your Author Centre, where you will find your manuscript title listed under "Manuscripts with Decisions." Under "Actions," click on "Create a Revision." Your manuscript number will be appended to denote a revision.

*** PLEASE NOTE: This is a two-step process. After clicking on the link, you will be directed to a webpage to confirm. ***

https://mc.manuscriptcentral.com/braincom?URL_MASK=f847235380f74baead114d4ffb65fb00

Please upload a revised manuscript with the changes you have made shown either with "track changes" or in coloured text.

When submitting your revised manuscript, you will be asked to respond to the reviewer(s) and editor's comments. Please be as specific as possible in your response to all of the comments.

The editorial team at Brain Communications also aims to increase transparency in scientific publishing and to demonstrate the value that peer review brings to the scientific process. Please therefore consider whether you are willing to have reviewer comments on your paper and your response published as supplementary data with the paper if it is accepted. This is entirely optional and will only be done if both the authors and reviewers agree. When submitting your revision you will have the opportunity to update the answer you entered previously.

IMPORTANT: Your original files are available to you when you upload your revised manuscript. Please delete any redundant files before completing the submission.

Because we are trying to facilitate timely publication of manuscripts submitted to Brain Communications, we would appreciate your revised manuscript as soon as possible. If it is not possible for you to submit your revision within 3 months, the option to submit a revision will expire, and we will consider your revised paper as a new submission. However, if you need a little more time, please get in touch and we can extend this deadline for you.

Please note that Brain Communications is a fully open access journal, and if your article is accepted for publication, an open access charge will be payable (this does not apply to letters and scientific commentaries). Details can be found in our Instructions for Authors: https://academic.oup.com/braincomms/pages/General_Instructions

Once again, thank you for submitting your manuscript to Brain Communications and I look forward to receiving your revision.

Best wishes

Dr. Manuela Marescotti
Scientific Editor, Brain Communications

Professor Tara Spires-Jones
Founding Editor, Brain Communications

Reviewer(s)' Comments to Author:

Reviewer: 1

Comments to the Author
The authors have addressed all of my concerns. In particular, I was not familiar with the shape-adaptive LGI, so I would like to thank the authors for introducing me to the concept. I will just note one small typo in the text added in response to comment 3a: "atypical folding patterns may [be] associated with" (the "be" is missing).

Reviewer: 2

Comments to the Author
Thank you for addressing my comments. I have no remaining concerns.

Comments from the Scientific Editor:

- Thank you for providing a graphical abstract, but please add extra annotations to make the main question and finding of the study clearer. If necessary, state the main finding with a sentence, not just a plot or image. Authors should strive to make the graphical abstract informative, interesting, and visually appealing (no separate caption is needed; abbreviations are allowed only for gene names).

- Please make sure to report in the legends of figures showing results of a statistical analysis the statistical analysis performed and not only the correction. Also please add to the these legends what each data-point represents and the n of data-points.

- Please make sure to report in the legends of figures showing a correlation the type of correlation performed.

- Fig2: please note that in the legend there is the description of a scatter plot not present in the figure, please amend either the legend or the figure.
